# Targeted enzymatic therapy for coeliac disease

**DOI:** 10.1038/s44321-026-00430-8

**Published:** 2026-05-14

**Authors:** Marina Girbal-González, Arturo Rodríguez-Banqueri, Hadeel Swaid, Soraia R Mendes, Laura Garzón-Flores, Juan Sebastián Ramírez-Larrota, Carolina Cueva, M Victoria Moreno-Arribas, Christof Regl, Christian G Huber, Katharina A Scherf, María José Rodríguez-Lagunas, Àngels Franch-Masferrer, Ulrich Eckhard, Francisco J Pérez-Cano, F Xavier Gomis-Rüth

**Affiliations:** 1 Section of Physiology; Department of Biochemistry and Physiology; Faculty of Pharmacy and Food Sciences; University of Barcelona, Av. Joan XXIII, 27-31, 08028 Barcelona, Catalonia Spain; 2 Research Institute of Nutrition and Food Safety (INSA-UB); University of Barcelona, Av. Prat de la Riba, 171, 08921 Santa Coloma de Gramenet, Catalonia Spain; 3 Proteolysis Laboratory; Department of Structural and Molecular Biology; Molecular Biology Institute of Barcelona (IBMB); Higher Scientific research Council (CSIC); Barcelona Science Park, c/Baldiri Reixac, 15-21, 08028 Barcelona, Catalonia Spain; 4 Graduate Program in Biotechnology; Faculty of Pharmacy and Food Sciences; University of Barcelona, Carrer Joan XXIII, 27-31, 08028 Barcelona, Catalonia Spain; 5 Synthetic Structural Biology; Department of Structural and Molecular Biology; Molecular Biology Institute of Barcelona (IBMB); Higher Scientific research Council (CSIC); Barcelona Science Park, c/Baldiri Reixac, 15-21, 08028 Barcelona, Catalonia Spain; 6 Institute of Food Science Research (CIAL); CSIC-UAM, c/Nicolás Cabrera, 9, 28049 Madrid, Spain; 7 Department of Biosciences and Medical Biology; Bioanalytical Research Laboratories; University of Salzburg, Hellbrunner Str. 34, 5020 Salzburg, Austria; 8 Leibniz Institute for Food Systems Biology at the Technical University of Munich, Lise-Meitner-Str. 34, 85354 Freising, Germany; 9 Technical University of Munich; TUM School of Life Sciences; Professorship of Food Biopolymer Systems, Lise-Meitner-Str. 34, 85354 Freising, Germany

**Keywords:** Digestive System, Immunology, Post-translational Modifications & Proteolysis

## Abstract

Coeliac disease (CD) is an autoimmune enteropathy triggered by proline-rich gluten immunogenic peptides (GIPs), for which no curative therapy exists. We developed *celiacase* (Clc), a recombinant prolyl endopeptidase engineered from pitcher plant neprosin to enhance expression, stability, and activity. Clc showed maximal activity at gastric pH, synergized with and resisted pepsin, and efficiently cleaved GIPs, including the highly immunogenic 33-mer, outperforming *Aspergillus niger* prolyl endopeptidase in degrading GIPs from wheat flour and gliadin. At an enzyme-to-gliadin ratio of 1:250, Clc reduced GIP levels by up to 99% in a dynamic human gastrointestinal simulator. Ex vivo, Clc-digested 33-mer fragments failed to elicit cytokine responses in mouse and rat macrophages and duodenal biopsies from CD patients. In vivo, low-dose Clc (1:75–1:380) degraded gliadin and attenuated pathology in gliadin-fed mice, reducing villus atrophy, inflammation, antibody responses, and gluten-induced dysbiosis, while restoring immune-regulatory markers and microbial metabolic pathways. In summary, Clc is a potent, acid-stable glutenase with promise as a therapeutic adjunct or alternative to a gluten-free diet for CD patients.

## Synopsis

A rationally engineered new gastric glutenase (celiacase, Clc) efficiently degrades immunogenic gluten peptides and prevents coeliac disease (CD)-associated immune, histological, and microbiota alterations across in vitro, ex vivo, and transgenic mouse models.

- Clc is a recombinant prolyl endopeptidase engineered from plant neprosin, showing increased enzymatic stability and activity ( ~ 20%) while preventing intermolecular disulfide-mediated aggregation of the parental enzyme.
- Gluten immunogenic peptides, including the CD reference 33-mer, were efficiently degraded under gastric conditions, achieving ≈99% inactivation in complex matrices and in a dynamic human gastrointestinal simulator.
- Clc-mediated cleavage of the 33-mer generated short peptides that failed to induce pro-inflammatory cytokine responses in macrophages or duodenal biopsies from CD patients.
- Oral Clc administration reduced gastrointestinal GIP levels ( ≈ 72-88%) and attenuated villous atrophy, inflammation, auto-antibody production, and gluten-induced dysbiosis in the currently most advanced transgenic mouse model for CD.
- Clc will next be evaluated as a potential oral therapy for CD in conjunction with a gluten-free diet.

## Abstract (web summary)

A rationally engineered new gastric glutenase (celiacase, Clc) efficiently degrades immunogenic gluten peptides and prevents coeliac disease (CD)-associated immune, histological, and microbiota alterations across in vitro, ex vivo, and transgenic mouse models.

## Graphical abstract

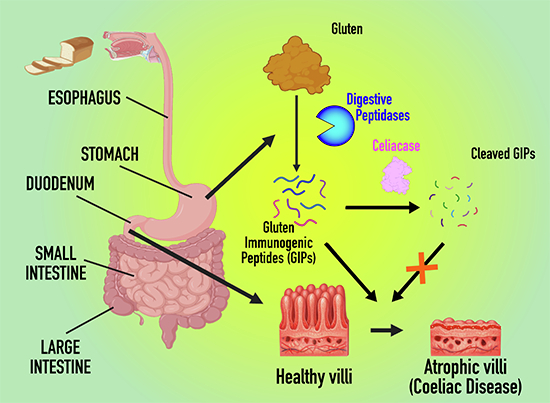

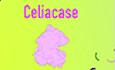

The paper explained **Medical issue**

Coeliac disease (CD) is a common autoimmune disorder triggered by the ingestion of gluten, a group of proteins present in wheat, barley, and rye. In sensitive individuals, digestion of gluten generates highly resistant fragments known as gluten immunogenic peptides (GIPs). These peptides can activate the immune system and lead to chronic inflammation and damage to the small intestinal lining, causing symptoms such as malabsorption, gastrointestinal discomfort, and systemic complications. Currently, the only effective treatment is strict lifelong adherence to a gluten-free diet (GFD). However, maintaining such a diet is challenging because gluten is widespread in processed foods and accidental exposure is frequent, meaning that many patients continue to experience symptoms despite careful dietary control.

**Results**

We developed a novel enzyme, termed celiacase (Clc), designed to break down harmful gluten peptides during digestion. The enzyme was engineered from a digestive enzyme found in carnivorous pitcher plants and optimised to improve its stability, activity, and production yield. Biochemical and structural analyses showed that Clc efficiently cleaves GIPs under acidic conditions similar to those in the human stomach. The enzyme degraded the highly immunogenic 33 amino acid peptide and reduced detectable GIP levels by up to 99% in complex substrates. When tested in immune cells and in duodenal biopsy samples from CD patients, the Clc-generated peptide fragments failed to induce the pro-inflammatory cytokine responses typically triggered by intact GIPs. The enzyme also remained active under simulated human digestive conditions, efficiently degrading gluten during the gastric phase of digestion. Oral administration of the enzyme reduced immunogenic gluten peptides in the gastrointestinal tract of standard mice. In the currently most advanced CD transgenic mouse model, which reproduces key features of the disease, Clc treatment alleviated several hallmarks of the disorder, including intestinal inflammation, villous atrophy, disease-associated antibodies, and gluten-induced alterations in gut microbiota.

**Clinical impact**

Together, these findings indicate that Clc is a potent gastric gluten-degrading enzyme capable of neutralising GIPs before they can trigger harmful immune responses. With further development and clinical evaluation, this strategy could complement the GFD and help protect individuals with CD from accidental gluten exposure.

## Introduction

Wheat has been cultivated for circa 10,000 years and is the world’s primary staple crop (de Sousa et al, 2021). The wheat kernel contains roughly 8–15% of protein, of which 85–90% is gluten, primarily composed of gliadin and glutenin (Asri et al, 2021; Biesiekierski, 2017). Along with rye secalins and barley hordeins, wheat gliadins and glutenins are classified as prolamins due to their high proline and glutamine content (Gass et al, 2006; Osborne, 1924). In the medical context, these *Triticeae* prolamins are referred to as “gluten”, which can trigger inflammatory, immunological, and autoimmune reactions in genetically predisposed individuals (Asri et al, 2021; Balakireva and Zamyatnin, 2016), collectively termed “wheat-related disorders” or “gluten-related disorders” (GRDs). These mainly include coeliac disease (CD), immunoglobulin (Ig) E-mediated wheat allergy, and non-coeliac wheat sensitivity (Asri et al, 2021; Cabanillas, 2020). CD is the most studied GRD, with a global prevalence of about 1.4% (Singh et al, 2018), and a higher relative risk and earlier onset in women (Ciacci et al, 1995; Jansson-Knodell et al, 2019). An estimated 3 million Americans have CD, diagnosed or not (Ludvigsson et al, 2025), with symptoms similar to irritable bowel syndrome, including sudden nausea, vomiting and chronic gastrointestinal disorders such as diarrhoea, nutritional deficiency, and malabsorption (Bonner et al, 2025; Tanner, 2021). Extraintestinally, CD can cause neurological and dermatological manifestations, as well as systemic effects like neurodegenerative diseases, certain lymphomas, fatigue, and headaches (Bonner et al, 2025). A daily gluten intake of ≈10 mg can already trigger the disease (Monachesi et al, 2021), which is only around 0.1% of the gluten found in a typical Western diet (5–20 g/day) (Biesiekierski, 2017). There is no medicinal treatment for CD, and patients must adhere to a lifelong gluten-free diet (GFD) to alleviate symptoms (Cabanillas, 2020). However, a GFD often lacks balanced nutrition (Balakireva and Zamyatnin, 2016; Zis and Hadjivassiliou, 2019) and can lead to microbiome dysbiosis (Chibbar and Dieleman, 2019). Additionally, gluten is prevalent in most processed foods and drugs in Western societies, so two-thirds of CD patients on a GFD are inadvertently exposed to gluten (Silvester et al, 2020). Consequently, about half of patients on a GFD continue to experience symptoms, particularly among those with refractory and non-responsive CD (Bonner et al, 2025).

The aetiological agents of CD are gluten immunogenic peptides (GIPs) derived from *Triticeae* prolamins (Rodríguez-Ramírez et al, 2024), which, unlike most food proteins, are not fully digested by the peptidases from the gastrointestinal tract (GIT) due to their high proline content (roughly 35%) (Bonner et al, 2025). This leads to the accumulation of 2–4 kDa GIPs in the chyme (Rodríguez-Ramírez et al, 2024; Shan et al, 2002; Tye-Din et al, 2010), which are translocated from the small intestinal lumen to the underlying lamina propria through the intestinal epithelial barrier, which is formed by tightly joined enterocytes (Cabanillas, 2020; Rodríguez-Ramírez et al, 2024). In the lamina propria, some GIP glutamines are deamidated to glutamate by transglutaminase 2 (TG2) (Zhuang and Khosla, 2020) and subsequently bind to antigen-presenting cells (APCs)—dendritic cells, macrophages, and B cells (Wei et al, 2020)—previously activated by pro-inflammatory cytokines such as interleukin (IL)-15 and type-1 interferons (IFNs). APCs utilise class-II human leucocyte antigen (HLA) receptors, primarily the DQ2.5 and DQ8 haplotypes, which preferentially bind deamidated peptides (Cabanillas, 2020; Wei et al, 2020). These CD-associated haplotypes are necessary but not sufficient for the disease, as they are shared by about 40% of the general population (Asri et al, 2021; Kim et al, 2004; Rej & Sanders, 2021; Tinto et al, 2015). Over 50 GIPs have been identified in the context of CD (Wei et al, 2020), including a 33-residue peptide (33-mer) from α-gliadin (Shan et al, 2002; Tye-Din et al, 2010) and a 26-mer from γ-gliadin (Shan et al, 2005; Sjöström et al, 1998), which are among the most immunogenic. These peptides contain six and five overlapping nine-residue immunogenic epitopes, respectively (Shan et al, 2002; Shan et al, 2005; Wei et al, 2020). Notably, a deletion mutant of wheat α2-gliadin lacking only the 33-mer was not toxic, establishing it as a reference standard in CD research (Shan et al, 2002; Shan et al, 2005).

Activated APCs present HLA-bound GIPs to T cell receptors on naïve gluten-specific CD4^+^ T cells, leading to their activation (Cabanillas, 2020; Wei et al, 2020). This process initiates both innate and adaptive immune responses, resulting in the secretion of pro-inflammatory cytokines like IFN-γ, IL-2, and IL-21 as part of a response by T-helper type-1 (Th1) cells. A further response by T-helper type 2 (Th2) cells is also triggered through the production of IgA and IgG antibodies (Abs) against gliadin, deamidated GIPs, endomysium, and TG2, as well as the activation of intestinal fibroblasts to remodel the lamina propria and the licensing of CD8^+^ intraepithelial lymphocytes (IEL) towards a cytotoxic phenotype (Cabanillas, 2020; Voisine and Abadie, 2021; Wei et al, 2020). This cascade leads to shedding and epithelial damage, including villous atrophy and crypt hyperplasia, which reduces the absorptive surface of the intestine. This understanding has led to a diagnostic protocol for CD based on upper GIT endoscopy with duodenal or jejunal biopsy to evaluate the presence of IELs and the extent of villous blunting and hyperplasia, assessed by the Marsh classification (Ensari and Marsh, 2019) with the Oberhuber correction (Oberhuber et al, 1999). Additional screening includes HLA genotyping and serological tests for Abs against TG2, endomysium, and deamidated GIPs (Besser and Khosla, 2023; Cabanillas, 2020; Rispo et al, 2024). Unfortunately, despite these diagnostic tools and an increasing global incidence (King et al, 2020; Roberts et al, 2021), gastrointestinal symptoms may persist for an average of around 11 years before diagnosis, with up to half of CD patients persisting undiagnosed (Tanner, 2021).

To study the processes of CD, various in vitro systems have been developed, including human gut-derived organoids (Freire et al, 2019), organ-on-a-chip technology (Moerkens et al, 2019), and dynamic gastrointestinal simulators (Barroso et al, 2015). CD is also investigated using preclinical animal models (Freitag et al, 2009; Štěpánková et al, 1996; Troncone and Ferguson, 1988), particularly humanised models that are transgenic for several human genes relevant to the disease, such as NOD.DQ8/Aβ^0^ (Galipeau et al, 2011), hCD4.DR3-DQ2.MHCII^Δ/Δ^ (de Kauwe et al, 2009), and DQ8-D^d^-IL-15tg (DePaolo et al, 2011) mice. However, these models have not successfully recapitulated CD until recently, when Abadie and colleagues modified the DQ8-D^d^-IL-15tg model to overexpress IL-15 in intestinal epithelial cells under the villin promoter, resulting in the DQ8-D^d^-villin-IL-15tg model (Abadie et al, 2022; Abadie et al, 2020). These mice exhibit the HLA-DQ8 haplotype, overexpress IL-15 and IFN-γ in the gut epithelium and lamina propria, produce IgG and IgA Abs against gliadin, present increased IEL infiltration, and develop villous atrophy after gluten challenge without any adjuvant. Importantly, symptoms revert upon adherence to a GFD or administration of TG2 inhibitors. Overall, these findings have formally demonstrated that gluten intake, susceptible HLA haplotypes, activated TG2, and the induction of adaptive immunity via CD4^+^ T cells are necessary and sufficient for causing CD (Abadie et al, 2022; Abadie et al, 2020; Voisine and Abadie, 2021).

Current therapeutic development for CD includes endopeptidases, known as “glutenases” (Gass et al, 2006), for oral enzyme therapy (Besser and Khosla, 2023; Cabanillas, 2020; Marti et al, 2005; Shan et al, 2002; Shan et al, 2005; Wei et al, 2020). A glutenase intended for clinical use must effectively function in the stomach, staying stable and highly active in the fasted gastric environment (pH ≈1.9 (Abuhelwa et al, 2016)), thus mimicking the pH-profile of gastric pepsins while resisting their activity. It should exhibit prolyl endopeptidase (PEP) or glutaminyl endopeptidase activity to thoroughly degrade immunogenic epitopes within GIPs alongside pepsin, even in the presence of large food quantities. Importantly, the glutenase must not harm intestinal tissues or disrupt nutrient absorption after gastric emptying, staying inactive at the postprandial pH values of the small intestine and colon (pH ≈6.5–6.7) (Bonner et al, 2025; del Amo-Maestro et al, 2022; Shan et al, 2002; Tanner, 2021; Wei et al, 2020; Zhang et al, 2023). Potential glutenases under investigation originate from bacteria, fungi, or plants and involve serine peptidases such as *Aspergillus niger* PEP (AN–PEP) (König et al, 2017; Liu et al, 2021; Marti et al, 2005; Mitea et al, 2008; Salden et al, 2015; Shan et al, 2004; Shan et al, 2002; Stefanolo et al, 2024; Stepniak et al, 2006; Wei et al, 2020) and aspartate peptidases (Ehren et al, 2009; Rey et al, 2016; Zhang et al, 2023). In particular, AN–PEP may have a positive impact on CD symptom intensity and quality of life during long-term GFD (Stefanolo et al, 2024). Other enzymes being explored are a serine-class dipeptidyl-peptidase IV, a serine-carboxyl peptidase, and cysteine peptidases like caricain from *Carica papaya* and EP-B2 from barley (Bonner et al, 2025; Cavaletti et al, 2019; Ehren et al, 2009; Gass et al, 2006). Notably, the serine peptidase kumamolysin from *Alicyclobacillus sendaiensis* has undergone multiple generations of computational redesign and optimisation (Kuma030, Kuma062, TAK-062, and KumaMax) (Pultz et al, 2021; Pultz et al, 2020). Despite these advancements, none of these candidates or their combinations, such as STAN1 (Ehren et al, 2009) and latiglutenase (Murray et al, 2022), have fully met the criteria for a true therapeutic glutenase, and clinical trials have not demonstrated sufficient remission in CD patients to replace a GFD (Bonner et al, 2025; Kivelä et al, 2021; König et al, 2017; Lähdeaho et al, 2014; Murray et al, 2022; Pultz et al, 2021; Tack et al, 2013; Wei et al, 2020), although AN–PEP has been approved as an alimentary supplement (Stefanolo et al, 2024). Thus, there is a pressing need to continue developing effective glutenases for CD (Bonner et al, 2025; Rej and Sanders, 2021; Wei et al, 2020).

Neprosin (Np), a digestive peptidase from the carnivorous *Nepenthes* plant, has emerged as a promising glutenase, particularly in combination with other peptidases (Lee et al, 2016; Rey et al, 2016; Schräder et al, 2017; Schräder et al, 2018), as well as a reagent for gluten-safe food production in bioengineered yeast (Ting et al, 2026). We have previously developed a high-yield mammalian expression system for Np, characterising it structurally and mechanistically (del Amo-Maestro et al, 2022). This glutamate-class PEP, distinguished by two unique catalytic glutamates, is synthesised as a zymogen that autoactivates at low pH. Mature Np effectively degrades gliadin and the 33-mer in vitro, and preliminary studies in standard C57BL/6 mice have shown that co-administering gliadin with minute amounts of the Np zymogen, pro-neprosin (pNp), significantly reduces GIP levels in the small intestine (del Amo-Maestro et al, 2022).

In this study, we developed an enhanced Np variant, designated celiacase (Clc), via structure-guided mutagenesis. Its glutenase activity was comprehensively characterised by structural, functional, and biophysical analyses, benchmarked against AN–PEP, and validated in a dynamic gastrointestinal simulator replicating the human GIT. Efficacy was further assessed in mouse and rat macrophages and duodenal biopsies from CD patients, as well as in C57BL/6J mice and the transgenic DQ8-D^d^-villin-IL-15tg model, with a focus on its capacity to prevent hallmark CD features, including villous atrophy, disease-specific Abs, and other biomarkers. In the transgenic model, we additionally profiled gut microbiota composition and functionality, together with lymphocytic phenotype, under conditions with and without Clc treatment.

## Results

### Design of celiacase as a novel glutenase candidate

**Figure 1:**
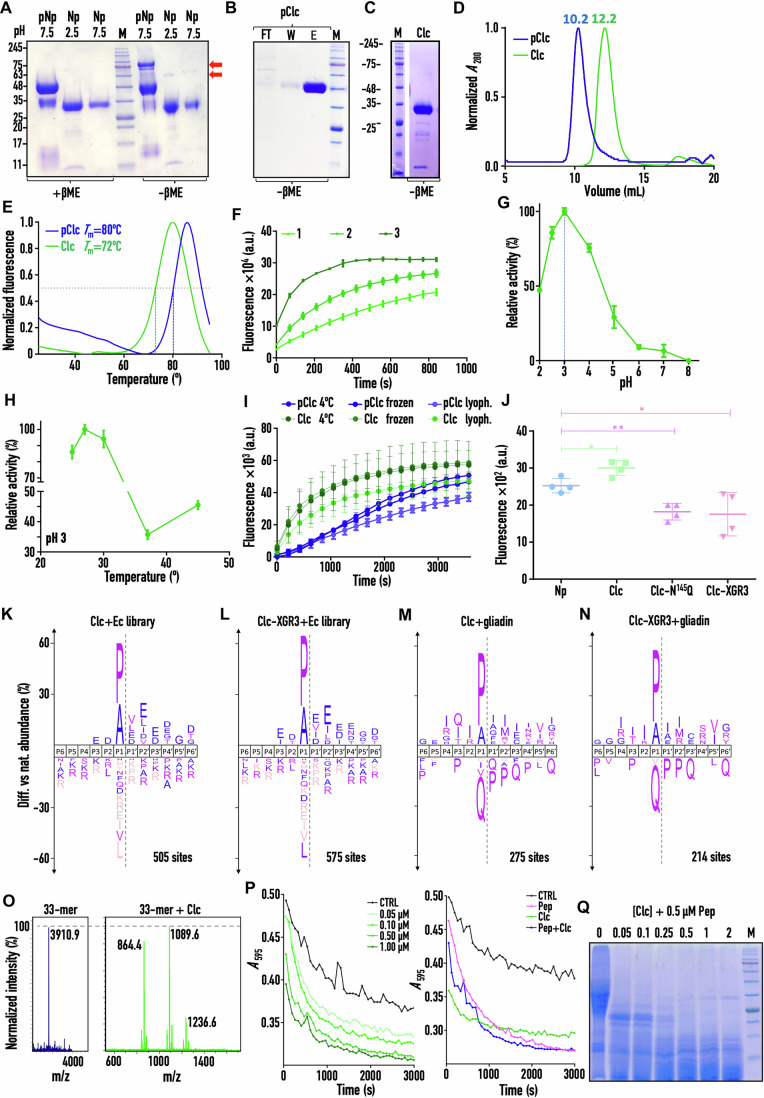
Molecular characterisation of neprosin and celiacase. (**A**) Oligomerisation analysis of pro-neprosin (pNp) and neprosin (Np) via SDS–PAGE under reducing (+βME) and non-reducing (–βME) conditions using β-mercaptoethanol. Red arrows pinpoint disulfide-linked pNp and Np dimers. (**B**) Non-reducing SDS–PAGE of the flowthrough (FT), wash (W), and elution (E) steps during IMAC purification of pro-celiacase (pClc). (**C**) Celiacase (Clc) obtained by autolytic maturation and pro-domain removal of pClc at pH 2.5. (**D**) SEC profiles of pClc and Clc. (**E**) Normalised differential scanning fluorimetry profiles of pClc and Clc, with corresponding temperatures of mid-transition (*T*_m_). (**F**) Concentration-dependent Clc activity at 0.036 µM (1), 0.072 µM (2), and 0.36 µM (3) measured at pH 3 against the FS6-QPQL quenched-fluorescent peptide substrate (Mca–Q–P–Q–L–Dpa–A–R–NH_2_) at 10 µM. (**G**) Clc pH-dependence in front of FS6-QPQL; *n* = 3. (**H**) Temperature dependence of Clc activity in front of FS6-QPQL at pH 3, measured at 25, 27, 30, 37 and 45 °C; *n* = 3. (**I**) Activity of Clc (green) and pClc (blue) at pH 3 against FS6-QPQL after storage at 4 °C for one week, flash-freezing/thawing, or lyophilisation/reconstitution. Zymogen activity curves show a sigmoidal profile with an initial lag phase, indicating autolytic activation; *n* = 3. (**J**) Activity of Np, Clc, glycosylation mutant Clc–N^145^Q, and multiple mutant Clc–XGR3 (section “Protein production and purification”) against the FS6-QPQL fluorogenic peptide. Statistics analysis of the activity, comparison via Student’s *t*-test: *p ≤ 0.05, **p ≤ 0.01, ***p ≤ 0.001 (Clc vs Np, *p* = 0.0142; Clc-N^145^Q vs Np: *p* = 0.0031; Clc–XGR3 vs Np: *p* = 0.0464); *n* = 3. (**K**, **L**) Substrate specificity profiling of Clc and Clc–XGR3 using PICS (Proteomics-based protease specificity profiling, mass spectrometry, and data analysis) with an *Escherichia coli*-derived tryptic library, compared to amino-acid abundance in the *E. coli* K12 proteome, and *iceLogo* visualisation. (**M**,** N**) PICS of Clc and Clc–XGR3 using commercial gliadin and comparing to the GluPro cereal prolamin database. (**O**) MALDI-TOF mass spectra of the intact 33-mer (left; 3911 Da) and after 20 min incubation with Clc (right). Observed peaks correspond to the sodium adducts of the resulting fragments after hydrolysis at QP–QL/P sites (see also Appendix Fig. S1A, B). (**P**) Time-dependent turbidimetry of commercial gliadin (Control) with Clc at various concentrations (left panel) or with pepsin (Pep)/Clc+Pep (at 0.5 µM) (right panel). (**Q**) SDS–PAGE of commercial gliadin degradation by pepsin (at 0.5 µM) and increasing Clc concentrations (in µM). Data in panels (**F**–**I**) are presented as mean ± SEM. Source data are available online for this figure.

Purified pNp and Np consistently formed dimers at pH 7.5, as shown by SDS–PAGE (Fig. 1a). This indicated intermolecular disulfide bond formation that led to aggregation, precipitation, and 20–30% protein loss—especially during concentration. Structural reanalysis of pNp and Np (Protein Data Bank [PDB] entries 7ZU8, 7ZVA, 7ZVB, 7ZVC (del Amo-Maestro et al, 2022)) identified two disulfide bridges (C^219^–C^224^ and C^358^–C^379^; numbering per UniProt C0HLV2) and a non-conserved, buried cysteine at position 334. It has a surface-exposed backbone and double side-chain occupancy, which suggests susceptibility to aberrant disulfide bonding upon minor conformational shifts. To prevent this, we introduced a C^334^V mutation—giving rise to a zymogen variant designated pro-celiacase (pClc)—which, based on in silico modelling, avoided steric clashes and yielded good packing. We also mutated the highly exposed *N*-glycosylation site at N^145^ (variant pClc–N^145^Q) to potentially improve production yield. A third variant, pClc–XGR3, was designed via sequence alignment with the *Nepenthes alata* orthologue (UniProt A0A1L7NZU4) and expert-guided modelling. This construct incorporated 60 surface mutations and an eight-residue loop deletion (ΔY^78^–Y^85^) (see Section 4.1), with the aim of testing structural stability without affecting catalytic or substrate-binding regions.

Expression in Expi293F cells (del Amo-Maestro et al, 2022) resulted in a 5–10% yield increase for pClc compared with pNp, whereas pClc–N^145^Q and pClc–XGR3 showed slightly reduced yields. Notably, pClc–XGR3 remained expressible despite extensive surface modifications. Neither pClc nor Clc formed intermolecular disulfides (Fig. 1B,C), and size-exclusion chromatography (SEC) confirmed monomeric elution profiles (Fig. 1D). Clc was generated from pClc by autolytic maturation at pH 2–3 (Fig. 1C,I), with the yield of purified Clc improving by around 20% over Np. Differential scanning fluorimetry indicated that the C^334^V mutation preserved thermostability, as Clc and pClc exhibited slightly higher mid-transition temperatures (72 and 80 °C; Fig. 1E) than those reported for Np and pNp (68 and 77 °C, respectively) (del Amo-Maestro et al, 2022). In summary, Clc prevents Np oligomerisation and enhances yield and stability, underscoring its greater potential for application.

### In vitro peptidolytic activity and substrate specificity

**Figure EV1:**
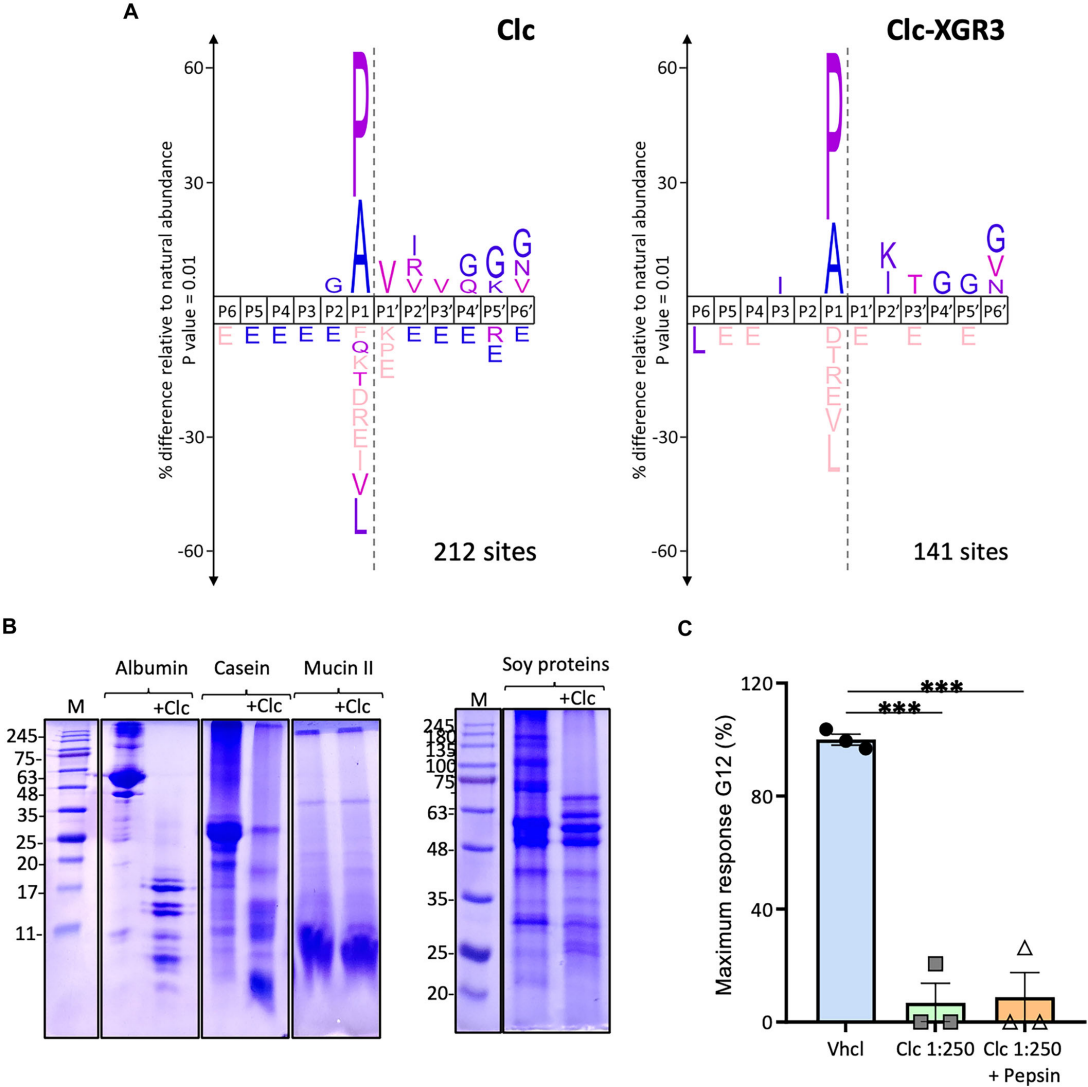
Specificity profiling of celiacase and mutant Clc–XGR3 using PICS proteomics and activity against other proteins. (**A**) The substrate specificities of celiacase (Clc) and Clc multimutant Clc–XGR3 were characterised using a GluC-derived peptide library from *Escherichia coli*. *IceLogos* visualise statistically significant (*p* < 0.01) amino-acid enrichment (above the x-axis) and depletion (below the x-axis) at non-prime (**P**_**6**_–**P**_**1**_, left) and prime (**P**_**1**_**’**–**P**_**6**_**’**, right) positions relative to the cleavage site, based on amino-acid frequencies in the *E. coli* K12 proteome. The data are derived from semi-specific cleavage peptides identified with at least two peptide-spectrum matches, and pink letters indicate amino acids not detected at a given position. (**B**) SDS–PAGE of albumin, casein, mucin II, and soy proteins after degradation by Clc at a 1:420 enzyme:substrate ratio. (**C**), ELISA (*n *= 3) of complex-meal digestion products using the G12 mAb pair, comparing Clc (1:250) and Clc (1:250) + Pepsin (0.5 µM) with control (Vhcl). Data were presented as mean ± SEM. Statistical analysis via one- or two-way ANOVA: *p ≤ 0.05, **p ≤ 0.01, ***p ≤ 0.001.

To evaluate Clc activity in vitro, we used a fluorogenic peptide incorporating the QPQL motif, which is recalcitrant to digestive peptidases (FS6-QPQL). It is present in the 33-mer standard and other GIPs (as QPQL/F/Q), including a 17-mer and 25-mer from α-gliadin, a 20-mer and 26-mer from γ-gliadin, a 17-mer from ω-gliadin, and a 16-mer from secalin (de Kauwe et al, 2009; Shan et al, 2002; Shan et al, 2005; Sjöström et al, 1998; Tye-Din et al, 2010; van de Wal et al, 1998). Clc efficiently cleaved this substrate in a concentration-dependent manner (Fig. 1F), with optimal activity at pH 3 and within the acidic range of pH 2–4 (Fig. 1G), mimicking gastric conditions. Activity dropped by about 80% at pH 5 and was *de facto* abolished at pH 6–7. Clc remained active between 25–45 °C, peaking at 27 °C (Fig. 1H), and retained activity after storage (as either pClc or Clc) at 4 °C, –80 °C, or in lyophilised form (Fig. 1I). Clc exhibited around 20% higher activity than Np and also outperformed both Clc–N^145^Q and Clc–XGR3 (Fig. 1J). Substrate specificity profiling via PICS proteomics (see section “Proteomics-based protease specificity profiling, mass spectrometry, and data analysis”) with various peptide libraries and substrates identified hundreds of cleavage sites and confirmed Clc’s preference for proline at the **P**_**1**_ position (Figs. 1K,M and EV1A). This is consistent with its classification as a PEP and its efficient cleavage of FS6-QPQL. Notably, Clc–XGR3 retained identical specificity—shared with Np (del Amo-Maestro et al, 2022)—despite extensive mutagenesis (Figs. 1L,N and EV1A). Finally, MALDI-TOF mass spectrometry (MS) confirmed efficient cleavage of the 33-mer by Clc at three QP–QL/P sites at pH 3, generating 9-, 7- and 3-mer fragments (Fig. 1O; Appendix Fig. S1), which were used in ex vivo assays (vide infra). These findings establish Clc as a potent, specific, and robust PEP targeting GIP motifs under physiologically relevant acidic conditions while staying inactive at the pH of the postprandial small intestine and colon.

### In vitro cleavage of gluten immunogenic peptides in complex matrices

To evaluate Clc’s ability to degrade GIPs in complex substrates, we employed commercial ELISA kits using two complementary mAb pairs, G12 and R5 (see section “Enzyme-linked immunosorbent assay quantification of gluten immunogenic peptides”), both widely recognised as standards in CD research (Morón et al, 2008a; Osman et al, 2001). G12 targets the QPQLPY epitope of the 33-mer and related motifs (e.g. QPQLPY, QPQQPY, QPQQPF, QPQLPF, QPQLPL and QPELPY) present in wheat, barley, and rye prolamins (Morón et al, 2008b). As expected, G12 detected the intact 33-mer but not its Clc-generated fragments cleaved at QP–QL sites. In contrast, R5 recognises the QQPFP motif—absent from the 33-mer but abundant in α- and γ-gliadins and ω-secalins—as well as QQQFP, LQPFP, and QLPFP (Morón et al, 2008b; Osman et al, 2001; Tanner, 2021). R5 is the Codex Alimentarius reference for gluten quantification in “gluten-free” products (Cagnasso et al, 2023; Tanner, 2021).

**Figure 2:**
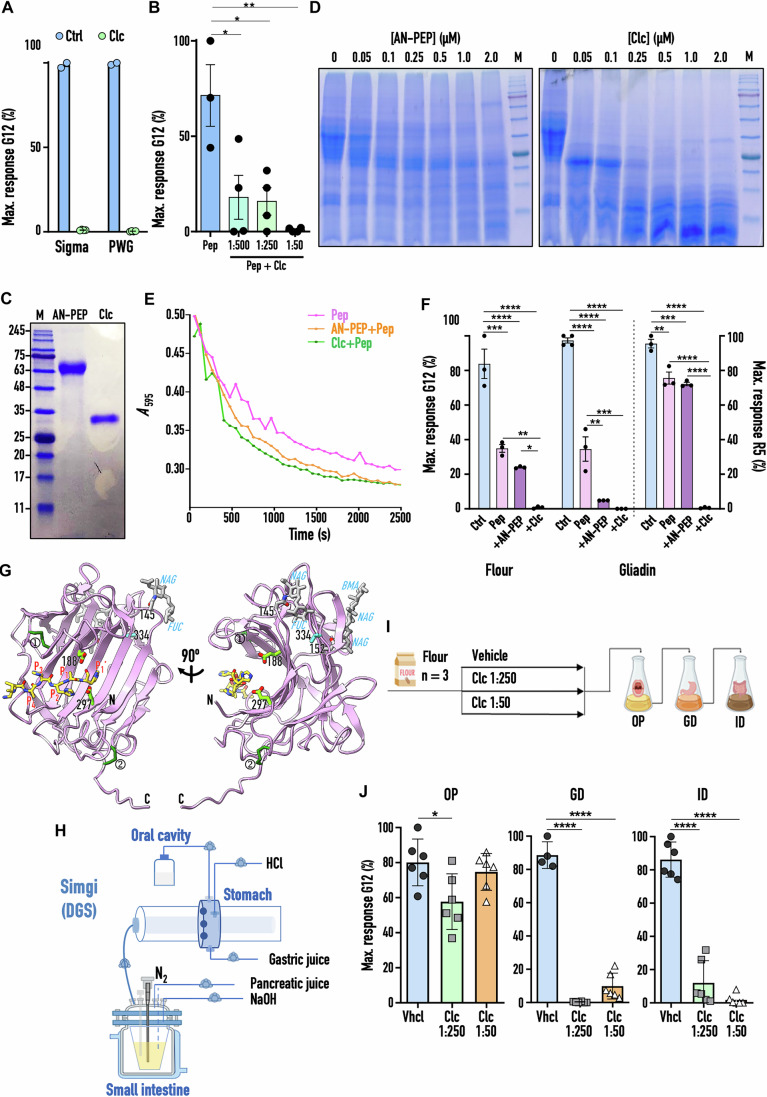
Activity in complex matrices and setups, molecular structure, and benchmarking in vitro. (**A**) ELISA of commercial gliadin (from Sigma) (*p* = 4.39E-08) and PWG-gliadin (*p* = 1.33E-09) after cleavage by Clc at 1:320 (total product weight; corresponds to 1:305 and 1:350 gliadin content, respectively), using the G12 mAb pair against toxic GIPs (*n* = 2 or 4/group) (Morón et al, 2008b). Controls (Ctrl) lacked the enzyme. (**B**) ELISA with the G12 mAb of wheat flour (*Farina de Girona*) digested by pepsin (Pep; 0.5 µM) alone and with Clc at 1:500 (*p* = 0.019 vs. Ctrl), 1:250 (*p* = 0.014 vs. Ctrl), or 1:50 (*p* = 0.003 vs. Ctrl) (*n* = 4/group). (**C**) SDS–PAGE of purified AN–PEP from commercial GiadinX capsules and mature Clc. (**D**) SDS–PAGE of commercial gliadin degradation by increasing concentrations of AN–PEP (left) or Clc (right). (**E**) Time-dependent turbidimetry of commercial gliadin degradation by pepsin (0.25 µM) alone, pepsin with AN–PEP (both at 0.25 µM), and pepsin with Clc (both at 0.25 µM). (**F**) ELISA of digestion products from wheat flour (*Farina de Girona*) and commercial gliadin, detected with the G12 mAb (left of the dashed line) or R5 mAb (right). Samples were digested with pepsin (0.5 µM) (*p* = 0.0003 vs. Ctrl, *p* = 0.003 vs. Clc), AN–PEP (1:250 enzyme:gliadin ratio) + pepsin (0.5 µM) (*p* = 0.00007 vs. Ctrl, *p* = 0.035 vs. Clc), or Clc (1:250) + pepsin (0.5 µM) (*p* = 5.7E-06 vs. Ctrl) for flour, and with pepsin (0.5 µM) (G12: *p* = 1.49E-06 vs. Ctrl, *p* = 0.001 vs. AN–PEP, *p* = 0.0004 vs. Clc; R5: *p* = 0.001 vs. Ctrl, *p* = 5.56E-08 vs. Clc), AN–PEP (1:320) + pepsin (0.5 µM) (G12: *p* = 5.04E-08 vs. Ctrl; R5: *p* = 0.0004 vs. Ctrl), or Clc (1:320) + pepsin (0.5 µM) (G12: *p* = 3.22E-08 vs. Ctrl; R5: *p* = 8.62E-09 vs. Ctrl) for commercial gliadin. Controls (Ctrl) lacked enzymes (*n* = 3/group). (**G**) Ribbon plot of the crystallographic catalytic domain structure of Clc, showing β-strands as arrows, in two orthogonal views. The C-terminal purification tag protrudes from the surface (bottom) and inserts into a symmetric molecule, mimicking a substrate (stick model with carbons in gold) spanning positions **P**_**5**_–**P**_**1**_ + **P**_**1**_**’** at neutral pH. The scissile bond is normally cleaved at acidic pH by catalytic glutamates E^188^ and E^297^ (see also (del Amo-Maestro et al, 2022) for the detailed chemical mechanism). Two surface asparagines are *N*-glycosylated: N^145^ (β1,4 → NAG–α1,6 → FUC) and N^152^ (β1,4 → NAG–β1,4 → NAG–β1,4 → BMA) (see Appendix Table S1 for sugar abbreviations). The catalytic domain is stabilised by two disulfides (①, C^219^–C^224^ and ②, C^358^–C^379^), and the characteristic V^334^ of celiacase (C^334^ in wild-type neprosin (del Amo-Maestro et al, 2022)) is shown with carbons in cyan. (**H**) Schematic of the “Simgi^®^” dynamic gastrointestinal simulator (DGS), which models the oral-phase, stomach, and small intestine of the human GIT, along with input injection points. For the physical setup, see Appendix Fig. S4. (**I**) Experimental setup for evaluating Clc activity against wheat flour (*Farina de Girona*) in the DGS. Each condition—control (no Clc; referred to as “vehicle” [Vhcl]), Clc 1:250, and Clc 1:50—was tested in triplicate. Samples were collected at the oral-phase (OP) stage, and the gastric (GD) and intestinal (ID) digestion stages. (**J**) ELISA of OP, GD, and ID digestion products using G12 mAb, comparing Clc 1:250 (OP: *p* = 0.034 vs. Vhcl, GD: *p* = 3.00E-11 vs. Vhcl, ID: *p* = 5.03E-09 vs. Vhcl) and Clc 1:50 (GD: *p* = 1.30E-10 vs. Vhcl, ID: *p* = 8.23E-10 vs. Vhcl) with control (Vhcl; *n* = 6/group, three replicates of two independent experiments). Results are expressed as mean ± SEM. Statistical differences: *p ≤ 0.05, **p ≤ 0.01, ***p ≤ 0.001 and ****p ≤ 0.0001. For data following a normal distribution and showing homogeneity of variance (**B**, **F**, **J**), one-way *ANOVA* followed by a Bonferroni post hoc test was performed. For (**A**), data were analyzed using the Kruskal–Wallis test followed by Dunn’s multiple-comparisons test with Bonferroni correction. Source data are available online for this figure.

For substrates, we used a commercial gliadin preparation, which we found contained 765 mg/g gliadin and 83 mg/g glutenin (Appendix Fig. S2), consistent with previous analyses (Xhaferaj and Scherf, 2024), and the PWG-gliadin standard from the Working Group on Prolamin Analysis and Toxicity, which contains 864 mg/g gliadin (van Eckert et al, 2006). Clc degraded commercial gliadin in a concentration-dependent manner, as shown by turbidimetry (Fig. 1P), and acted synergistically with gastric pepsin, exhibiting optimal activity at 0.25–0.5 µM Clc and 0.5 µM pepsin (Fig. 1P,Q; Appendix Fig. S3C,D). G12-based ELISA confirmed ≈99% GIP inactivation in both gliadin products (Fig. 2A) at a 1:320 w/w enzyme:gliadin ratio. Unless otherwise indicated, all enzyme:substrate ratios hereafter are expressed as w/w and normalised to true gliadin content (Appendix Fig. S2 and (van Eckert et al, 2006)). We also analysed a local wheat flour containing 50 mg/g gliadin, 28 mg/g glutenin and 21 mg/g albumin/globulin (Appendix Fig. S2), values consistent with typical wheat flours (Schuster et al, 2022). Clc, at ratios of 1:500, 1:250 and 1:50 (with pepsin), reduced GIP levels in a dose-dependent manner, achieving near-complete removal (≈98%) at 1:50 (Fig. 2B). Moreover, we evaluated the activity of Clc in the context of a complex meal, with or without pepsin, observing similar results (Fig. EV1C). This underscores Clc’s potency despite the substantial buffering capacity of the matrix, which contains proteins, starch, fibre, lipids, enzymes, inhibitors, vitamins and minerals (McCance et al, 1945). In addition, Clc demonstrated activity against representative food substrates tested individually, including albumin, casein and soy proteins (Fig. EV1B). Importantly, the absence of off-target activity against gastric mucin II, which lines the stomach, supports gastric mucosal compatibility (Fig. EV1B).

For benchmarking, Clc was compared with AN–PEP, a candidate glutenase from a commercial dietary supplement (Stepniak et al, 2006; Tanner, 2021), whose purity was confirmed (Fig. 2C). The enzyme was titrated with a specific PEP nitroanilide substrate, confirming full functionality of the preparation (Appendix Fig. S3A). High activity was further verified using the fluorogenic FS6-QPQL substrate (Appendix Fig. S3B). However, under simulated gastric conditions, Clc consistently outperformed AN–PEP in gliadin degradation, both alone (Fig. 2D) and in combination with pepsin (Fig. 2E). At 1:500, Clc removed ≈99% of GIPs from wheat flour and gliadin, as detected by G12, compared with ≈66% by AN–PEP. R5-based assays corroborated Clc’s superior efficacy and revealed that AN–PEP performs only marginally better than pepsin alone (Fig. 2F).

Collectively, these findings demonstrate that Clc efficiently degrades GIPs in vitro within complex food matrices, acts synergistically with pepsin under acidic conditions while resisting pepsin-mediated degradation, and outperforms other candidate glutenases.

### Molecular structure of celiacase

To gain insight into the molecular determinants of Clc function, we determined its X-ray crystal structure (Fig. 2G; Appendix Table S1). Notably, whereas Np proved challenging to crystallise, Clc readily yielded high-quality crystals under multiple conditions, reflecting its enhanced stability and conformational homogeneity. As inferred from pNp (del Amo-Maestro et al, 2022), pClc consists of a pro-domain (R^25^–P^128^), which governs folding and latency, and a catalytic domain (S^129^–Q^380^). The C-terminal segment of the pro-domain (L^113^–P^128^) is flexible and occupies the catalytic cleft in a substrate-like conformation, thereby blocking access. Autolytic cleavage at P^128^–S^129^ in *trans* releases the mature, active Clc enzyme. The catalytic domain adopts an antiparallel β-sandwich architecture, comprising a seven-stranded, highly curled front sheet and an eight-stranded back sheet, which are connected by nine crossover loops and stabilised by two disulfide bonds. Two *N*-glycosylation sites were identified at N^145^ and N^152^. The front sheet forms a narrow channel that accommodates extended peptide substrates (Fig. 2G, right), with the C-terminal purification tag inserting into the active site of a crystallographic symmetry mate. This mimics substrate binding and reveals two co-catalytic glutamates, E^188^ and E^297^, originating from the front sheet (Fig. 2G, left). The cleft is ideally configured to accommodate QPQL-containing peptides for cleavage. Collectively, these features classify Clc—and Np—as members of the eelysin family within the glutamate peptidase class (del Amo-Maestro et al, 2022; Kondo et al, 2010; Sims et al, 2004). Overall, Clc exhibits a compact, robust fold that confers pH stability, resistance to pepsin digestion, and specificity for QPQL motifs such as those present in the 33-mer.

### Wheat flour digestion by celiacase in a simulated human gastrointestinal tract

To assess Clc activity under physiologically relevant conditions, we employed a dynamic gastrointestinal simulator (Simgi^®^), a computer-controlled in vitro model that replicates human digestion (see section “Studies in a dynamic gastrointestinal simulator”; Fig. 2H–J; Appendix Fig. S4) (Cueva et al, 2015) and has been used to study AN–PEP in the past (Mitea et al, 2008). A wheat flour slurry was introduced into the system with or without pClc at enzyme:gliadin ratios of 1:250 and 1:50. GIP levels were quantified at various digestive stages via G12 ELISA following alcoholic extraction (see section “Enzyme-linked immunosorbent assay quantification of gluten immunogenic peptides”). As expected, no significant GIP degradation occurred during the oral phase, as pClc remains inactive in the simulated salivary fluid at pH 7 (Fig. 2J, left). Upon activation in the simulated gastric fluid (pH 1.8–2.0), Clc reduced GIP levels by up to 99% within the gastric compartment (Fig. 2J, centre), and these reductions were maintained in the downstream intestinal compartment (Fig. 2J, right). These findings demonstrate that Clc effectively degrades GIPs within a complex food matrix under simulated human digestive conditions corresponding to enzyme:flour ratios of 1:10,000 and 1:2000, respectively. Its robust activity in the gastric environment highlights its strong potential to prevent GIP-mediated immune activation in CD. As digestion progresses to the neutral pH of the intestinal compartment, Clc becomes inactive, consistent with its pH-dependent activity profile (section “In vitro peptidolytic activity and substrate specificity”).

### Immune response to the 33-mer and its celiacase cleavage products ex vivo

**Figure 3:**
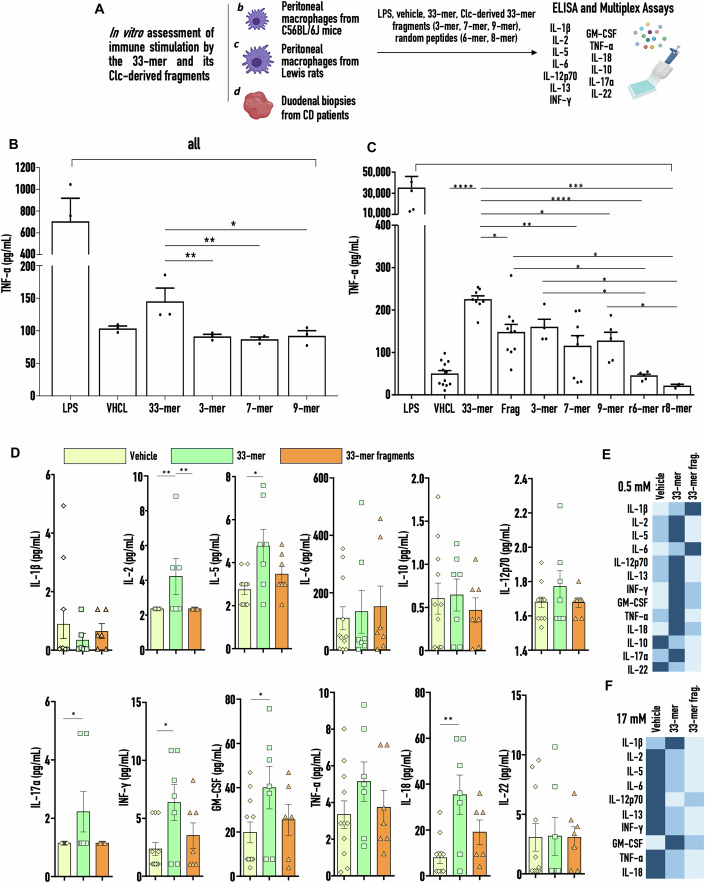
Immune response of mouse and rat macrophages and duodenal biopsies from coeliac-disease patients upon stimulation with the 33-mer and its fragments. (**A**) Experimental design for *in cell* assessment of inflammatory cytokine secretion (pg/mL) by peritoneal mouse (*n* = 10) and rat (*n* = 24) macrophages or duodenal biopsies from CD patients (*n* = 26 biopsies, 4/5 biopsies/patient) following stimulation with the 33-mer, Clc cleavage fragments (3-mer, 7-mer, 9-mer and combined), or random (r) 6-mer (T A T R G G) and 8-mer (A T L A K V S H) peptides. Only the most significant cytokines are reported. (**B**,** C**) TNF-α cytokine levels after stimulation of mouse (**B**) or rat (**C**) macrophages with stimuli at 4.25 mM. Statistical significances for mouse macrophages: LPS (*p* = 0.05 vs. Vhcl, *p* = 0.01 vs. 33-mer, *p* = 0.005 vs. 3.mer, *p* = 0.004 vs. 7-mer and *p* = 0.05 vs. 9-mer), 33-mer (*p* = 0.005 vs. 3-mer, *p* = 0.0019 vs. 7-mer, *p* = 0.047 vs. 9-mer) for rat macrophages: LPS (*p* = 4.77E-11 vs. Vhcl, *p* = 9.60E-10 vs. 33-mer, *p* = 1.21E-10 vs. Frag, *p* = 2.48E-09 vs. 3-mer, *p* = 8.65E-09 vs. 7-mer, *p* = 2.50E-09 vs. 9-mer, *p* = 8.75E-09 vs. r6-mer, *p* = 3.81E-07 vs. r8-mer), Vhcl (*p* = 5.38E-06 vs. 33-mer), 33-mer (*p* = 0.049 vs. Frag, *p* = 0.007 vs. 7-mer, *p* = 0.048 vs. 9-mer, *p* = 0.00009 vs. r6-mer, *p* = 0.0003 vs. r8-mer), Frag (*p* = 0.016 vs. r6-mer, *p* = 0.011 vs. r8-mer), 3-mer (*p* = 0.032 vs. r6-mer, *p* = 0.016 vs. r8-mer) and 9-mer (*p* = 0.037 vs. r8-mer). (**D**) Levels (pg/mL) of cytokines IL-1β, IL-2 (33-mer: *p* = 0.005 vs. Vhcl, *p* = 0.009 vs. Frag), IL-5 (33-mer: *p* = 0.011 vs. Vhcl), IL-6, IL-10, IL-12p70, IL-17α (33-mer: *p* = 0.049 vs. Vhcl), IFN-γ (33-mer: *p* = 0.028 vs. Vhcl), GM-CSF (33-mer: *p* = 0.048 vs. Vhcl), TNF-α, IL-18 (33-mer: *p* = 0.005 vs. Vhcl), and IL-22 in supernatants from duodenal biopsies of CD patients incubated with stimuli at 0.5 mM by treatment group (Vhcl, 33-mer, and 33-mer fragments). (**E**,** F**) Heatmaps of cytokine levels in biopsy supernatants by treatment group at 17 and 0.5 mM. Colour scale denotes cytokine concentration values, with intensity corresponding to magnitude (dark blue = high, light blue = low). LPS lipopolysaccharide, TNF tumour necrosis factor, IL interleukin, IFN interferon, GM-CSF granulocyte-macrophage colony-stimulating factor. Results are expressed as mean ± SEM. Statistical significance: *p ≤ 0.05, **p ≤ 0.01, ***p ≤ 0.001 and ****p ≤ 0.0001. Data were analysed using the Kruskal–Wallis test followed by Dunn’s multiple-comparisons test with Bonferroni correction. Source data are available online for this figure.

**Figure EV2:**
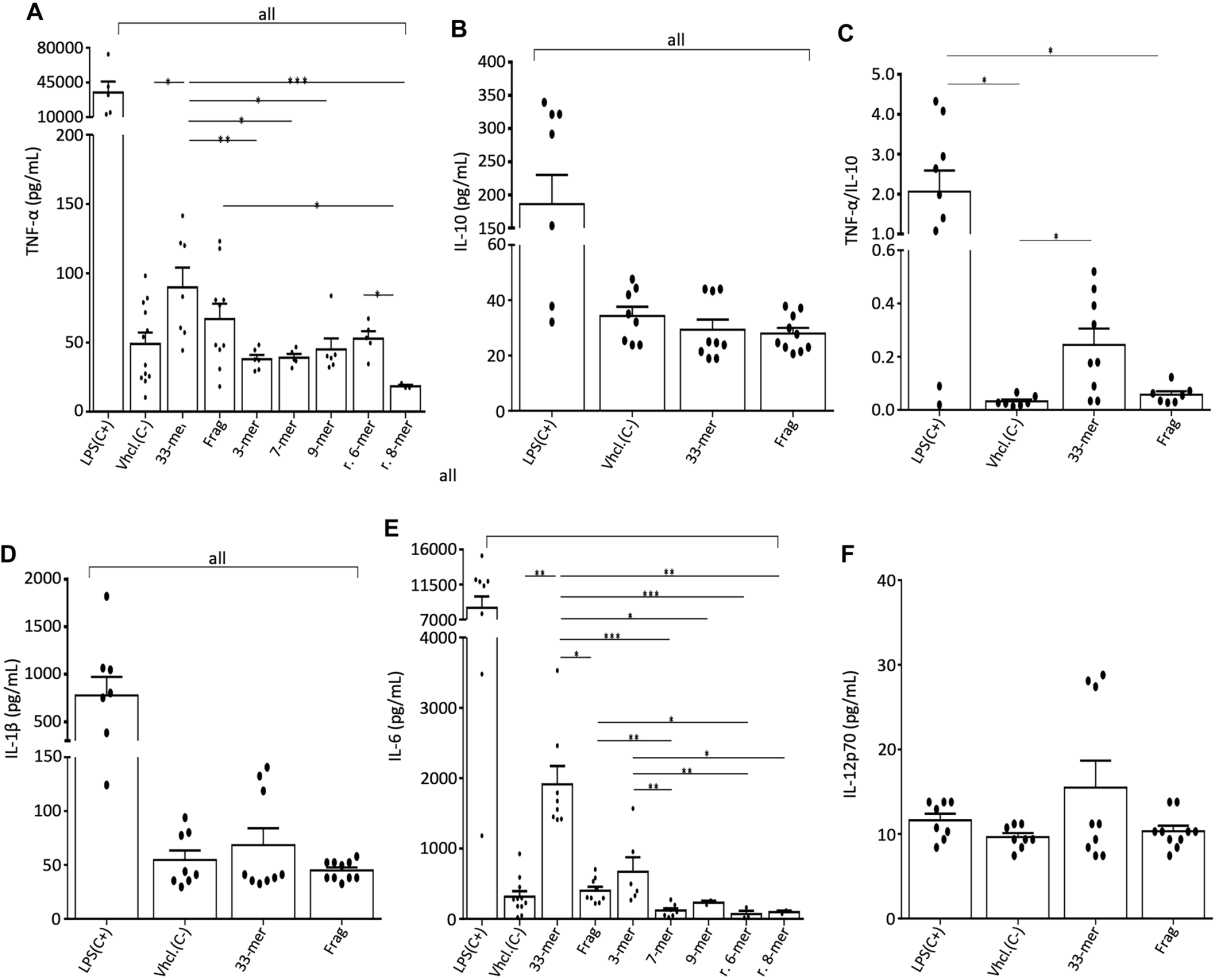
Immune response of rat macrophages upon stimulation with the 33-mer and its fragments. (**A**–**F**) Assessment of inflammatory cytokine secretion by peritoneal rat macrophages (*n* = 24) following stimulation with the 33-mer, its Clc cleavage fragments (3-mer, 7-mer, 9-mer and combined), or random (r) 6-mer (T A T R G G) and 8-mer (A T L A K V S H) peptides. Only the most significant cytokines are reported. Cytokine levels of TNF-α (**A**), IL-10 (**B**), TNF-α/IL-10 ratio (**C**), IL-1β (**D**), IL-6 (**E**), IL-12p70 (**F**); all stimuli at 4.25 mM, except (**A**) at 0.25 mM.

To evaluate immunogenic differences between the 33-mer and its Clc-derived 9-, 7- and 3-mer fragments (LQLQPFPQP, QLPYPQP and QPF; see section “In vitro peptidolytic activity and substrate specificity”; Appendix Fig. S1B), peritoneal macrophages from healthy C56BL/6J mice and Lewis rats were exposed to each peptide (Fig. 3A). Vehicle and two random-sequence peptides (6-mer and 8-mer) served as negative controls, while bacterial lipopolysaccharide (0.1 µg/mL) was included as a positive control. Cytokine levels were quantified in mouse (Fig. 3B) and in rat (Figs. 3C and EV2A–F) supernatants via ELISA.

Stimulation with the 33-mer at 4.25 mM significantly increased tumour necrosis factor (TNF)-α compared with individual fragments. Similarly, IL-6 levels remained significantly elevated in rat supernatants in response to the 33-mer relative to any fragment, either alone or in combination (Fig. EV2E). In rats, no significant differences were observed among vehicle, the 33-mer, and fragments for IL-10, IL-1β and IL-12p70 (Fig. EV2B,D,F). However, the 33-mer increased the TNF-α/IL-10 ratio (Th1-pro-inflammatory/Th2-anti-inflammatory) (Fig. EV2C), suggesting a Th1-skewed immune profile. This pattern is characteristic of autoimmune conditions such as CD, which are marked by increased production of IFN-γ, TNF-α, and IL-2 (Bellanti et al, 2003). At a lower concentration (0.25 mM), rat macrophage stimulation with the 33-mer also significantly increased TNF-α relative to individual fragments, although compared with their combined effect (Fig. EV2A). In addition, all peptides elicited lower responses than LPS, and only LPS and the 33-mer induced cytokine levels significantly above vehicle. Random peptides produced responses comparable to vehicle, indicating that the reduced activity of Clc-derived fragments was not attributable to peptide length.

Parallel experiments with duodenal biopsies from CD patients (Fig. 3A) treated with 0.5 mM peptide concentrations revealed significantly elevated levels of pro-inflammatory cytokines IL-2, IL-17α, IFN-γ, IL-18, granulocyte-macrophage colony-stimulating factor (GM-CSF) and the eosinophilotropic cytokine IL-5 in response to the 33-mer compared with vehicle (Fig. 3D,E). By contrast, the fragments did not significantly differ from vehicle—except for IL-2—indicating a markedly reduced immunogenic profile. At 17 mM (Fig. 3F), only the 33-mer induced IL-1β and GM-CSF, further underscoring its heightened immunostimulatory potential relative to its cleavage products.

Collectively, these results demonstrate that the intact 33-mer provokes a substantially stronger pro-inflammatory response than its Clc-derived cleavage fragments, both in healthy-rodent macrophages and in patient duodenal biopsies.

### Gluten degradation by celiacase in C57BL/6 J mice

**Figure 4:**
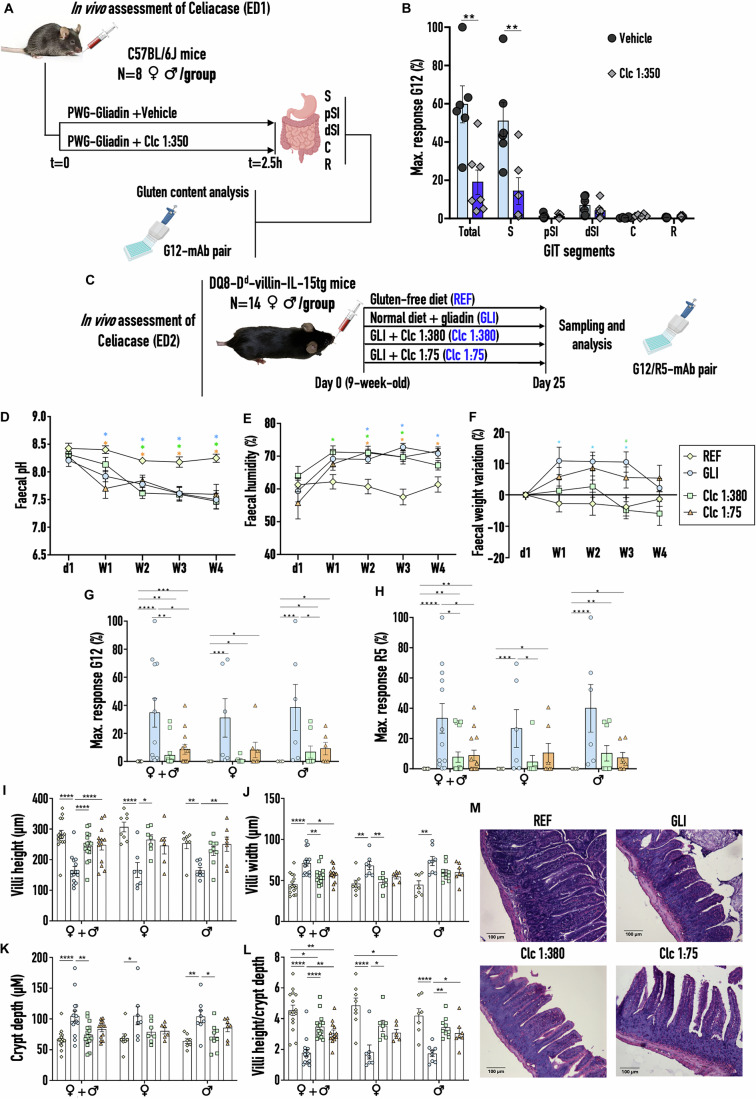
Celiacase-mediated gliadin degradation in mouse models. (**A**) Assessment of gliadin degradation in standard C57BL/6J mice from the Experimental Design 1 protocol (ED1; *n* = 8 per group, 4 females and 4 males) following administration of PWG-gliadin with vehicle or celiacase (Clc; 1:350 enzyme:gliadin ratio). Samples were collected 2.5 h post-feeding from the stomach (S), proximal small intestine (pSI), distal small intestine (dSI), caecum (C), and rectum (R). (**B**) ELISA quantification of gliadin-derived GIP content in each GIT segment (S: *p* = 0.008) and total (*p* = 0.002), using the G12 mAb after ED1. (**C**) Physiological, immunological, and histological evaluation of transgenic DQ8-D^d^-villin-IL-15tg mice following the 25-day Experimental Design 2 protocol (ED2; *n* = 14 per group; 7 females and 7 males). Mice were assigned to the REF, GLI, Clc 1:380 or Clc 1:75 groups (see sections “Gluten degradation by celiacase in transgenic DQ8-D^d^-villin-IL-15tg mice” and “Experimental design 2 (ED2)”). (**D**) Faecal pH over time at day 1 (d1) and weeks 1 (W1) (GLI: *p* = 0.044 vs. REF /Clc 1:75: *p* = 0.001 vs. REF), W2 (GLI: *p* = 0.007 vs. REF/Clc 1:380: *p* = 0,00008 vs. REF/Clc 1:75: *p* = 0.035 vs. REF), W3 (GLI: *p* = 0.004 vs. REF/Clc 1:380: *p* = 0.002 vs. REF/Clc 1:75: *p* = 0.005 vs. REF), and W4 (GLI: *p* = 0.001 vs. REF/Clc 1:380: *p* = 0.001 vs. REF/Clc 1:75: *p* = 0.007 vs. REF) (W1–W4) of ED2 per group. (**E**) Faecal humidity (%) over time at d1 and W1 (Clc 1:75: *p* = 0.011 vs. REF), W2 (GLI: *p* = 0.005 vs. REF/Clc 1:380: *p* = 0,0002 vs. REF/Clc 1:75: *p* = 0.001 vs. REF), W3 (GLI: *p* = 1.21E-06 vs. REF/Clc 1:380: *p* = 0.00006 vs. REF/Clc 1:75: *p* = 0.0002 vs. REF), and W4 (GLI: *p* = 0.002 vs. REF/Clc 1:75: *p* = 0.001 vs. REF) of ED2 per group. (**F**) Faecal weight variation (%) over time at d1 and W1 (GLI: *p* = 0.003 vs. REF), W2 (GLI: *p* = 0.048 vs. REF), W3 (GLI: *p* = 0.008 vs. REF/Clc 1:380: *p* = 0.01 vs. GLI) and W4 of ED2 per group. Symbol colours (D–F) indicate significance: * *p* ≤ 0.05 vs. REF; ^#^ *p* ≤ 0.05 vs. GLI. (**G**,** H**), ELISA of combined S, pSI, and dSI samples using the G12 mAb pair for all animals (GLI: *p* = 3.98E-08 vs. REF, *p* = 0.007 vs. Clc 1:380 and *p* = 0.046 vs. Clc 1:75/Clc 1:380: *p* = 0.003 vs. REF/Clc 1:75: *p* = 0.0003 vs. REF), only F (GLI: *p* = 0.0002 vs. REF/Clc 1:380: *p* = 0.049 vs. REF/Clc 1:75: *p* = 0.01 vs. REF) or only M (GLI: *p* = 0.0001 vs. REF and *p* = 0.045 vs. Clc 1:380/Clc 1:380: *p* = 0.028 vs. REF/Clc 1:75: *p* = 0.012 vs. REF) Same for the R5 mAb pair for all animals (GLI: *p* = 1E-07 vs. REF, *p* = 0.014 vs. Clc 1:380 and *p* = 0.001 vs. Clc 1:75/Clc 1:380: *p* = 0.002 vs. REF/Clc 1:75: *p* = 0.001 vs. REF), only F (GLI: *p* = 0.0004 vs. REF and *p* = 0.043 vs. Clc 1:380/Clc 1:75: *p* = 0.022 vs. REF) or only M (GLI: *p* = 0.0001 vs. REF/Clc 1:380: *p* = 0.006 vs. REF/Clc 1:75: *p* = 0.018 vs. REF). (**I**) Histological analysis of villi height of dSI per group for all animals (GLI: *p* = 3.4E-07 vs. REF, *p* = 0.0004 vs. Clc 1:380 and *p* = 0.0004 vs. Clc 1:75), only F (GLI: *p* = 0.0003 vs. REF and *p* = 0.014 vs. Clc 1:380) or only M (GLI: *p* = 0.006 vs. REF and *p* = 0.008 vs. Clc 1:75). (**J**) Histological analysis of villi width of dSI per group for all animals (GLI: *p* = 1.45E-06 vs. REF, *p* = 0.002 vs. Clc 1:380 and *p* = 0.022 vs. Clc 1:75), only F (GLI: *p* = 0.003 vs. REF and *p* = 0.009 vs. Clc 1:380) or only M (GLI: *p* = 0.001 vs. REF). (**K**) Histological analysis of crypt depth of dSI per group for all animals (GLI: *p* = 0.0002 vs. REF and *p* = 0.003 vs. Clc 1:380), only F (GLI: *p* = 0.049 vs. REF) or only M (GLI: *p* = 0.006 vs. REF and *p* = 0.017 vs. Clc 1:380). (**L**) Histological analysis of the villi height/crypt depth ratio of dSI per group for all animals (GLI: *p* = 1.76E-09 vs. REF, *p* = 0,00007 vs. Clc 1:380 and *p* = 0.006 vs. Clc 1:75/Clc 1:380: *p* = 0.022 vs. REF/Clc 1:75: *p* = 0.001 vs. REF), only F (GLI: *p* = 0.0001 vs. REF and *p* = 0.048 vs. Clc 1:380/Clc 1:75: *p* = 0.038 vs. REF) or only M (GLI: *p* = 0.00003 vs. REF, *p* = 0.002 vs. Clc 1:380 and *p* = 0.031 vs. Clc 1:75). (**M**) Representative haematoxylin and eosin-stained dSI sections from each group at the end of ED2. Results are expressed as mean ± SEM. Statistical significance: *p ≤ 0.05, **p ≤ 0.01, ***p ≤ 0.001 and ****p ≤ 0.0001. Data showing a normal distribution and homogeneity of variance (**B**, **D**, **F**, **I**–**L**) were analysed using one- or two-way *ANOVA* followed by Bonferroni post hoc tests. Non-parametric data (**E**, **G**) were analysed using the Kruskal–Wallis test with Dunn’s post hoc test and Bonferroni correction. Source data are available online for this figure.

We next assessed whether Clc could withstand the harsh GIT environment (pH 1–7.5; 37 °C) while maintaining gluten-degrading activity in vivo. Healthy inbred C57BL/6J mice received either vehicle or Clc, together with PWG-gliadin at a 1:350 ratio (*n* = 8 per group; 4 males and females; Fig. 4A), following experimental design 1 (ED1; see Experimental design 1 (ED1)). The gliadin administered would translate to a human-equivalent dose of 13–16 g of gliadin (16–20 g of gluten) per kg body weight. After 2.5 h, contents from the stomach, proximal and distal small intestine, caecum, and rectum were collected, and GIPs were quantified in each compartment via sandwich ELISA using the G12 mAb pair (Fig. 4B). As most of the bolus remained in the stomach at this time point, this compartment provided the only reliable material for quantification. In Clc-treated mice, both total and gastric GIP levels were reduced by ≈72% compared with controls, demonstrating that Clc retains enzymatic activity and effectively degrades gluten under in vivo conditions following acute gliadin ingestion.

### Gluten degradation by celiacase in transgenic DQ8-D^d^-villin-IL-15tg mice

Building on the in vitro, ex vivo, and acute in vivo findings, we performed an intervention in the DQ8-D^d^-villin-IL-15tg CD model maintained on a GFD (experimental design 2, ED2). Mice were allocated to one of four groups (see section “Experimental design 2 (ED2)”): continued GFD (REF), gluten-containing diet supplemented with additional gliadin bolus (GLI), or GLI combined with Clc at very low (Clc 1:380) or low (Clc 1:75) doses (*n* = 14 per group; 7 males and 7 females; Fig. 4C). Consistent differences between REF and GLI groups across all measured variables confirmed successful disease induction (Figs. 4D–M; and EV3).

**Figure EV3:**
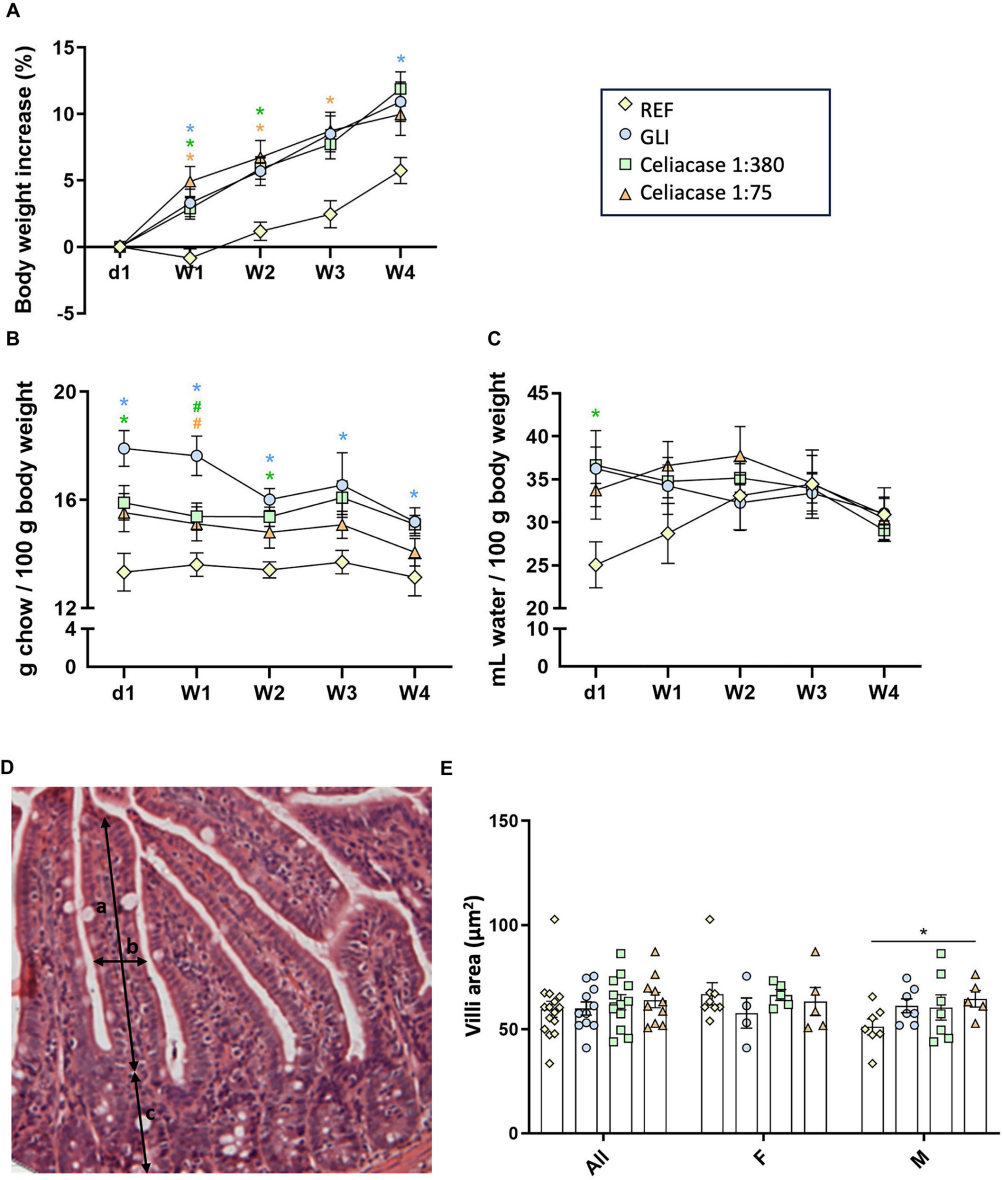
Body weight, food and water intake of DQ8-D^d^-villin-IL-15tg mice during the ED2 intervention, and post-intervention histological analysis. (**A**–**C**) Percentage increase in body weight, chow consumption, and water intake at day 1 (d1) and weeks 1–4 (W1–W4) within the 25-day experiment for the REF, GLI; Clc 1:380, and Clc 1:75 groups. Symbol colours indicate significant differences: *p ≤ 0.05 vs. REF; ^#^*p* ≤ 0.05 vs. GLI. (**D**) Schematic representation of histological measurements: a = villi length, b = villi width, c = crypt depth, a × b = villi area, b/c = villi length-to-crypt depth ratio. (**E**) Villi area of the distal small intestine split into the REF, GLI, Clc 1:380 and Clc 1:75 groups. In all cases, *n* = 7 per group and sex, results are expressed as mean ± SEM for females (F), males (M), and combined (all), * stands for *p* ≤ 0.05 vs. REF. Statistical analyses are as described in section “Statistical and data analysis”.

Body weight increased in all groups during the 25-day ED2, but was significantly higher in gluten-fed mice (Fig. EV3A). GLI mice also exhibited increased chow intake, a trend not observed in Clc-treated groups (Fig. EV3B), while water consumption remained unchanged (Fig. EV3C). Faecal pH and humidity, indicative of diarrhoea, were significantly altered in all gliadin-fed groups, with reduced pH and increased humidity (Fig. 4D,E). By week 4, Clc at 1:380 reversed the elevated faecal humidity observed in the GLI and Clc 1:75 groups. Both Clc doses also prevented the increase in faecal weight seen in GLI mice, aligning values more closely with REF (Fig. 4F).

On the final day of ED2, morphometric and weight assessments included organ weights, naso-anal length, total body length, body-mass index (BMI) and Lee Index (Appendix Table S2). No significant differences were observed among groups except for reduced stomach weight in GLI mice and increased caecum weight in GLI and both Clc-treated groups compared to REF. Haematological analysis (Appendix Table S3) revealed significantly lower red blood cell count, haemoglobin, haematocrit, and platelet count in the Clc 1:380 group compared with REF and GLI. Platelet large cell count was also reduced in both Clc-treated groups versus REF.

Stomach and small intestine contents were collected 1.5 h after gliadin administration and analysed by G12 and R5 ELISA, as described in section “Gluten degradation by celiacase in C57BL/6J mice”. As expected, no GIP signal was detected in the REF group, confirming assay specificity (Fig. 4G,H). In contrast, the GLI group exhibited strong GIP signals, whereas Clc treatment significantly reduced GIP levels—as revealed by either mAb pair—by up to 88% across all animals, irrespective of sex, which is consistent with the findings in C57BL/6J mice.

Histological analysis of the distal small intestine, where villous atrophy is most pronounced in this model (Abadie et al, 2022; Abadie et al, 2020), was performed (Figs. 4I–M and EV3D,E), and representative haematoxylin-and-eosin-stained sections from each group are depicted in Figs. 4M and EV3D. Significant differences between REF and GLI groups were observed for villus height, villus width, crypt depth, and villus height/crypt depth ratio (Fig. 4I–L), but not for villus area (Fig. EV3E), confirming successful disease induction in the model. Clc treatment at both doses significantly prevented the reduction in villus height and villus height/crypt depth ratio, as well as the increase in villus width and crypt depth (Fig. 4L).

In summary, the transgenic CD model was successfully established, and Clc administration markedly reduced GIP levels in the GIT—by up to 88%—while attenuating hallmark histological features of CD, including villous atrophy.

### Celiacase modulates systemic and intestinal immunoglobulin profiles in gluten-fed DQ8-D^d^-villin-IL-15tg mice

**Figure 5:**
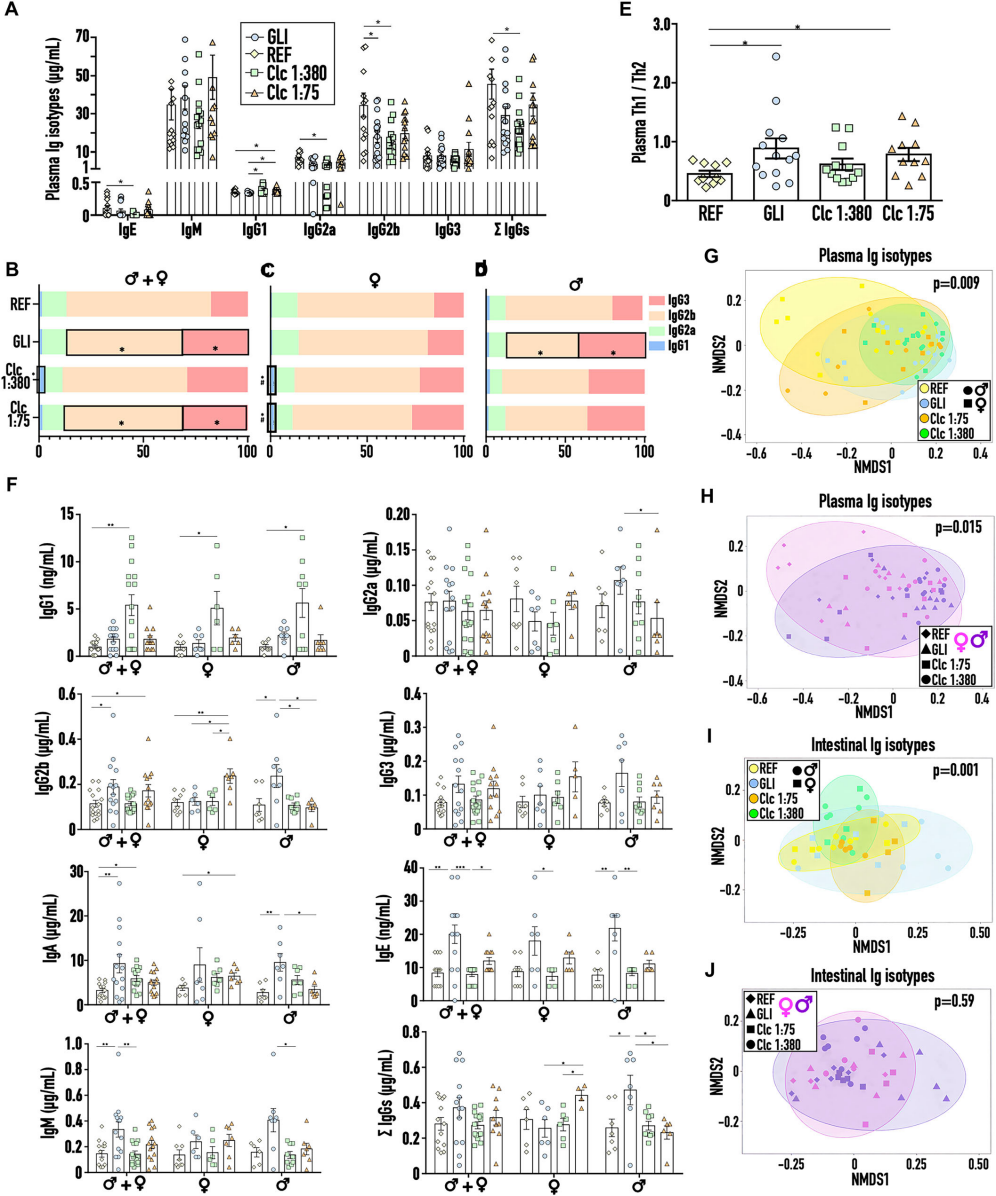
Systemic and intestinal immunoglobulin responses to celiacase treatment in gluten-fed DQ8-D^d^-villin-IL-15tg mice. (**A**) Plasma levels of IgE (Clc 1:380 *p* = 0.01 vs. REF), IgM, and IgG1 (GLI: *p* = 0.022 vs. Clc 1:380 and *p* = 0.006 vs Clc 1:75/Clc 1:75: *p* = 0.018 vs. REF), IgG2a (Clc 1:380: *p* = 0.002 vs. REF), IgG2b (GLI: *p* = 0.037 vs. REF/Clc 1:380: *p* = 0.014 vs. REF), IgG3 and total IgGs (Clc 1:380: *p* = 0.018 vs. REF) across the REF, GLI, Clc 1:380, and Clc 1:75 groups, measured at the end of ED2. (**B**–**D**) Relative proportions of IgG subclasses (IgG1, IgG2a, IgG2b, and IgG3) to total IgG, shown for all animals (GLI: *p*_*IgG2b*_ = 0.017 vs. REF and *p*_*IgG3*_ = 0.042 vs. REF/Clc 1:380 *p*_*IgG1*_ = 0.007 vs. REF/Clc 1:75 *p*_*IgG2b*_ = 0.021 vs. REF, *p*_*IgG3*_ = 0.043 vs. REF), females (GLI: *p*_*Ig1*_ = 0.034 vs. Clc 1:380 and *p*_*IgG1*_ = 0.045 vs. Clc 1:75/Clc 1:380 *p*_*IgG1*_ = 0.026 vs. REF/Clc 1:75 *p*_*IgG2b*_ = 0.035 vs. REF, *p*_*IgG3*_ = 0.043 vs. REF) and males (GLI: *p*_*IgG2b*_ = 0.021 vs. REF and *p*_*IgG3*_ = 0.049 vs. REF). Statistical significance: *p ≤ 0.05 vs. REF; #*p* ≤ 0.05 vs. GLI. (**E**) Th1/Th2 ratio, calculated as [IgG2a + IgG3]/[IgG1 + IgG2b] (GLI: *p* = 0.02 vs. REF/Clc 1:75: *p* = 0.022 vs. REF). (**F**) Intestinal Ig isotypes levels of IgG1 (All: Clc 1:380: *p* = 0.0001 vs. REF/F: Clc 1:380: *p* = 0.018 vs. REF/M: Clc 1:380: *p* = 0.012 vs. REF), IgG2a (M: GLI: *p* = 0.035 vs. Clc 1:380), IgG2b (All: GLI: *p* = 0.031 vs. REF and Clc 1:75: *p* = 0.049 vs. REF/F: Clc 1:75: *p* = 0.004 vs. REF, *p* = 0.012 vs. GLI and *p* = 0.013 vs. Clc 1:380/M: GLI: *p* = 0.02 vs. REF, *p* = 0.044 vs. Clc 1:380 and *p* = 0.04 vs. Clc 1:75), IgG3, IgA (All: GLI: *p* = 0.004 vs. REF and Clc 1:380: *p* = 0.019 vs. REF/F: Clc 1:75: *p* = 0.044 vs. REF /M: GLI: *p* = 0.002 vs. REF and *p* = 0.023 vs. Clc 1:75), IgE (All: GLI: *p* = 0.001 vs. REF and *p* = 0.0003 vs. Clc 1:380/Clc 1:380: *p* = 0.042 vs. Clc 1:75/F: GLI: *p* = 0.031 vs. Clc 1:380/M: GLI: *p* = 0.003 vs. REF and *p* = 0.003 vs. Clc 1:380), IgM (All: GLI: *p* = 0.004 vs. REF and *p* = 0.005 vs. Clc 1:380/M: GLI: *p* = 0.013 vs. Clc 1:380), and total IgGs (F: GLI: *p* = 0.018 vs. Clc 1:75 and Clc 1:380 *p* = 0.025 vs. Clc 1:75/M: GLI: *p* = 0.033 vs. REF, *p* = 0.033 vs. Clc 1:380 and *p* = 0.033 vs. Clc 1:75), by group. (**G**–**J**) NMDS analysis of plasma and intestinal Ig profiles, grouped by group (**G**, **I**) and by sex (**H**, **J**), with corresponding *p*-values. Group differences were assessed using the analysis of similarities (*ANOSIM*) test. Results are expressed as mean ± SEM (**A**,** E**,** F**), *n* = 14/group, equally divided between females and males (*n* = 7/group and sex). Statistical significance: *p ≤ 0.05, **p ≤ 0.01, ***p ≤ 0.001 and ****p ≤ 0.0001. Data showing a normal distribution and homogeneity of variance (**B**–**D**) were analysed using one- or two-way *ANOVA* followed by Bonferroni post hoc tests. Non-parametric data (**A**, **E**, **F**) were analysed using the Kruskal–Wallis test with Dunn’s post hoc test and Bonferroni correction. Source data are available online for this figure.

Plasma concentrations of Ig isotypes and subclasses (IgA, IgE, IgM, IgG1, IgG2a, IgG2b and IgG3) were analysed across the four experimental groups (REF, GLI, Clc 1:380 and Clc 1:75) and by sex following ED2 (Fig. 5A). Notably, Clc-treated mice exhibited a higher proportion of IgG1 compared with both REF and GLI groups. Further analysis by group and sex (Fig. 5B–D) revealed that both Clc treatments counteracted the GLI-induced decrease in IgG2b, and that Clc 1:380 prevented the IgG3 increase observed in males and in the combined group. In females, both Clc doses significantly increased IgG1 proportions relative to REF and GLI.

To evaluate the Th1/Th2 balance, the ratio [IgG2a + IgG3]/[IgG1 + IgG2b] was calculated, with higher values indicating a Th1-skewed, pro-inflammatory profile typically associated with autoimmune conditions such as CD. The GLI group displayed a significantly elevated Th1/Th2 ratio compared with REF, an effect reversed by Clc 1:380, suggesting mitigation of the pro-inflammatory state (Fig. 5E). At the intestinal level, the concentration of IgG2b, IgA, IgE and IgM isotypes—regardless of sex—and IgGs—only in males— were significantly higher in GLI than in REF animals. This increase was prevented for IgE and IgM by either one or both doses of Clc in all animals, while for IgG2b, IgA and all IgGs this effect was exclusively observed in males (Fig. 5F). To explore clustering patterns in Ig profiles by treatment or sex, non-metric multidimensional scaling (NMDS) analyses (see section “Statistical and data analysis”) were performed separately for plasma (Fig. 5G,H) and intestinal samples (Fig. 5I,J), using Gower’s distance. Stress values (0.13 for plasma, 0.09 for intestine) indicated a reliable two-dimensional representation of the data. In plasma, Ig isotype composition differed significantly by treatment (*p* = 0.009) and sex (*p* = 0.015), driven mainly by a strong difference between REF and Clc 1:380 (*p* = 0.001) (Fig. 5G,H). Similarly, intestinal Ig profiles clustered significantly by treatment (*p* = 0.001) but not by sex (*p* = 0.59), with major differences between REF–Clc 1:380 (*p* = 0.003) and GLI–Clc 1:380 (*p* = 0.003) (Fig. 5I,J). To further assess the influence of sex, Ig data were analysed separately (Appendix Fig. S5A,B). No significant clustering was detected in females, whereas in males, REF–Clc 1:380 remained significant (*p* = 0.001). When focusing on IgG subclasses, NMDS revealed significant clustering by treatment and sex (Appendix Fig. S5C,D; stress = 0.10). Separate analyses confirmed significant treatment-driven clustering in both females (stress = 0.09) and males (stress = 0.08) (Appendix Fig. S5E,F). Pairwise comparisons indicated significant differences between REF and Clc 1:380 in all animals (*p* = 0.019), females (*p* = 0.027), and males (*p* = 0.037), as well as between GLI and Clc 1:75 (*p* = 0.045, *p* = 0.040, and *p* = 0.040, respectively).

Together, these findings demonstrate that Clc treatment not only modulates systemic and mucosal Ig isotype/subclass distributions but also rebalances the Th1/Th2 immune profile, with NMDS confirming distinct, treatment-driven clustering patterns.

### Celiacase affects plasma and intestine cytokine profiles and disease-specific auto-antibodies in gluten-fed DQ8-D^d^-villin-IL-15tg mice

**Figure 6:**
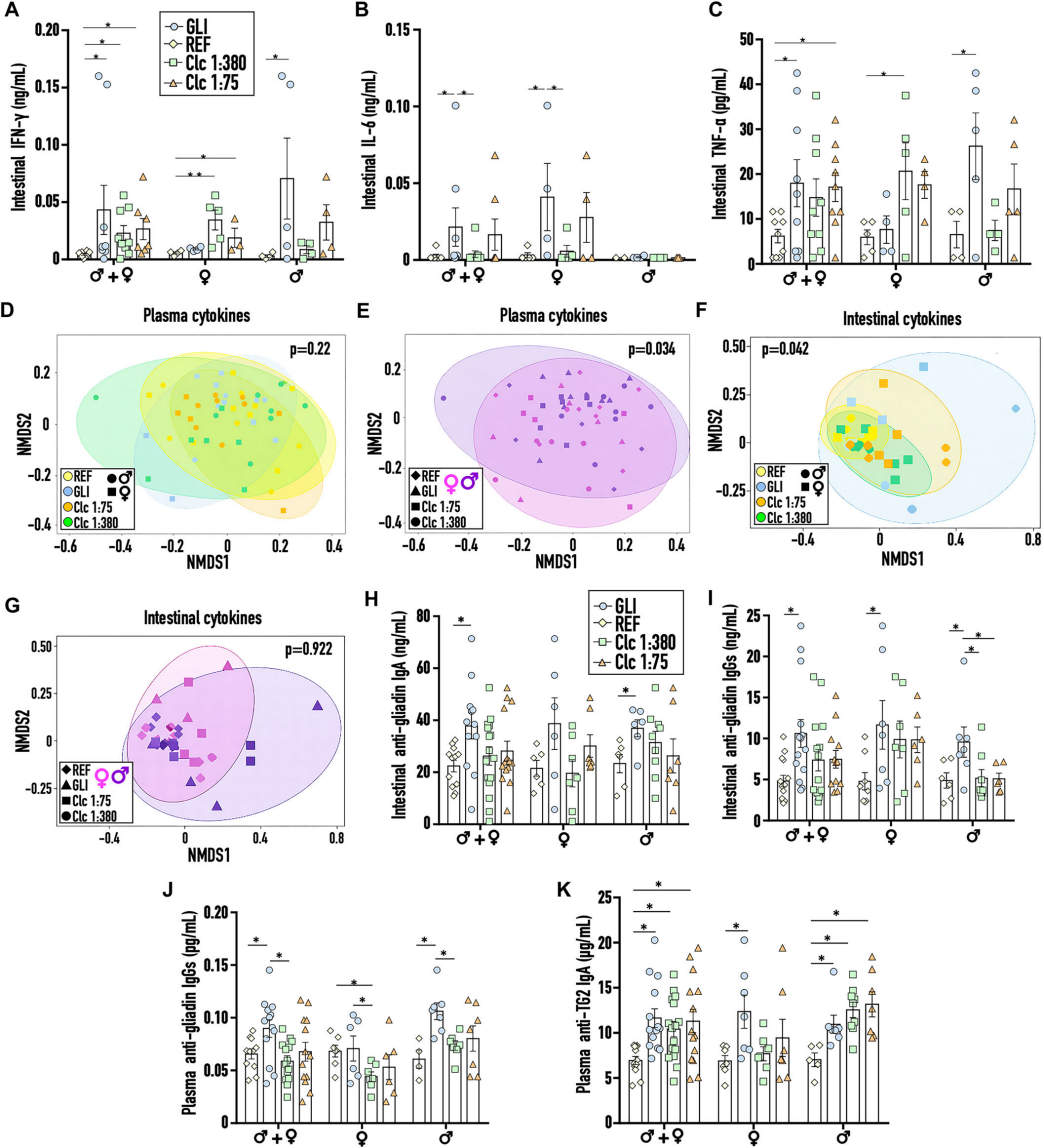
Cytokine responses to celiacase treatment in gluten-fed DQ8-D^d^-villin-IL-15tg mice. (**A**–**C**) Intestinal levels of pro-inflammatory cytokines IFN-γ (All: REF: *p* = 0.0029 vs. GLI, *p* = 0.015 vs. Clc 1:380 and *p* = 0.018 vs. Clc 1:75/F: REF: *p* = 0.003 vs. Clc 1:380 and *p* = 0.039 vs. Clc 1:75/M: REF: *p* = 0.043 vs. GLI), IL-6 (All: GLI: *p* = 0.035 vs. REF and *p* = 0.046 vs. Clc 1:380/F: GLI: *p* = 0.017 vs. REF and *p* = 0.034 vs. Clc 1:380), and TNF-α (All: REF: *p* = 0.049 vs. GLI and *p* = 0.025 vs. Clc 1:75/F: REF: *p* = 0.037 vs. Clc 1:380/M: REF: *p* = 0.045 vs. GLI), measured at the end of ED2 across the REF, GLI, Clc 1:380 and Clc 1:75 groups. Data are shown for all animals, females, and males. (**D**–**G**) NMDS analysis of plasma and intestine cytokine profiles by group (**D**, **F**) and by sex (**E**, **G**), with corresponding *p* values. Group differences were assessed using the analysis of similarities (*ANOSIM*) test. (**H**–**K**) Levels of intestinal anti-gliadin IgA (All: REF: *p* = 0.043 vs. GLI/M: REF: *p* = 0.035 vs. GLI), intestinal anti-gliadin IgG (All: REF: *p* = 0.002 vs. GLI/F: REF: *p* = 0.022 vs. GLI/M: GLI: *p* = 0.031 vs. REF, *p* = 0.021 vs. Clc 1:380 and *p* = 0.049 vs. Clc 1:75), plasma anti-gliadin IgG (All: GLI: *p* = 0.048 vs. REF and *p* = 0.007 vs. Clc 1:380/F: Clc 1:380: *p* = 0.028 vs. REF and *p* = 0.043 vs. GLI/M: GLI: *p* = 0.006 vs. REF and *p* = 0.027 vs. Clc 1:380) and plasma anti-TG2 IgA (All: REF: *p* = 0.0004 vs. GLI, *p* = 0.006 vs. Clc 1:380 and *p* = 0.011 vs. Clc 1:75/F: REF: *p* = 0.025 vs. GLI/M: REF: *p* = 0.007 vs. GLI, *p* = 0.006 vs. Clc 1:380 and *p* = 0.004 vs. Clc 1:75) auto-Abs. Results are expressed as mean ± SEM, *n* = 14/group, equally divided between females and males (*n* = 7/group and sex). Statistical signficance: *p ≤ 0.05, **p ≤ 0.01, ***p ≤ 0.001 and ****p ≤ 0.0001. For data showing a normal distribution and homogeneity of variance (**H**, **J**, **K**), one- or two-way *ANOVA* followed by Bonferroni post hoc tests was performed. Non-parametric data (**A**–**C**, **I**) were analysed using the Kruskal–Wallis test with Dunn’s post hoc test and Bonferroni correction. Source data are available online for this figure.

Intestinal concentrations of three key pro-inflammatory cytokines associated with CD were significantly elevated in GLI mice compared with REF as a result of gliadin intake, specifically IFN-γ and TNF-α in males, and IL-6 in females (Fig. 6A–C). These increments were prevented by Clc treatment. To further investigate cytokine patterns, NMDS analyses were performed on plasma and intestinal cytokine profiles (Fig. 6D–G). Plasma cytokines (stress = 0.08) clustered significantly by sex (*p* = 0.034) but not by treatment (*p* = 0.22), whereas intestinal cytokines (stress = 0.12) showed significant clustering by treatment (*p* = 0.042) but not by sex (*p* = 0.922). No overall treatment-related differences in plasma cytokine levels were observed (Appendix Fig. S6A–C). However, subgroup analyses indicated elevated IL-4, IL-5, IFN-γ and TNF-α in GLI females, and increased IL-5 and TNF-α in GLI males compared with REF; these elevations were mitigated by Clc 1:380.

Profiling of CD-specific auto-Abs revealed consistently higher levels in GLI animals compared with REF in both plasma and intestinal wash samples, irrespective of sex, isotype (IgG or IgA), or antigen specificity (anti-gliadin or anti-TG2), confirming successful disease induction (Fig. 6H–K). Importantly, both Clc treatments—particularly Clc 1:380—prevented gliadin-induced increases in auto-Abs across multiple contexts, including anti-gliadin IgA and IgG in plasma and gut wash from males and females, and anti-TG2 IgA in plasma from females.

Overall, these findings demonstrate that Clc effectively prevents intestinal inflammation and gliadin-induced increases in both pro-inflammatory cytokines and disease-relevant auto-Abs.

### Celiacase modulates mesenteric lymph-node lymphocyte phenotypes in gluten-fed DQ8-D^d^-villin-IL-15tg mice

**Figure 7:**
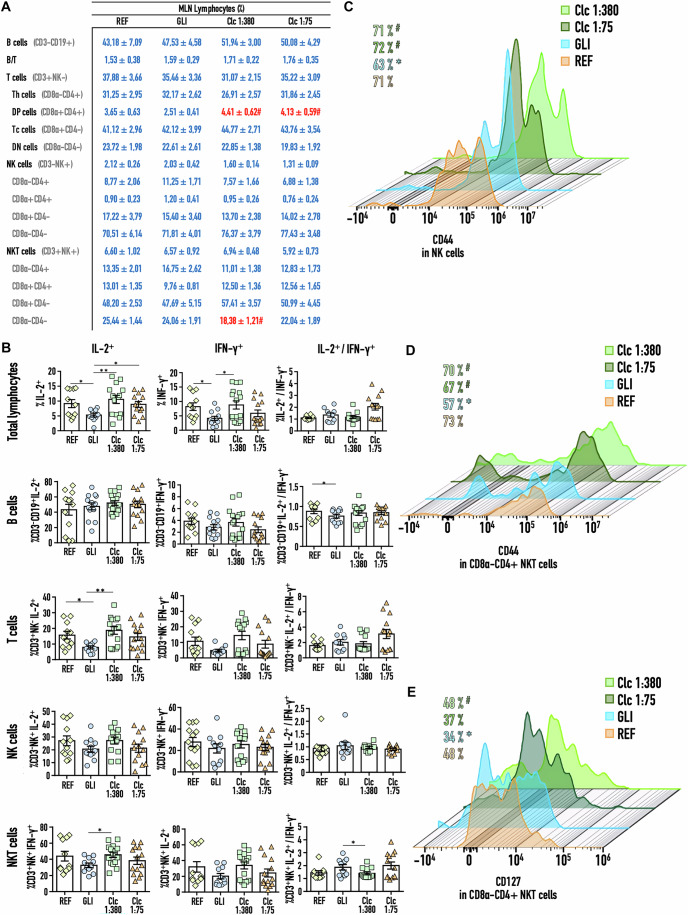
Lymphocyte phenotyping in celiacase-treated gluten-fed DQ8-D^d^-villin-IL-15tg mice. (**A**) Proportions of major MLN lymphocyte populations following completion of ED2 across the REF, GLI, Clc 1:380 and Clc1:75 groups. Analysed cells include B cells, T cells (T-helper [Th], double positive [DP], cytotoxic T [Tc], double negative [DN]), natural killer (NK) cells and natural killer T (NKT) cells. Statistically significant differences for Clc 1:75 and Clc 1:380 vs. GLI are indicated in red and labelled with ^#^ (*p* ≤ 0.05). (**B**) Proportions of IL-2^+^, IFN-γ^+^ and their ratio within (top to bottom) total lymphocytes (IL-2^+^: GLI: *p* = 0.037 vs. REF, *p* = 0.007 vs. Clc 1:380 and *p* = 0.041 vs. Clc 1:75/IFN-γ^+^: GLI: *p* = 0.023 vs. REF and *p* = 0.02 vs. Clc 1:380), along with three different types of B cells (IL-2^+^/IFN-γ^+^: REF: *p* = 0.036 vs. GLI), T cells (IL-2^+^: GLI: *p* = 0.025 vs. REF and *p* = 0.004 vs. Clc 1:380), NK cells, and NKT cells for each group. Statistical significance: *p ≤ 0.05, **p ≤ 0.01, ***p ≤ 0.001 and ****p ≤ 0.0001. (**C**–**E**) Histogram plots showing the percentage of CD44 expression in NK and CD8α^–^CD4^+^ NKT cells by group, and of CD127 in CD8α^–^CD4^+^ NKT cells. Statistically significant differences for Clc 1:75 and Clc 1:380 are indicated in bold and labelled with ^#^ (*p* ≤ 0.05) versus GLI and with *(*p* ≤ 0.05) versus REF. Results are expressed as mean ± SEM, *n* = 14/group. For data showing a normal distribution and homogeneity of variance (**A**, **C**, **D**, **E**), one- or two-way *ANOVA* followed by Bonferroni post hoc tests was performed. Non-parametric data (**B**) were analysed using the Kruskal–Wallis test with Dunn’s post hoc test and Bonferroni correction. Source data are available online for this figure.

Given the central role of mucosal lymphocytes in CD progression, we performed comprehensive profiling of major lymphocyte subsets in the mesenteric lymph nodes (MLNs) across the REF, GLI, Clc 1:380 and Clc 1:75 groups (Fig. 7A–E). B cells, T cells, natural killer (NK) cells and natural killer T (NKT) cells were quantified by flow cytometry (see section “Phenotyping of mesenteric lymph node lymphocytes”), which revealed a significant increase in CD8α^+^CD4⁺ double-positive T cells and CD8α⁻CD4⁻ double-negative NKT cells in Clc-treated groups (Fig. 7A). Functional assessment by intracellular cytokine staining showed that Clc prevented gliadin-induced reductions in IL-2 and IFN-γ expression in total lymphocytes, IL-2 in T and NKT cells, and the IL-2/IFN-γ ratio in B cells (Fig. 7B). Clc also mitigated gliadin-associated alterations in CD44 and CD127 receptor expression—key mediators of immune cell adhesion, migration, and survival—particularly in NK cells (Fig. 7C) and CD8α⁻CD4⁺ NKT cells (Fig. 7D,E). In summary, Clc significantly counteracts gliadin-driven immunophenotypic shifts in key lymphocyte populations, supporting its potential as an immunomodulatory agent.

### Caecal microbiota modulation by celiacase in gluten-fed DQ8-D^d^-villin-IL-15tg mice

**Figure 8:**
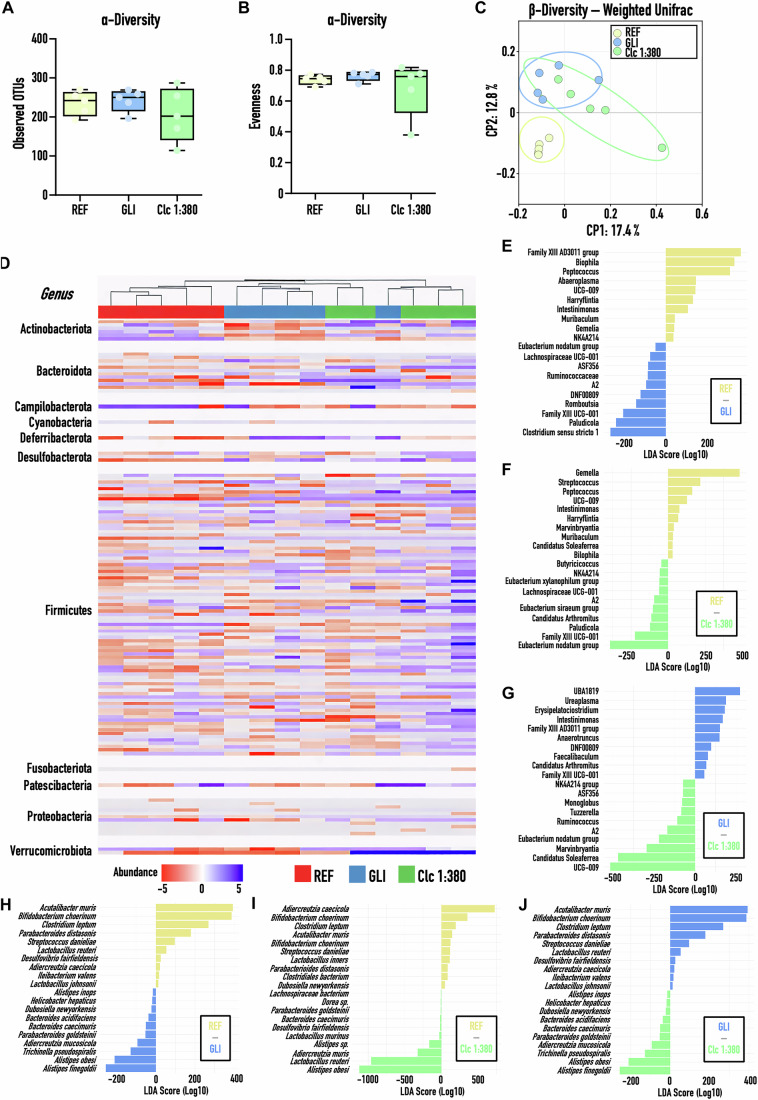
Caecal microbiota profiling in celiacase-treated gluten-fed DQ8-D^d^-villin-IL-15tg mice. Microbiome profiling by 16S rRNA sequencing for the REF, GLI, and Clc 1:380 groups, following completion of ED2 (*n* = 5/group, only females). (**A**,** B**) α-Diversity of the caecal microbiota, expressed as observed operational taxonomic units (**A**) and evenness (**B**). Box plots show the median (centre line), interquartile range (25th–75th percentiles; box), and minimum to maximum values (whiskers). (**C**) Principal component analysis of β-diversity based on weighted-unifrac distance. (**D**) Heatmap of genus-level abundance, clustered by dendogram; row labels indicate phyla, and colours represent standardised genus abundance (red = high, blue =  low). (**E**–**J**) Linear discriminant analysis plots comparing genera (**E**–**G**) and species (**H**–**J**) across the three groups. Statistical significance (*p* < 0.05*)* was assessed by permutational multivariate analysis of variance (*PERMANOVA*) and analysis of similarities (*ANOSIM*), and dispersion effects by permutational analysis of multivariate dispersions (*PERMDISP*). Source data are available online for this figure.

Comparative 16S rRNA sequencing of caecal microbiota from REF, GLI and Clc 1:380 groups revealed no significant differences in α-diversity, as measured by observed operational taxonomic units and evenness (Fig. 8A,B), indicating stable microbial richness and within-group diversity across treatments. In contrast, β-diversity analysis (Fig. 8C), which assesses between-group dissimilarity, demonstrated significant differences in community structure (*p* = 0.001), with distinct clustering between REF–GLI (*p* = 0.0135), REF–Clc 1:380 (*p* = 0.0135) and GLI–Clc 1:380 (*p* = 0.044). Principal coordinate analysis positioned Clc 1:380 samples between REF and GLI, suggesting partial prevention of gluten-induced microbiota alterations. Heatmap clustering of bacterial genera (Fig. 8D) corroborated these findings, with samples grouping by treatment.

At the phylum level, GLI mice exhibited increased abundance of Actinobacteria, Proteobacteria, and Patescibacteria compared with REF, all of which were prevented by Clc 1:380 (Appendix Fig. S7). However, Clc 1:380 did not avoid the GLI-associated decrease in Desulfobacterota and Verrucomicrobiota or the increase in Firmicutes. Linear discriminant analysis (see section “Caecal microbiota profiling”) at both genus (Appendix Fig. S8) and species (Appendix Fig. S9) levels identified distinct taxonomic signatures across groups (Fig. 8E–J). REF mice were enriched in the *Family XII AD3011* genus, *Bilophila*, and *Peptococcus*, whereas GLI mice showed strong associations with *Clostridium sensu stricto 1* and *Paludicola*. Clc 1:380 was associated with *Gemella* (vs. REF) and *Candidatus Soleaferrea* (vs. GLI), whereas REF and GLI showed closer associations with the *Eubacterium nodatum* group and *UBA 1819* and *UCG-009* groups, respectively. Analysis of genera showing significant differences between groups (Appendix Fig. S8A–C) indicated that Clc 1:380 prevented gluten-induced shifts in *Acetatifactor*, *Lachnospiraceae UCG-006*, *Turicibacter*, *Muribaculaceae*, *Odoribacter*, *Chlamydia*, *Candidatus Saccharimonas*, and *Desulfovibrio*. At the species level (Fig. 8H–J), *Acutalibacter muris* and *Bifidobacterium choerinum* were enriched in REF, whereas *Alistipes finegoldii* and *Alistipes obesi* were associated with GLI. Clc 1:380 was linked to *Lactobacillus reuteri* and *A. obesi*, and mitigated GLI-associated changes in *A. muris*, *Alistipes inops*, *Bacteroides acidifaciens*, *Adlercreutzia mucosicola*, *Chlamydia muridarum*, and *Desulfovibrio fairfieldensis* (Appendix Fig. S9). Relative abundance at various taxonomic levels across groups are shown in bar plots (Appendix Fig. S10A–F).

To assess the functional implications of these microbial shifts, we employed *PICRUSt* and the KEGG database (see Section “Caecal microbiota profiling”) to predict metabolic pathway activity of caecal microbiota across the three groups (Appendix Fig. S11A–D). Enrichment analysis revealed that REF mice exhibited higher predicted activity in pathways such as the phosphotransferase system, carbon metabolism, and the biosynthesis of cofactors and amino acids compared with both GLI and Clc 1:380. By contrast, GLI animals showed enrichment in bacterial secretion and biosynthetic pathways relative to Clc 1:380. When comparing REF–GLI and REF–Clc 1:380, six of the top ten pathways enriched in REF–GLI displayed reduced gene representation in REF–Clc 1:380, and eight of the top 30 were absent altogether, indicating a partial preventive effect of Clc. The GLI–Clc 1:380 comparison revealed fewer enriched pathways and lower gene counts, further supporting the modulatory effect of Clc on microbial function. A detailed visualisation of gene orthologues involved in the most enriched pathways for each pairwise comparison is provided in the category net plots (Appendix Fig. S12A–C).

Overall, these findings demonstrate that Clc 1:380 did not alter microbial richness but significantly modulated community structure and composition, partially maintaining a profile closer to that of REF. It prevented gliadin-induced increases in several pro-inflammatory phyla and taxa, and preserved key metabolic functions, supporting its protective role against gluten-induced dysbiosis in CD-prone transgenic mice.

### Global non-metric multidimensional scaling analysis of experimental variables

**Figure 9:**
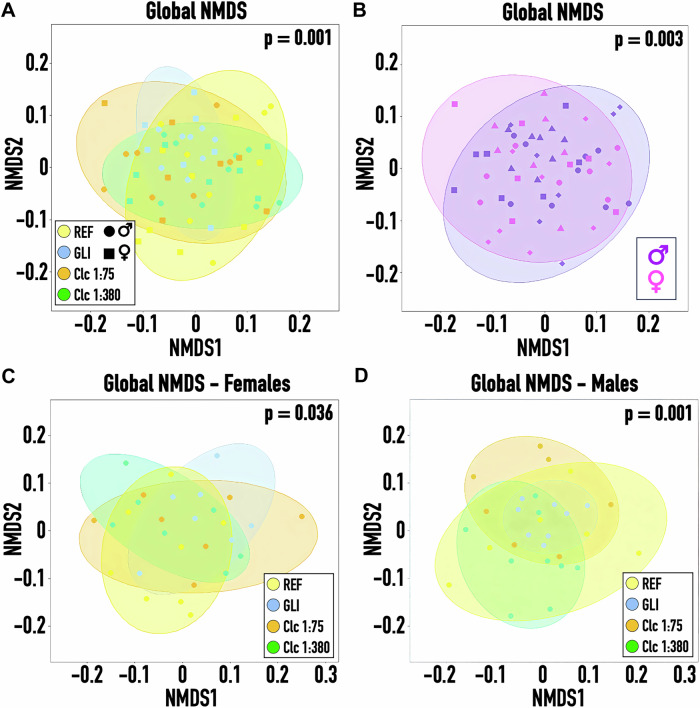
Comprehensive non-metric multidimensional scaling analysis of all experimental parameters. (**A**–**D**) Global NMDS analysis (see Statistical and data analysis) of combined data from the REF, GLI, Clc 1:380 and Clc 1:75 groups (*n* = 14/group, equally divided between females and males;*n* = 7/group and sex). The overall dataset includes auto-Ab levels in gut wash and plasma, plasma cytokines and Igs, blood haematological profile, gliadin levels in the stomach and small intestine, body weight change, chow and water consumption, body mass index and Lee index, faecal humidity and pH, organ weight, morphometric and histological measurements, mesenteric lymph-node lymphocyte populations and caecal microbiota composition. The results are depicted by group (**A**), sex (**B**), females alone (**C**) and males alone (**D**). Group differences were assessed using the analysis of similarities (*ANOSIM*) test. Statistical significance was set at *p* < 0.05. Source data are available online for this figure.

To evaluate the overall impact of Clc in gluten-fed DQ8-D^d^-villin-IL-15tg mice, we conducted a global NMDS analysis incorporating a comprehensive set of experimental variables, including gut and plasma auto-Ab levels, plasma cytokines and immunoglobulins, haematological profiles, gliadin concentrations in the stomach and small intestine, body-weight changes, food and water intake, body-mass index and Lee index, faecal humidity and pH, organ weights, morphometric and histological parameters, MLN lymphocyte populations and caecal microbiota composition (Fig. 9). The analysis revealed distinct clustering by treatment group across all animals (Fig. 9A; *p* = 0.001), along with sex-specific differences at the global level (Fig. 9B; *p* = 0.003), in females (Fig. 9C; *p* = 0.036), and in males (Fig. 9D; *p* = 0.001). Pairwise comparisons confirmed significant differences between REF and GLI, REF and Clc 1:380, and GLI and Clc 1:380 groups across all animals and within each sex (all *p* = 0.001–0.003).

In summary, NMDS analysis demonstrates that Clc treatment induces a robust and multidimensional shift in the physiological, immunological, and microbial profiles of gluten-fed DQ8-D^d^-villin-IL-15tg mice, underscoring its therapeutic potential in CD.

## Discussion

We developed Clc, a novel candidate glutenase rationally re-engineered from the pitcher plant enzyme Np. A single point mutation (C^334^V) improved recombinant expression in human cells, enhanced protein stability, prevented dimerisation—previously responsible for yield losses in Np—and increased peptidolytic activity by 20%. In vitro assays confirmed that Clc efficiently cleaved the immunogenic 33-mer peptide into three discrete fragments and hydrolysed a fluorogenic peptide containing the QPQL motif, highly resistant to GIT peptidases. Clc displayed strict proline specificity at the **P**_**1**_ position, consistent with its classification as a PEP, with optimal activity at pH 2–3 and negligible activity at neutral pH. This acid-activity profile makes Clc particularly well-suited for prandial gastric digestion while limiting downstream effects. Active Clc was readily generated from its zymogen (pClc) via auto-activation at pH 3 by pro-domain removal. The enzyme retained full activity after freezing and lyophilisation. Structural analysis confirmed a robust scaffold with a substrate-binding cleft tailored to QPQL motifs and two co-catalytic glutamate residues, classifying Clc within the eelysin family of glutamate peptidases.

ELISA analysis with G12 and R5 mAb pairs showed that Clc efficiently degraded GIPs in vitro in complex food matrices (gliadin and wheat flour) at low enzyme:gliadin ratios (1:50–1:500). Clc acted synergistically with pepsin under simulated gastric conditions and resisted pepsin degradation. Compared with the glutenase candidate AN–PEP, Clc removed ≈99% of GIPs from wheat flour and gliadin, versus ≈66% by AN–PEP. In a dynamic gastrointestinal simulator mimicking human digestion, pClc at 1:250 (1:10,000 relative to flour), following activation by simulated gastric fluid, reduced GIP by ≈99% in the stomach phase, while remaining inactive during oral and intestinal phases, consistent with its pH-dependent profile.

Previous work showed that the gliadin-derived 33-mer peptide elicits immune responses in macrophages (Herrera et al, 2018). Here, both mouse and rat macrophages stimulated with the intact 33-mer—but not its Clc-digested fragments—produced TNF-α. The same effect was observed for IL-6 in rat macrophages. Similarly, duodenal biopsies from adult CD patients (Marsh 1–3 A) secreted significantly higher levels of IL-17α, IFN-γ, GM-CSF, IL-18 and IL-2 when exposed to the intact 33-mer versus Clc-derived fragments. These cytokines, including IL-2, are known to be strongly upregulated upon gluten reintroduction in CD patients on a GFD (Goel et al, 2020; Tye-Din et al, 2019). Collectively, these results demonstrate that Clc abolishes the inflammatory and immunostimulatory effects of the 33-mer, suggesting its potential to mitigate gluten-induced immune activation.

In vivo, Clc demonstrated resilience to the harsh murine GIT environment in C57BL/6J mice, achieving ≈72% gliadin degradation in the stomach in an acute assay at a low enzyme:gliadin ratio (1:350), a key limitation in alternative glutenase development (Dunaevsky et al, 2021). Using the DQ8-D^d^-villin-IL-15tg mouse model, currently the most relevant transgenic model for CD (Abadie et al, 2022; Abadie et al, 2020), we confirmed Clc’s efficacy in the GIT. When gliadin-challenged animals received Clc at ratios of 1:75 or 1:380, immunological and pathophysiological alterations were significantly reduced compared with GLI mice (gliadin-challenged only) and approached those of GFD-fed controls (REF). These ratios actually underestimate true values, as gliadin from the normal diet was not considered due to the difficulty of quantifying intake in mice fed ad libitum.

Diarrhoea, a hallmark CD symptom, was assessed via faecal weight and humidity and was partially reversed by Clc. Histological analyses showed marked villous and crypt distortion in GLI mice, which was largely alleviated by Clc. The slightly reduced effect in males on the 25-day ED2 protocol may reflect sex-based differences in CD onset, as reported for women compared to men (Ciacci et al, 1995).

CD is characterised by a predominant Th1-mediated response and systemic inflammation (Bellanti et al, 2003). Consistent with this, GLI mice displayed an elevated Th1/Th2 ratio, which was mitigated by Clc.

Treatment also normalised IgG3/IgG and IgG2b/IgG ratios altered in GLI mice; the IgG3 increase mirrors patterns in human CD (Uhde et al, 2020), whereas IgG2b is murine-specific. GLI mice also had elevated anti-gliadin and anti-TG2 Ab titres, as previously described (Abadie et al, 2020), both substantially reduced by Clc—especially anti-TG2 IgA in females. MLN lymphocyte analysis revealed no major changes in lymphocyte subsets, as reported for other GIT autoimmune conditions (Postovalova et al, 2016), except for shifts in CD8α⁻CD4⁺ T cells and CD8α⁻CD4⁻ NKT cells in Clc-treated mice. Functionally, Clc prevented gliadin-induced reductions in IL-2 and IFN-γ in total lymphocytes, preserved IL-2 in T and NKT cells, and restored the IL-2/IFN-γ ratio in B cells. It also mitigated alterations in CD44 and CD127 expression, which are key receptors in adhesion, migration and survival.

Given the well-established association between CD and gut dysbiosis (Cenit et al, 2015), we profiled caecal microbiota in treated and untreated DQ8-D^d^-villin-IL-15tg mice. Analysis of β-diversity revealed significant shifts between REF and GLI, consistent with dysbiosis. Specifically, GLI mice showed increases in Actinobacteriota and Campilobacterota, and reductions in Cyanobacteria, Desulfobacterota, Patescibacteria and Verrucomicrobiota. Clc prevented most of these alterations, except for changes in Cyanobacteria and Desulfobacterota. At the genus level, Clc increased beneficial taxa such as *Lactobacillus*—containing gluten-processing species (Akobeng et al, 2020)—and *Lactococcus*, while counteracting gluten-induced reductions in *Odoribacter*, *Alistipes* and *Clostridium sensu stricto 1*, all previously associated with CD. Clc also reduced the overrepresentation of potentially pathogenic genera, including *Desulfovibrio*, *Bilophila* and various *Lachnospiraceae* members. However, it did not restore reductions in *Lactobacillus johnsonii* and *Akkermansia*, the latter being associated to gluten consumption.

To investigate functional consequences, we assessed microbial metabolic pathway enrichment, an area scarcely studied in CD (Constante et al, 2022). Multiple pathways were significantly enriched in REF compared with GLI, consistent with findings in paediatric CD cohorts (Leonard et al, 2021). GLI mice exhibited reductions in lysine degradation, butyrate and propionate production, sulphur and purine metabolism, glyoxylate and dicarboxylate cycles, lipopolysaccharide biosynthesis, the citrate cycle and the pentose phosphate pathway, which are changes associated with impaired gut barrier function and immune regulation (Rasouli-Saravani et al, 2023). Lipid and vitamin metabolism pathways, including thiamine, biotin and lipoic acid, were also downregulated in GLI, mirroring transcriptomic data (Loberman-Nachum et al, 2019). While vitamin biosynthesis pathways remained suppressed after Clc treatment, lipid metabolism was partially preserved. Finally, purine metabolism, previously reported as upregulated in active CD (Leonard et al, 2019), was enriched in GLI compared with Clc-treated mice.

In conclusion, Clc is a novel, structurally robust glutenase that efficiently degrades GIPs in vitro and in vivo, while mitigating hallmark immune, histological, and microbiota alterations associated with CD, as confirmed by global NMDS analysis. It outperforms other candidate glutenases and presents a strong therapeutic profile for use alongside or in place of a GFD, as discussed for AN–PEP (Stefanolo et al, 2024). Beyond its translational potential, Clc offers a valuable tool for studying gluten metabolism and host–microbiome interactions in CD. Future clinical studies will be essential to confirm its efficacy in humans and its potential to improve quality of life for individuals with CD and related conditions, including irritable bowel syndrome and non-coeliac wheat sensitivity.

## Methods

**Table:** Reagents and tools table

Reagent/resource	Reference or source	Identifier or catalogue number
**Experimental models**		
Lewis rats	Janvier Labs	LEW/Rj
C57BL/6 J mice	Janvier Labs	C57BL/6J
DQ8-D^d^-villin-IL-15tg mice	University of Chicago	NA
**Recombinant DNA**		
pClc–XGR3	GenArt	Custom gene synthesis
Neprosin	Genscript	Custom gene synthesis
**Antibodies**		
Aquagrant gluten G12	Romer Labs	033645096
Ridascreen® Gliadin	R-Biopharm AG	Art. No. R7001
Mouse Antibody Isotyping Panel 7-Plex	Invitrogen	EPX070-20816-901
ProcartaPlex Mouse and Rat Mix & Match Panel	Invitrogen	(MAN0025393)
ProcartaPlex Human Combinable Panel	Invitrogen	(MAN0028348)
ProcartaPlex Mouse Essential Th1/Th2 Panel 6-Plex	Invitrogen	EPX060-20831-901
Mouse Anti-gliadin IgA ELISA kit	AMSBIO	AMS.E03A2074
Mouse Anti-gliadin IgG ELISA kit	AMSBIO	AMS.E03A2073
Anti-transglutaminase-2 (TG2) IgA ELISA	AMSBIO	AMS.E03A2131
Mouse anti-CD3-APC/Cyanine7	BioLegend	100221
Mouse anti-CD4-PerCP/Cyanine5.5	BioLegend	100539
Mouse anti-CD8α-FITC	BioLegend	100803
Mouse anti-CD44-PE	BioLegend	103023
Mouse anti-CD62L-APC	BioLegend	104411
Mouse anti-CD127(IL-7Rα)-PE/Dazzle594	BioLegend	135031
Mouse anti-CD19-SuperBright 780	Thermo Fisher Scientific	78-0193-82
Mouse anti-CD314/NKG2D-SuperBright 600	Thermo Fisher Scientific	63-5882-82
Purified rat anti-mouse CD16/CD32	BD Biosciences	553141
Mouse anti-IL-2-BrilliantViolet 711	BioLegend	121125
Mouse anti-IFNγ-BrilliantViolet 421	BioLegend	505829
Mouse anti-HLA-DR/DP/DQ-FITC	BD Bioscience	562008
Mouse anti-MHC-II-Alexa-Fluor-700	Thermo Fisher Scientific	56-5321-80
Mouse anti-CD45-PE	Thermo Fisher Scientific	12-0451-81
ANTI-MO MCH II IA/IE NR	Fisher Scientific	17676975
Mouse TNF-alpha Sandwich ELISA Kit Datasheet	Proteintech	KE10002
**Oligonucleotides and other sequence-based reagents**		
33-mer	GenScript	Custom synthesised peptides
9-mer, 7-mer, 3-mer gliadin fragments	GenScript	Custom synthesised peptides
Random control peptides (6-mer, 8-mer)	GenScript	Custom synthesised peptides
Mca–Q–P–Q–L–Dpa–A–R–NH_2_	GenScript	Custom synthesised peptides
**Chemicals, Enzymes and other reagents**		
Wheat gliadin	Sigma-Aldrich	G3375
PWG-gliadin	Working Group on Prolamin Analysis and Toxicity	Not applicable. https://www.wgpat.com/pwggliadin.html
GliadinX (AN–PEP)	GliadinX	https://www.gliadinx.com
És farina de GIRONA (Farina de Blat)	Supermercat Bon Preu Esclat	https://farineracoromina.com/en/about; Lot No. L23004, Feb/24
RPMI-1640	Merck	R0883
L-glutamine	Thermo Fisher Scientific	G7513-100ML
streptomycin-penicillin	Merck	11548876
Lipopolysaccharide	Merck	L4391-1MG
Foetal bovine serum (FBS)	Merck	F0804-500ML
Ketamine (Ketabel 100 mg/mL)	Merial Laboratories	4052 ESP
Xylazine (Rompun 20 mg/mL)	Bayer	1977 ESP
Neo-Clear	Merck	1.09843
Paraplast plus for tissue embedding (Paraffin)	Merck	P3683-1KG
Roche cOmplete ULTRA EDTA free	Sigma-Aldrich	6538282001
Glycine 99.5%	Panreac AppliChem	A1067,5000
SYPRO Orange protein stain	Thermo Fisher Scientific	S6651
**Software**		
GraphPad Prism	GraphPad Software	v. 8
IBM SPSS Statistics	IBM	v. 30
R	R Foundation	v. 4.5.1 (2025-06-13 ucrt)
Rstudio	Posit Software, PBC	2025.09.1
ImageJ	Wayne Rasband and contributors	v. 1.54 G
FlowJo	BD Life Sciences	v. 11
flexControl software package	Bruker Daltonics	v. 3.4
UNICORN™ 7	Cytiva	v.7.1
Phenix suite	Phenix	v. 2.0
**Other**		
Simgi® dynamic gastrointestinal simulator	CIAL-CSIC	NA
Aurora flow cytometer	Cytek Biosciences	NA
BX41 microscope with an Olympus XC50 camera	Olympus	BX41
Gluten-free AIN-76A Purified Diet	Envigo	Cat. No. 170481
Global 18% Protein Rodent Diet	Teklad	2018
EDTA-coated Microvette tubes	Sarstedt	20.1288
Spincell 3 haematology analyser	Monlab	Ref. 5006101
40-μm mesh cell strainer	Thermo Fisher Scientific	22363547
Countess 3 automated cell counter	Invitrogen	Cat. No. AMQAX2000
Megafufe centrifuge	Heraeus	1.0 R
Kimble Cordless pellet pestle	Sigma-Aldrich	Z359971
22 G tube	Asico	K7-3550
Microplate reader	LabSystems	352 Multiskan MS
MAGPIX cytometer	Luminex	MAGPIX-XPON4.1-CEIVD
SuperFrost Plus adhesion slides	Thermo Fisher Scientific	1255015
Ni-NTA HisPur	Thermo Fisher Scientific	88221
ÄKTA pure™ 150 M	Cytiva	29046694
Superdex 75 increase 10/300 GL	Cytiva	29148721
PD-10 columns	Cytiva	17085101
Alpha 1–4 LD vacuum freeze dryer	Martin Christ	NA
iCycler iQ5 real-time PCR detection system	Bio-Rad	NA
AutoFLEX III MALDI-TOF mass spectrometer	Bruker Daltonics	NA
ground steel MALDI target plate	Bruker Daltonics	NA
96-well UV transparent plates	Thermo Fisher	8404
Synergy HTX microplate reader	BioTek	NA
QExactive Plus mass spectrometer	Thermo Fisher Scientific	NA

### Protein production and purification

The coding sequence for pNp (residues R^25^–Q^380^, UniProt C0HLV2) from *Nepenthes × ventrata* was previously cloned into a modified pCMV-Sport6 plasmid, yielding pS6-proNEP (del Amo-Maestro et al, 2022). This construct included a C-terminal His_6_-tag (residues AIAHHHHHH) for immobilised metal affinity chromatography (IMAC) and an N-terminal tetrapeptide (DLMV) preceding the pro-domain. pClc, corresponding to the C^334^V mutant of pNp, was generated via QuikChange site-directed mutagenesis (Phusion polymerase, Thermo Fisher Scientific [TFS]) using pS6-proNEP as a template and specific forward (5’–GCG TGA CCT GCA GGT CGT TGA TAC CTA TGG–3’) and reverse (5’–CCA TAG GTA TCA ACG ACC TGC AGG TCA CGC–3’) primers to yield the pS6-pro-celiacase plasmid. The glycosylation-deficient variant Clc–N^145^Q was produced by inverse PCR with non-overlapping primers (forward 5’–CAG GCG AGC CTG CAA GGC GCG–3’; reverse 5’–ACC GCC ATG AAT GTA CGC AAT AAC GCC–3’), followed by phosphorylation and ligation. A synthetic gene encoding pClc–XGR3 (R^25^–Q^380^), featuring an eight-residue deletion (ΔY^78^–Y^85^) and 60 point mutations (R^25^K, S^26^T, I^27^L, Q^28^N, R^30^E, A^32^S, K^36^R, T^41^S, V^49^M, L^65^K, H^69^P, T^70^S, P^75^G, S^77^G, E^89^K, P^91^S, N^95^S, K^100^E, G^101^V, T^111^S, L^113^V, K^117^R, N^161^T, N^166^D, G^167^K, S^182^T, S^183^N, T^215^S, T^217^S, T^230^V, Q^241^K, V^244^T, G^247^A, Q^248^E, G^271^S, T^272^V, G^279^S, S^284^T, N^287^E, T^304^S, F^306^Y, T^320^R, K^325^R, C^334^V, V^335^T, T^337^E, Y^338^R, N^340^Q, V^341^T, S^343^T, T^345^P, A^346^T, N^347^E, S^348^K, N^357^Q, N^360^K, Q^362^E, Q^365^E, S^367^K and S^368^K), was obtained from GenArt (TFS) and cloned into pCMV-Sport6 via *Eco91I* and *AsiSI* sites, preserving the same flanking sequences as pS6-proNEP and pS6-pro-celiacase.

Recombinant proteins were expressed in Expi293F cells (TFS) cultured in FreeStyle F17 expression medium (Gibco), which were transfected at ≈1×10^6^ cells/mL using polyethyleneimine. After ≈72 h, conditioned medium was harvested and subjected to batch IMAC using nickel nitrilotriacetic acid resin (Invitrogen) with peptidase inhibitors (Roche cOmplete), followed by SEC on a Superdex 75 Increase 10/300 GL column. Purity was assessed by 14% Tris-glycine SDS–PAGE using PageRuler Plus (10–250 kDa; TFS) or Blue Star PLUS (9–245 kDa; Nippon Genetics Europe) markers. Proteins were lyophilised in an Alpha 1–4 LD vacuum freeze dryer (Martin Christ), stored at −80 °C, and reconstituted in Milli-Q water prior to use. AN–PEP from GliadinX capsules (https://www.gliadinx.com) was directly dissolved in buffer, and its purity was confirmed by SDS–PAGE.

### Autolytic activation and disulfide-mediated dimerisation of neprosin

Mature Clc and Np were obtained by autolytic activation of the zymogens, following the protocol established for pNp (del Amo-Maestro et al, 2022). Briefly, proteins were buffer-exchanged after IMAC into 20 mM Tris·HCl pH 7.5, 250 mM sodium chloride using PD-10 columns (GE Healthcare) at room temperature. The eluate was then mixed 1:1 with 100 mM glycine pH 2.5 and incubated at 37 °C for up to 16 h prior to visualisation by 20% SDS–PAGE. Next, Np and pNp were buffer exchanged to 20 mM Tris·HCl pH 7.5, 150 mM sodium chloride to study intermolecular disulfide formation, which was verified by 20% SDS–PAGE analysis at pH 2.5 and pH 7.5 in non-reducing conditions.

### Protein stability assays

Thermostability of pClc and Clc was assessed by differential scanning fluorimetry using an iCycler iQ5 real-time PCR detection system (Bio-Rad). Samples (50 μL, 0.8 mg/mL) in 20 mM Tris·HCl pH 7.5, 150 mM sodium chloride were supplemented with 5× SYPRO Orange protein stain (TFS) and heated from 25 to 95 °C at a rate of 1 °C/min. The temperature of mid-transition was determined from the midpoint of the fluorescence transition curve using the GraphPad Prism software (Swift, 1997).

### Activity profiling and stability

Proteolytic activity of AN–PEP, Clc, Np, and their variants was assessed with the fluorogenic peptide FS6-QPQL (Mca–Q–P–Q–L–Dpa–A–R–NH_2_; GenScript) at 10 μM. Enzyme concentrations were determined spectrophotometrically at 280 nm using a BioDrop-DUO micro-volume UV/Vis spectrophotometer (Biochrom) and theoretical extinction coefficients. Reactions were performed in triplicate and monitored for fluorescence (λₑₓ = 325 nm, λₑ_m_ = 393 nm) on a Synergy H1 microplate reader (BioTek), with endpoint readings taken after 30 min. For general activity assays, enzymes were incubated in 100 mM glycine pH 3.0, 150 mM sodium chloride at 37 °C. For pH profiling, 0.15 μM celiacase was incubated with substrate in 100 μL McIlvaine buffer (pH 2–8 (McIlvaine, 1921)). For temperature profiling, 0.15 μM Clc was assayed in 100 mM glycine pH 3.0, 150 mM sodium chloride at 22–45 °C. For stability analysis, activity was assessed after storage at 4 °C for 1 week, flash-freezing in liquid nitrogen and storage at −80 °C, or after lyophilisation and storage at −80 °C.

The proteolytic activity of Clc was further evaluated against the protein substrates albumin (15 mg/mL), casein (15 mg/mL), soy protein (10 mg/mL) and mucin II (20 mg/mL). Digestion reactions were performed at an enzyme:substrate ratio of 1:420 (w/w) in glycine buffer (pH 2.5) and incubated at 37 °C for 16 h. The resulting proteolytic profiles were analysed by 14–20% SDS–PAGE.

AN–PEP was titrated and its enzyme units (U) were determined using the peptide Z-G-P-pNA (1 mM), monitoring absorbance at 410 nm for 10 min at 37 °C. One unit (U) of activity was defined as the amount of enzyme required to release 1 μmol/min of *p*-nitroaniline. The results indicated that one GliadinX capsule contained ≈7 U, in contrast to the value indicated by the manufacturer (194,000 PPI; ≈11 U). AN–PEP was also tested against FS6-QPQL under the same conditions as Clc.

### In vitro cleavage of the 33-mer peptide

The 33-mer peptide from wheat α-gliadin (LQL QPF PQP QLP YPQ PQL PYP QPQ LPY PQP QPF; theoretical Mw = 3911 Da; see Appendix Fig. S1) was purchased from GenScript. Peptide cleavage by Clc was monitored on an AutoFLEX III MALDI-TOF mass spectrometer (Bruker Daltonics), equipped with a pulsed nitrogen laser (λ = 337 nm) and operated via the *flexControl* software package (Bruker Daltonics). The peptide was dissolved in water at 5 mM and stored at −20 °C. Cleavage reactions were conducted with 250 μM substrate in 100 mM glycine pH 3.0 at 37 °C, in the presence of 0.5 μM Clc and/or 10 μM pepsin. Reactions were quenched at 0, 10 and 20 min by adding 1 μL of 2 M Tris·HCl pH 8.8 per 50 μL of reaction mixture. Samples were then diluted 1:10 with water, mixed 1:1 with 2,5-dihydroxybenzoic acid matrix (10 mg/mL in 30% acetonitrile and 0.1% trifluoroacetic acid), and spotted onto a ground steel MALDI target plate (Bruker Daltonics). After air-drying at room temperature, spectra were acquired in positive reflectron mode at 21 kV total acceleration voltage, focusing on a mass range of 200–4000 *m/z*. Each spectrum was averaged from 200 random laser shots and analysed with the *flexAnalysis* software (Bruker Daltonics). Cleavage resulted in 9-mer, 7-mer and 3-mer peptide fragments at 1:3:1 molar ratio (Appendix Fig. S1).

### Turbidimetric assays

Commercial wheat gliadin (Sigma-Aldrich, Cat. No. G3375) was prepared as a 10 mg/mL slurry in 100 mM glycine pH 3.0, 150 mM sodium chloride, and sonicated to aid suspension. Gliadin degradation assays were conducted in 200-μL reaction volumes by adding varying concentrations of enzymes: pepsin from porcine gastric mucosa (Fluka; 0.05–10 μM), Clc (0.05–2 μM) or AN–PEP (0.05–2 μM). To assess synergistic effects, 0.5 μM pepsin was combined with 0.05–2 μM Clc or AN–PEP. Although the physiological concentration of pepsin in humans is ≈5 µM (Roberts et al, 2007), a tenfold lower concentration was chosen to enhance the effect of Clc and AN–PEP. Reactions were performed in 96-well plates (Corning) at 37 °C and monitored turbidimetrically using a Synergy HTX microplate reader (BioTek). Absorbance at 595 nm was recorded every 54 s for 120 min, with intermittent shaking to maintain homogeneity. Reactions were terminated by boiling in SDS sample buffer for 10 min, and quenched samples were analysed by Tris-glycine SDS–PAGE as described above.

### Protein composition of wheat flower and gliadin

The protein composition of a local Catalan wheat flour (*Farina de Girona*, https://farineracoromina.com/en/about; Lot No. L23004, Feb/24) and commercial wheat gliadin (Sigma-Aldrich, Cat. No. G3375) was determined according to (Gabler and Scherf, 2020; Jahn et al, 2024). For wheat flour (100 mg), the albumin/globulin fraction was extracted in two steps with 1 mL of 67 mM sodium/potassium phosphate pH 7.6, 400 mM sodium chloride. Gliadins were subsequently extracted in three steps with 0.5 mL of 60% (v/v) ethanol, and glutenins with two steps of 1 mL of 50% (v/v) propanol, 2 M urea, 1% (w/v) dithiothreitol, and 50 mM Tris·HCl pH 7.5. Each extraction involved vortexing (2 min), magnetic stirring (10 min at 22 °C for albumins/globulins and gliadins; 30 min at 60 °C for glutenins), and centrifugation (30 min, 25 °C, 3550×*g*). The respective supernatants were pooled and adjusted to 2 mL with the corresponding extraction solution. For commercial gliadin (20 mg), the albumin/globulin extraction step was omitted. Gliadins and glutenins were extracted in three steps with 1.5 mL of their respective solutions, followed by the same mixing, stirring, and centrifugation procedures. Supernatants were combined, adjusted to 5 mL, and filtered through a 0.45-µm regenerated cellulose membrane (Wicom). Samples were analysed by reversed-phase high-performance liquid chromatography as described (Xhaferaj and Scherf, 2024), and protein profiles were compared with PWG-gliadin from the Working Group on Prolamin Analysis and Toxicity (Arbeitsgemeinschaft Getreideforschung, Detmold (van Eckert et al, 2006)), which also served as the external calibration standard and contains 86.4% of gliadin. All extractions were performed and analysed in triplicate.

### Enzyme-linked immunosorbent assay quantification of gluten immunogenic peptides

The presence of GIPs derived from gluten/gliadin before and after degradation by Clc and AN–PEP was assessed using commercial wheat gliadin, PWG-gliadin, wheat flour (*Farina de Girona*), and a complex meal (2018 Global 18% Protein Rodent Diet; Teklad). Digestions were carried out at 37 °C for 2 h in 0.1 M glycine pH 2.5, with enzymes added at specified enzyme:gliadin ratios. Actual gliadin content was determined for each product (see Appendix Fig. S2; Appendix Table S4, and (van Eckert et al, 2006)). Additionally, samples from the dynamic gastrointestinal simulator (section “Studies in a dynamic gastrointestinal simulator”) and murine GIT sections (section “Quantification of gluten immunogenic peptides, antibodies, and cytokines”) were also analysed through ELISA. Following alcoholic extraction (2–6 replicates per condition), GIPs were quantified with either the AgraQuant Gluten G12 ELISA kit (Romer Labs) or the Ridascreen R5 Gliadin ELISA kit (R-Biopharm AG), which recognise distinct GIP epitopes (Morón et al, 2008b; Osman et al, 2001) (see section “In vitro cleavage of gluten immunogenic peptides in complex matrices”), according to the manufacturers’ instructions.

### Proteomics-based protease specificity profiling, mass spectrometry and data analysis

Protease specificity was assessed via the Proteomic Identification of peptidase Cleavage Sites (PICS) method with trypsin- or GluC-digested reference peptide libraries, as previously described (Eckhard et al, 2020; Eckhard et al, 2016; Schilling et al, 2011b; Schilling et al, 2011c). Briefly, overnight cleavage assays were performed with either Clc or Clc–XGR3 (0.2 µg) on an *Escherichia coli*-derived library or commercial gliadin (20 µg) in 20 µL of 100 mM glycine buffer pH 2.5, 150 mM sodium chloride. For PICS profiling, 25 µg of peptide library was incubated at 37 °C with 0.5 µg of enzyme in 50 µL of either 100 mM glycine pH 2.5, 150 mM sodium chloride (active Clc due to acidic pH; heavy label) or 25 mM Tris·HCl pH 8.0, 150 mM sodium chloride (inactive Clc due to basic pH; light label). Reactions were chilled on ice, and cysteines were alkylated with iodoacetamide after addition of 100 mM triethylammonium bicarbonate pH 8.5. Peptides were labelled by reductive dimethylation with formaldehyde (light) or deuterated ^13^C-formaldehyde (heavy), and samples were pooled for further processing. All samples were purified on C18 columns (TFS), dried, and reconstituted in 1% acetonitrile, 0.1% formic acid for LC–MS/MS analysis on a QExactive Plus mass spectrometer (TFS). Data were acquired in data-dependent mode following established protocols (Blochl et al, 2021; Eckhard et al, 2020). Raw MS data were processed in *FragPipe* (Yu et al, 2023), with *MSFragger* (Kong et al, 2017) for peptide-spectrum matching, *MSBooster* (Yang et al, 2023) and *Percolator* (The et al, 2016) for enhanced identification, and *Philosopher* (da Veiga Leprevost et al, 2020) for statistical validation at a 1% false discovery rate. Searches were semi-specific against the *E. coli* K12 proteome (with contaminants) for PICS, and non-specific against the GluPro cereal prolamin database (Daly et al, 2020) for gliadin assays. Cleavage peptides required ≥2 peptide-spectrum matches for inclusion, and cleavage sites were reconstructed using *WebPICS* (Schilling et al, 2011a) or *FragPipe*’s extended peptide output. Specificity motifs were visualised with *iceLogo* (Colaert et al, 2009), showing statistically significant (*p* < 0.01) amino-acid enrichment (positive) or depletion (negative) compared to the reference proteome at non-prime (**P**_**6**_–**P**_**1**_, left; for peptidase substrate and active-site nomenclature, see (Gomis-Rüth et al, 2012; Schechter and Berger, 1967)) and prime (**P**_**1**_**’**–**P**_**6**_**’**, right) positions relative to the scissile **P**_**1**_–**P**_**1**_**’** bond (vertical dashed line).

### Crystallisation, diffraction data collection and structure analysis

pClc was auto-activated as described above, and the resulting mature enzyme was further purified by SEC in 20 mM Tris·HCl, pH 7.5, 150 mM sodium chloride. Crystallisation was performed by sitting-drop vapour diffusion at the IBMB Automated Crystallography Platform (https://www.ibmb.csic.es/en/platforms/automated-crystallographic-platform). Optimal crystals, with one protomer in the asymmetric unit, formed at 20 °C with protein at ≈16 mg/mL, using a reservoir solution containing 0.1 M Bis-Tris propane pH 6.5, 0.2 M sodium sulphate, 22% polyethylene glycol 3350. Crystals were harvested with cryo-loops (Molecular Dimensions), transferred briefly through cryo-buffer (reservoir solution supplemented with 20% [v/v] glycerol), and flash-vitrified in liquid nitrogen. Complete X-ray diffraction datasets were collected at 100 K on beamline ID23-1 of the ESRF Synchrotron (Grenoble, France), using an Eiger2-16M-CdTe pixel detector. Data were processed with *Xds* (Kabsch, 2010) and *Xscale* to 1.9 Å resolution. Data were converted to *Mtz*-format with *Xdsconv* for use in the *Phenix* (Liebschner et al, 2019) and *Ccp4* (Agirre et al, 2023) crystallographic software suites. Data collection and processing statistics are provided in Appendix Table S1.

The Clc structure was determined by Fourier synthesis using the *Refine* protocol from the *Phenix* suite, with Np (PDB entry 7ZVC) as the initial model. Subsequently, iterative cycles of manual model building in *Coot* (Casañal et al, 2020) alternated with crystallographic refinement with *Refine*, which included translation/libration/screw-motion refinement. The final Clc model comprised residues T^132^–Q^480^, C-terminal tag residues A I A H H H H, one tentative pentaglycine, one diethylene glycol molecule, a sulphate ion, a chloride ion, a Tris molecule, a glycerol molecule, and 185 solvent molecules. Two *N*-linked glycan chains were observed: NAG–FUC at N^145^ and NAG–NAG–BMA at N^152^ (sugar abbreviations and final model statistics are provided in Appendix Table S1). Validation was performed with *Phenix* and the *wwPDB Validation Service* (https://validate-rcsb-1.wwpdb.org/validservice), and the structure was deposited in the PDB under accession code 9QR8.

### Studies in a dynamic gastrointestinal simulator

The Simgi^®^ dynamic gastrointestinal simulator (DGS; https://cial.uam-csic.es/simgi (Cueva et al, 2015)) was employed to evaluate the effect of Clc on wheat flour digestion within a human GIT context. For this study, the stomach and small intestine compartments, each equipped with multiple input and output ports (Fig. 2H; Appendix Fig. S4), were used to dynamically model the oral phase (OP), gastric digestion (GD), and intestinal digestion (ID). Simulated salivary, gastric, and intestinal fluids were prepared under sterile conditions as previously described (Brodkorb et al, 2019). Simulated gastric juice was freshly prepared by supplementing simulated gastric fluid with pepsin (2000 U/mL; Sigma-Aldrich) and stored at 4 °C. Simulated pancreatic juice was produced by dissolving sodium bicarbonate (12.5 mg/mL; Sigma-Aldrich), bile salts (6 mg/mL; BD Difco), and porcine pancreatin (0.9 mg/mL; Sigma-Aldrich) in simulated intestinal fluid, followed by filtration through a 0.45-µm polyethersulfone membrane (Millipore).

Three distinct gluten-containing food models (GFMs) were evaluated under sterile conditions. Each GFM consisted of a slurry of 8 g of wheat flour (section “Enzyme-linked immunosorbent assay quantification of gluten immunogenic peptides”) in 80 mL of simulated salivary fluid (Brodkorb et al, 2019), supplemented with 10 mL of 20 mM Tris·HCl pH 7.5, 150 mM sodium chloride, and either no Clc (vehicle), Clc at 0.16 mg/mL (at 1:250 enzyme:gliadin ratio), or Clc at 0.80 mg/mL (1:50) (Fig. 2I). These ratios were calculated based on the experimentally determined gliadin content of the flour (Appendix Fig. S2). A total of nine gastrointestinal digestions were performed (three replicates per GFM), with slight modifications to established protocols (Tamargo et al, 2022; Tamargo et al, 2023). Before each run, the system was preconditioned with simulated gastric fluid (65 mL, pH 2.0) in the stomach compartment and simulated intestinal fluid (55 mL, pH 7.0) in the intestinal compartment. Digestion began with 90 mL of a GFM, representing OP, from which a 10 mL aliquot was immediately collected. The remaining 80 mL was introduced into the stomach together with 15 mL of simulated gastric juice via peristaltic movements, yielding a total gastric volume of 160 mL. GD proceeded with gradual acidification to pH 1.8. Subsequently, gastric emptying was simulated using the Elashoff method (Elashoff et al, 1982), after which a 100 mL sample was collected (GD). For the ID fraction, the remaining 60 mL of gastric digest was transferred to the intestinal compartment, combined with 40 mL of simulated pancreatic juice. The pH was adjusted to and maintained at 7.0 ± 0.2, and the mixture was incubated at 37 °C for 120 min with agitation (150 rpm) under anaerobic conditions, after which the entire final ID volume (≈155 mL) was collected (ID). Aliquots from each digestion stage (OP, GD and ID) were immediately stored at –80 °C for subsequent ELISA analysis using the G12 Ab, as described in section “Enzyme-linked immunosorbent assay quantification of gluten immunogenic peptides”.

### Ex vivo immune stimulation by the 33-mer and its cleavage fragments

Peritoneal macrophages were isolated from male C57BL/6 J (*n* = 10) and female Lewis rats (*n* = 24) as previously described (Estruel-Amades et al, 2020). Briefly, 10 or 40 mL of cold phosphate buffered saline at pH 7.2 (PBS) were injected into the abdominal cavity of mice or rats, respectively, followed by gentle massage for 2 min. The peritoneal fluid was collected, erythrocytes lysed, and the suspension centrifuged at 538×*g* for 10 min at 4 °C. The supernatant containing macrophages was resuspended in RPMI-1640 medium (Merck), supplemented with 10% v/v heat-inactivated foetal bovine serum (Merck), 100 IU/mL streptomycin-penicillin (Merck), and 2 mM L-glutamine (TFS). Cells were plated at 10^6^ cells/well and cultured overnight (5% CO_2_, 37 °C), then stimulated for 24 h at either a low (0.25 mM) or high (4.25 mM) concentration of the 33-mer peptide, or its Clc-derived fragments (9-mer, 7-mer and 3-mer; see section “In vitro cleavage of the 33-mer peptide” and Appendix Fig. S1; purchased from GenScript). Lipopolysaccharide (≈0.1 µg/mL) and vehicle served as positive and negative controls, respectively. Two random peptides–a 6-mer (TATRGG) and an 8-mer (ATALAKVE)–were included as additional negative controls. Given the 1:3:1 molar ratio of the 33-mer fragments following Clc cleavage (Appendix Fig. S1), they were tested, individually or in combination (“fragments” in Fig. 3), at corresponding relative concentrations: 0.05, 0.15, 0.05 mM (low) and 0.85, 2.55 and 0.85 mM (high). Following stimulation, plates were centrifuged at 538×*g* for 5 min, and supernatants were collected for cytokine response analysis. The macrophages used in this study were routinely tested and confirmed to be free of mycoplasma contamination. This study was approved by the Ethics Committee for Animal Experimentation of the University of Barcelona (CEEA-UB; Ref. OT-240/19 [rats] and Ref. 186/20 [mice]).

Duodenal biopsies were obtained from nine diagnosed CD patients (five women and four man, aged 31–65 years), classified as Marsh 1–3A, which were kindly provided by Dr. Fernando Fernández Bañares (Digestive Service, Mútua de Terrassa University Hospital, Barcelona). Within 2 h of collection and transport, biopsies were immersed in FBS-supplemented RPMI-1640 medium with foetal bovine serum and stimulated at 5% CO_2_ and 37 °C for 24 h with lipopolysaccharide, vehicle, the 33-mer, or the fragments at either low (0.5 mM; six patients, 4–5 biopsies/patient) or high (17 mM; three patients, 4–8 biopsies/patient) concentrations, after which the cytokine response was assessed. This study was approved by the Ethics Committee for Investigation with Medicinal Products (CEIm) of the Fundació Assistencial Mútua de Terrassa (Ref. P21-143). All participants provided written informed consent prior to enrolment. All procedures involving human participants were conducted in accordance with the ethical standards of the World Medical Association Declaration of Helsinki and the principles outlined in the US Department of Health and Human Services Belmont Report. Blinding was not performed, as it was not applicable to the study design.

### Animal care and maintenance

Inbred C57BL/6 mice from Janvier Labs and transgenic DQ8-D^d^-villin-IL-15tg mice, the progenitors of which were kindly provided by V. Abadie from the University of Chicago (Abadie et al, 2020), were housed in polycarbonate cages at the Animal Experimentation Unit at the Faculty of Pharmacy of the University of Barcelona (https://www.ub.edu/portal/web/farmacia/servei-d-experimentacio-animal-ub-, ref. 9900030), under controlled temperature and humidity with a 12:12 h light–dark cycle. Mice had ad libitum access to water and chow, and were acclimatised for at least 1 week prior to experimentation. Mice were fed either regular normal diet (2018 Global 18% Protein Rodent Diet; Teklad) or its equivalent GFD (Gluten-free AIN-76A Purified Diet; Envigo, Cat. No. 170481). This study is reported in accordance with the ARRIVE guidelines.

For the transgenic mice, presence of *D*^*d*^*-IL-15* and *villin-IL-15* transgenes was assessed in all animals by multiplex PCR using ear tissue, and both C57BL/6 J and BALB/c mice were used as negative controls. Briefly, samples were lysed and amplified with Platinum Direct PCR Universal Master Mix and Platinum GC Enhancer (TFS), along with forward primers (*D*^*d*^*-IL-15*: 5’-TGATATCGAATTCGGCTTG-3’; *villin-IL-15*: 5’-AGTTTCCCTTCTTCCTCTGG-3’), a common reverse primer (*IL-15*: 5’-GACGTGTTGATGAACATTTGG-3’), and nuclease-free water (TFS). PCR was performed for 40 cycles under thermal conditions as recommended by the manufacturer. Products were resolved on a 1% agarose gel at 90 V for 1 h. Both transgenes yielded bands of about 450 bp. To confirm the DQ8 transgenic phenotype, ≈5×10^5^ mesenteric lymph-node lymphocytes were stained extracellularly as reported (Ruiz-Iglesias et al, 2022), with the fluorochrome-conjugated anti-mouse mAbs anti-HLA-DR/DP/DQ-FITC (BD Bioscience), anti-MHC-II-Alexa-Fluor-700, and anti-CD45-PE (both TFS), and assessed by flow cytometry analysis using an Aurora cytometer (Cytek Biosciences) at the Flow Cytometry Unit of the Scientific and Technological Centres of the University of Barcelona (CCiTUB; https://www.ccit.ub.edu/EN).

### Experimental design 1 (ED1)

Ten-week-old C57BL/6 mice on normal diet were placed on GFD 1 day before the experiment and fasted for 1 h prior to administration to minimise gastrointestinal content interference. Mice were randomly assigned to two groups (*n* = 8 per group, four females and four males each). Each received 50 µL of PWG-gliadin (80 mg/mL in 1:5 ethanol:water), preceded by either 50 µL of vehicle (20 µM Tris-buffered saline pH 7.5, 150 µM sodium chloride) or 50 µL of Clc in vehicle at a Clc:gliadin ratio of 1:350. These ratios do not account for the gliadin present in the chow, which represents about 40–50% of the gluten content; gluten itself makes up roughly 70–80% of the total grain protein of the normal diet, according to the manufacturer. After 2.5 h (Padmanabhan et al, 2013), animals were anaesthetised intramuscularly with ketamine (90 mg/kg, Merial Laboratories) and xylazine (10 mg/kg, Bayer). Digestive content was collected from the stomach, proximal and distal small intestine, caecum, and rectum, and stored for GIP quantification via ELISA using the G12 mAb pair (see sections “Enzyme-linked immunosorbent assay quantification of gluten immunogenic peptides” and “Quantification of gluten immunogenic peptides, antibodies, and cytokines”). The study was approved by the CEEA-UB (Ref. 186/20).

### Experimental design 2 (ED2)

Nine-week-old DQ8-D^d^-villin-IL-15tg mice on GFD were divided into four groups (*n* = 14 per group, seven females and seven males each). The REF group continued on GFD and received 100 μL of PBS (vehicle) via oral gavage with a 100-µL Hamilton syringe and 22 G tube (Asico). The GLI group was fed a gluten-containing diet supplemented with 20 mg of commercial wheat gliadin (section “Enzyme-linked immunosorbent assay quantification of gluten immunogenic peptides”) three times weekly on alternate days by gavage (Abadie et al, 2022). The two treatment groups (Clc 1:380 and Clc1:75) followed the GLI protocol but further received Clc at 1:380 or 1:75 enzyme:gliadin ratios, administered 5 min prior to gliadin. As for ED1, these ratios do not account for the gliadin present in the chow. At the end of ED2 on day 25, mice were fasted for 1.5 h, anaesthetised intramuscularly with ketamine (90 mg/kg) and xylazine (10 mg/kg), and exsanguinated by cardiac puncture. Blood was collected in EDTA-coated Microvette tubes (Sarstedt). One aliquot was analysed immediately with a Spincell 3 haematology analyser (Monlab), while others were centrifuged at 23,000×*g* for 10 min at room temperature in a Sigma 112 mini centrifuge to obtain plasma. MLNs were processed through a sterile 40-μm mesh cell strainer (TFS) to isolate lymphocytes, whose viability and count were assessed with a Countess 3 automated cell counter (Invitrogen), as previously described (Estruel-Amades et al, 2019). The stomach, small intestine (proximal and distal segments), caecum, and rectum were excised, weighed, and their digestive content collected and stored at room temperature for further analysis. Given that villous atrophy is most pronounced in the distal small intestine in this mouse model (Abadie et al, 2022; Abadie et al, 2020), segments of 2 cm were obtained from distal intestine and fixed in 4% PBS-paraformaldehyde (ITW Reagents) for histology. Additionally, distal segments were weighed, minced, and incubated in 4 mL PBS at 37 °C and 750 rpm for 10 min in an orbital shaker. Samples were centrifuged at 538×*g* for 10 min at 4 °C in a Megafufe 1.0 R centrifuge (Heraeus), and the supernatant (gut wash) was collected for further analysis. The study was approved by the CEEA-UB (Ref. 186/20).

### Quantification of gluten immunogenic peptides, antibodies and cytokines

The concentrations of cytokines IL-1β, IL-2, IL-5, IL-6, IL-10, IL-12p70, IL-13, IL-17α, IFN-γ, GM-CSF, TNF-α, IL-18 and IL-22 in macrophage supernatants from Lewis rats were determined using ELISA kits (BD Biosciences) and the ProcartaPlex Mouse and Rat Mix & Match Panel (Invitrogen). For mouse macrophages, TNF-α concentrations in the supernatants were quantified using an ELISA kit (Proteintech). The same cytokines were analysed in supernatants from stimulated biopsies of CD patients with the ProcartaPlex Human Combinable Panel (Invitrogen). For ED1 and ED2, contents from the distinct GIT segments (sections “Experimental design 1 (ED1)” and “Experimental design 2 (ED2)”) were homogenised in PBS with a cordless pellet pestle (Kimble) to a final concentration of 200 mg/mL. Samples underwent alcoholic extraction for ELISA analysis with the G12 and R5 mAb pairs, as detailed in section “Enzyme-linked immunosorbent assay quantification of gluten immunogenic peptides”. In ED2, plasma and gut wash samples were tested for anti-TG2 IgA, anti-gliadin IgA, and anti-gliadin IgG using specific ELISA kits (from Amsbio). Moreover, Ig isotypes and subclasses (IgA, IgE, IgG1, IgG2a, IgG2b, IgG3 and IgM) were quantified with the Mouse Antibody Isotyping Panel 7-Plex (Invitrogen). Plasma and intestinal cytokines IL-2, IL-4, IL-5, IL-6, IL-12p70, IFN-γ and TNF-α were measured using the ProcartaPlex Mouse Essential Th1/Th2 Panel 6-Plex. The Th1/Th2 ratio was calculated as IgG2a/IgG1. All assays were performed according to the manufacturers’ protocols. Data were acquired on a 352 Multiskan MS microplate reader (LabSystems) or a MAGPIX cytometer (Luminex) at the CCiTUB Flow Cytometry Unit.

### Histology

Histological analysis of distal intestine samples from ED2 was conducted as previously described (Sáez-Fuertes et al, 2024). Briefly, tissues were sequentially dehydrated in increasing ethanol concentrations (70, 96, and 100%) containing Neo-Clear (Merck)—as xylene substitute—and paraffin (Merck), sectioned at 5 μm, and mounted on SuperFrost Plus adhesion slides (TFS). Sections were stained with haematoxylin and eosin and examined under a bright-field Olympus BX41 microscope equipped with an Olympus XC50 camera at 40×, 200× or 1000× magnification. Villi height and width, and crypt depth were measured using *ImageJ* (Schneider et al, 2012). Villi area and the villous height-to-crypt depth ratio were subsequently calculated.

### Phenotyping of mesenteric lymph-node lymphocytes

MLN lymphocytes (≈5 × 10^5^) were stained for both extracellular and intracellular markers with anti-mouse monoclonal Abs, as previously described (Ruiz-Iglesias et al, 2022). For surface staining, the following fluorochrome-conjugated mAbs were employed with the corresponding dilutions: anti-CD3-APC/Cyanine7 (1/100), anti-CD4-PerCP/Cyanine5.5 (1/200), anti-CD8α-FITC (1/200), anti-CD44-PE (1/100), anti-CD62L-APC (1/200), anti-CD127(IL-7Rα)-PE/Dazzle594 (1/200) (all BioLegend), anti-CD19-SuperBright 780 (1/200) and anti-CD314/NKG2D-SuperBright 600 (1/20) (TFS). F_c_ receptors were blocked with purified rat anti-mouse CD16/CD32 (BD Biosciences) prior to mAb incubation and fixation. For intracellular staining, cells were permeabilized, incubated with anti-IL-2-BrilliantViolet 711 (1/50) and anti-IFNγ-BrilliantViolet 421 (1/75) (BioLegend), and subsequently fixed. Flow cytometry analysis was conducted at the CCiTUB Flow Cytometry Unit using an Aurora cytometer (Cytek Biosciences). Data were processed and gated with *FlowJo* (BD Life Sciences). Isotype-matched controls were included for each cell sample.

### Caecal microbiota profiling

Caecal samples from female mice in the REF, GLI, and Clc 1:380 groups (see section “Experimental design 2 (ED2)”) were analysed (*n* = 5 per group) by Microomics Systems (Barcelona; www.microomics.com). Microbial composition was assessed via amplification and sequencing of the V3–V4 regions of the 16S rRNA gene using Illumina MiSeq (2 × 300 bp). Raw reads were processed with *QIIME 2* (Bolyen et al, 2019) and *Dada2* (Callahan et al, 2016). Data on phylotype and operational taxonomic unit were used to compute α-diversity (richness and evenness) and β-diversity (weighted-UniFrac). Richness and evenness were analysed with generalised linear models with the packages *MASS* (Venables and Ripley, 2002) and *glmmTMB* (https://github.com/glmmTMB/glmmTMB), respectively. For mixed models, *NBZIMM* (Zhang and Yi, 2020) and *betareg* (Cribari-Neto and Zeileis, 2010), respectively, were used instead. β-Diversity was visualised via principal coordinate analysis and ordination plots with the *R* software package (https://www.R-project.org), with group differences assessed by *PERMANOVA* and *ANOSIM*, and dispersion effects by *PERMDISP* (Anderson and Walsh, 2013). Taxonomic classification was performed using a Bayesian classifier (Wang et al, 2007) trained on the Silva database (Pruesse et al, 2007). Differential abundance was tested with negative binomial generalised linear models with *MASS* or *NBZIMM*. Taxa with <1% relative abundance across all groups were grouped accordingly. Linear discriminant analysis with *MASS* identified genera and species most associated with each treatment. Functional profiling was conducted with *PICRUSt2* (Douglas et al, 2020), placing phylotypes into a reference tree of ≈20,000 16S rRNA genes from the IMG database. Functional annotation was based on KEGG-database orthologs (Kanehisa et al, 2012). Minimal statistical significance was set at *p* ≤ 0.05.

### Statistical and data analysis

Unless otherwise specified, in vitro activity assays are presented as the mean ± standard error of the mean (SEM) from two replicates of four independent experiments. Statistical analyses were performed using one- or two-way *ANOVA* in GraphPad Prism (*p ≤ 0.05, **p ≤ 0.01, ***p ≤ 0.001, ****p ≤ 0.0001).

For cell and animal assays, *IBM SPSS Statistics* for Windows (IBM Corp.) was used. Normality and homogeneity of variance were assessed with the Shapiro–Wilk and Levene tests, respectively. Parametric data were analysed via one- or two-way *ANOVA* followed by Bonferroni post hoc tests. Non-parametric data were evaluated using the Kruskal–Wallis test with Dunn’s post hoc and Bonferroni correction. Correlations were assessed using Pearson or Spearman coefficients, depending on data distribution. Unless otherwise specified, results are presented as the mean ± SEM.

NMDS based on Bray–Curtis distances was performed with the *Vegan* package (Oksanen et al, 2025) within the *R* software package. Group differences were tested using *ANOSIM*, with pairwise *ANOSIM* for post hoc comparisons. Significance was set at *p* < 0.05. Asterisks denote significance levels as in the above in vitro assays. For pairwise comparisons: *p ≤ 0.05 vs. REF; #*p* ≤ 0.05 vs. GLI.

Samples were processed and analysed using the same predefined experimental workflow. Where applicable, samples were assigned to experimental groups according to predefined criteria; no formal randomisation was performed. Investigators were not blinded during sample processing and data analysis.

### Miscellaneous

Three-dimensional structure figures were prepared using *Chimera* (Huang et al, 2014). Molecular visualisations were further generated with commercial office productivity software, as well as with BioRender (https://www.biorender.com). During the preparation of this manuscript, the authors used large language models based on the *Transformer* deep neural architecture (Vaswani et al, 2017), strictly in order to improve language and grammar. After using these tools, the authors reviewed and edited the content as needed and take full responsibility for the content of the publication.

## Supplementary information

Appendix

Peer Review File

Source data Fig. 1

Source data Fig. 2

Source data Fig. 3

Source data Fig. 4

Source data Fig. 5

Source data Fig. 6

Source data Fig. 7

Source data Fig. 8

Source data Fig. 9

Expanded View Figures

## Data Availability

The coordinates of the experimental celiacase structure determined in this study are retrievable from the Protein Data Bank at www.pdb.org under access code 9QR8. The proteomics datasets are retrievable from Zenodo (https://zenodo.org/records/19456489). The source data of this paper are collected in the following database record: biostudies:S-SCDT-10_1038-S44321-026-00430-8.
