# Supplementary material for: Targeted enzymatic therapy for coeliac disease
Source: EMBO Mol Med. 2026 May 14;18(6):2236–71. doi: 10.1038/s44321-026-00430-8 (PMC13269764; doi:10.1038/s44321-026-00430-8)
Supplement: Supplementary file 1 — Appendix [file 44321_2026_430_MOESM1_ESM.pdf]

Appendix
for
Targeted enzymatic therapy for coeliac disease
by
Marina Girbal-González, Arturo Rodríguez-Banqueri, Hadeel Swaid, Soraia R. Mendes, Laura Garzón-Flores, Juan Sebastián Ramírez-Larrota, Carolina Cueva, M. Victoria Moreno-Arribas, Christof Regl, Christian Huber, Katharina A. Scherf, María José Rodríguez-Lagunas, Àngels Franch-Masferrer, Ulrich Eckhard, Francisco J. Pérez-Cano and F. Xavier Gomis-Rüth

Table of Contents **1**

1. Appendix figures

Appendix Figure S1 **2**
Appendix Figure S2 **3**
Appendix Figure S3 **4**
Appendix Figure S4 **5**
Appendix Figure S5 **6**
Appendix Figure S6 **7**
Appendix Figure S7 **8**
Appendix Figure S8 **9-11**
Appendix Figure S9 **12**
Appendix Figure S10 **13-14**
Appendix Figure S11 **15**
Appendix Figure S12 **16**

2. Appendix tables

Appendix Table S1 **17**
Appendix Table S2 **18**
Appendix Table S3 **19**
Appendix Table S4 **20**

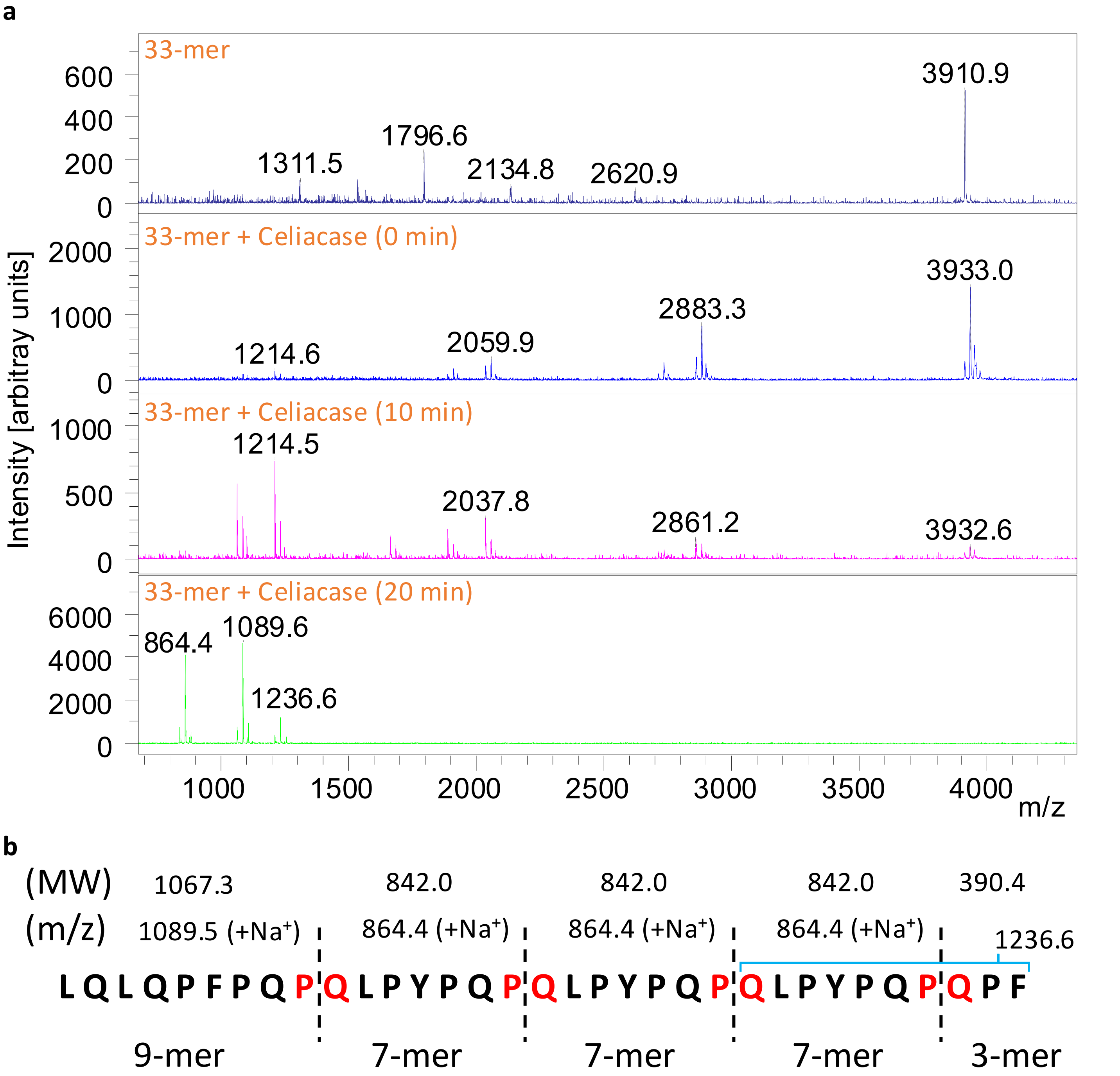

Appendix Figure S1 – *In vitro* cleavage of the 33-mer peptide. **a,** Time-course analysis of the enzymatic cleavage of the 33-mer (MW: 3,911 Da) by celiacase, monitored using an AutoFLEX III MALDI-TOF mass spectrometer. **b,** Cleavage sites (QP–Q/PL) identified from the MALDI-TOF data, revealing the final fragments “9-mer”, “7-mer” (present at threefold molar concentration), and “3-mer.”

a

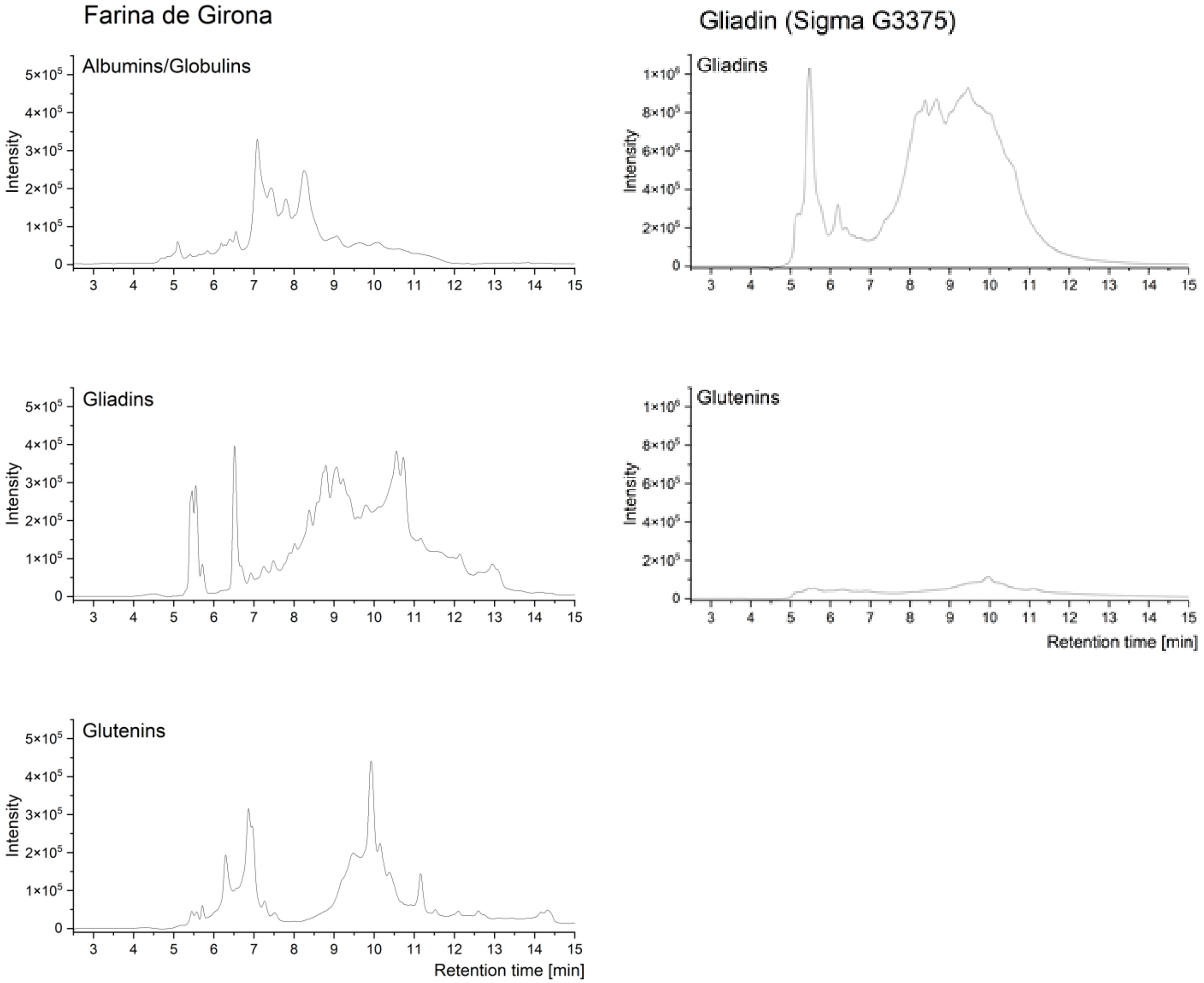

b

	Albumin/Globulin [mg/g]	Gliadin [mg/g]	Glutenin [mg/g]	Gluten [mg/g]	Total (HPLC) [mg/g]	Glia/Glut (ratio)
Farina de Girona	20.52	49.83	27.63	77.46	97.98	1.80
**Gliadin**	-	764.75	82.66	847.41	847.41	9.25

	ω5-Gliadin [mg/g]	ω1,2-Gliadin [mg/g]	α-Gliadin [mg/g]	γ-Gliadin [mg/g]	ωb-Gliadin [mg/g]	HMW-GS [mg/g]	LMW-GS [mg/g]
Farina de Girona	3.22	4.63	18.29	23.69	0.69	7.58	19.36
**Gliadin**	89.82	73.60	211.68	389.64	7.59	14.02	61.05

Appendix Figure S2 – Chromatographic profiles and protein-fraction analysis of wheat flour and gliadin. (A) Left panels display reversed-phase HPLC ultraviolet chromatograms of three protein fractions extracted from *Farina de Girona* wheat flour: albumins/globulins, gliadins, and glutenins (top to bottom). Right panels show the corresponding chromatograms of the gliadins and glutenins from a commercial gliadin standard (Sigma-Aldrich Cat. No. G3375). **(B)** Quantitative protein composition, expressed in milligrams per gram of sample [mg/g], broken down by albumins/globulins, gliadins, glutenins, gluten (sum of gliadins and gluteninn), and total protein content as determined by HPLC, together with the calculated gliadin/glutenin ratio. The lower section provides a detailed breakdown of specific gliadin types (ω5-, ω1,2-, α-, γ-, and ωb-gliadins) and high- and low-molecular-weight glutenin subunits (HMW–GS and LMW–GS). Note that the albumin/globulin content was not determined for the gliadin standard.

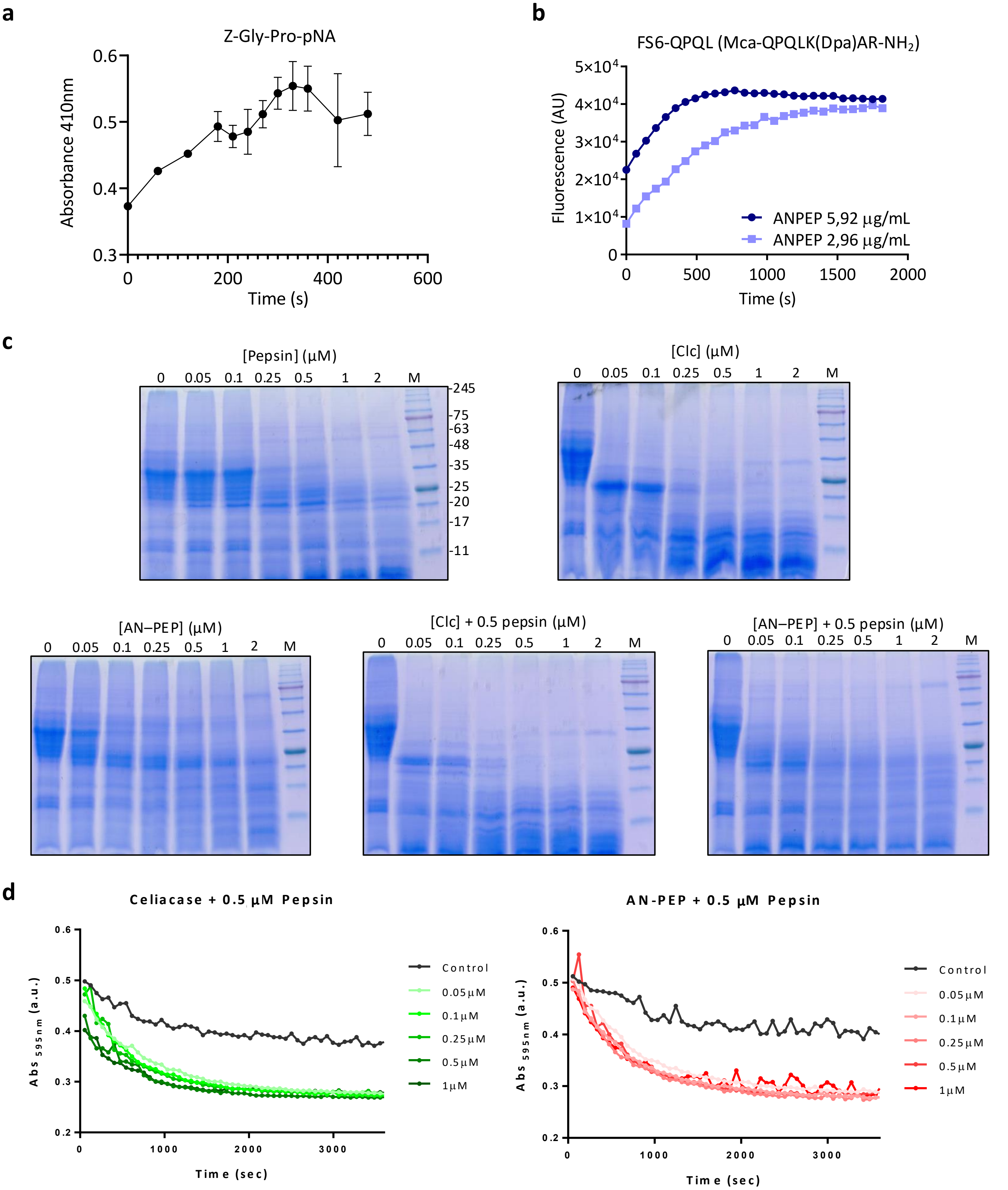

Appendix Figure S3 – Activity of AN-PEP and comparative digestion of gliadin. a, Activity of AN-PEP against Z-Gly-Pro-pNA (1 mM), the absorbance was measured at 410 nm (12 min; 37 ºC). Data are presented as mean ± SEM. **b**, Activity of AN-PEP against the FS6-QPQL fluorescent substrate (10µM) (30 min; 37 ºC). **c,** SDS-PAGE of commercial gliadin digestion by pepsin, celiacase (Clc), or AN–PEP at concentrations of 0 µM (control), 0.05 µM, 0.1 µM , 0.25 µM, 0.5 µM, 1 µM, and 2 µM. Digestions (2 h; 37 °C) were performed with/without additional 0.5 µM pepsin. Panels were reused from Figs. 1q and 2d). **d,** Turbidimetric assay of gliadin degradation by Clc (left) and AN–PEP (right) at the same concentrations, in the presence of 0.5 µM pepsin. Degradation was monitored measuring absorbance at 595 nm at 37 °C.

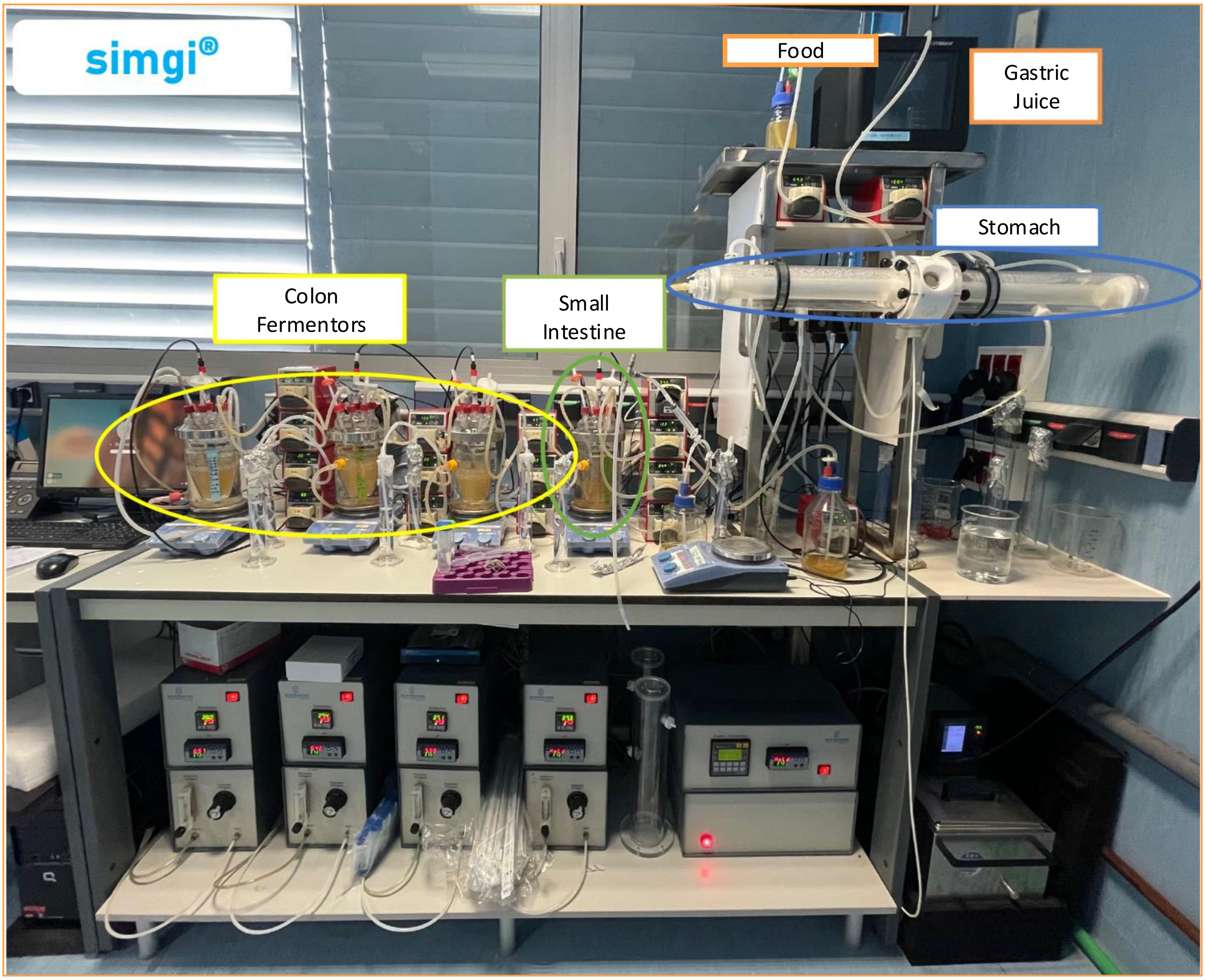

Appendix Figure S4 – Dynamic gastrointestinal simulator. Picture depicting the setup of the "Simgi®" dynamic gastrointestinal simulator at the Institute of Food Science Research (CIAL), CSIC-UAM, in Madrid (Spain) (https://cial.uam-csic.es/simgi/index_eng.html).

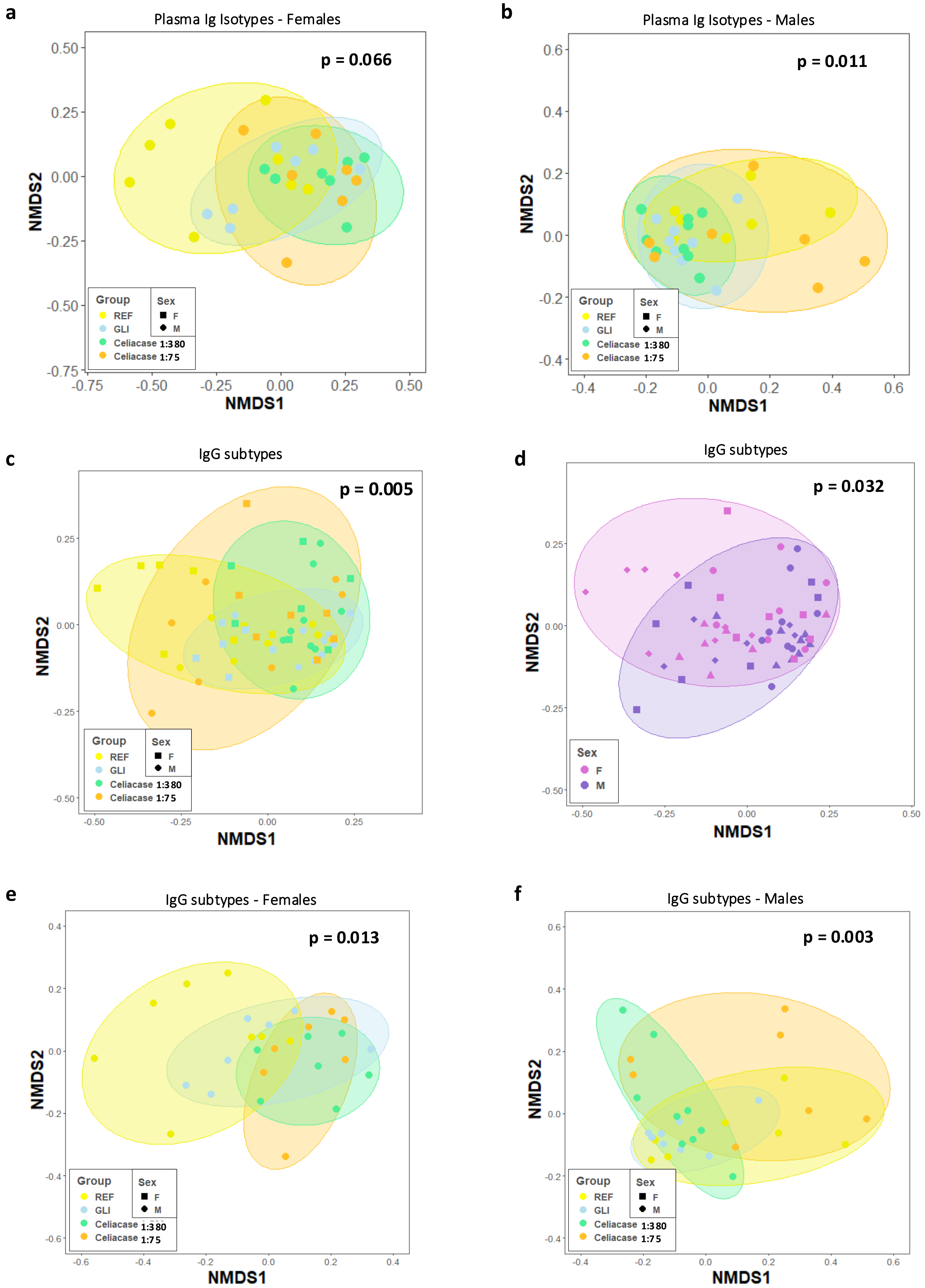

Appendix Figure S5 – Immunoglobulin analysis of DQ8-D^{d}-villin-IL-15tg mice after ED2. a–f, Non-metric multidimensional scaling (NMDS) analysis (see Section 4.20) for immunoglobulin (Ig) isotypes and IgG subtypes (IgG3, IgG2b, IgG2a, and IgG1) present in plasma of the REF, GLI, Clc 1:380, and Clc 1:75 groups. Data were analysed separately by groups and females (a,e) or males (b,f), as well as by groups (c) or by sex (d). The respective *p*-values are indicated.

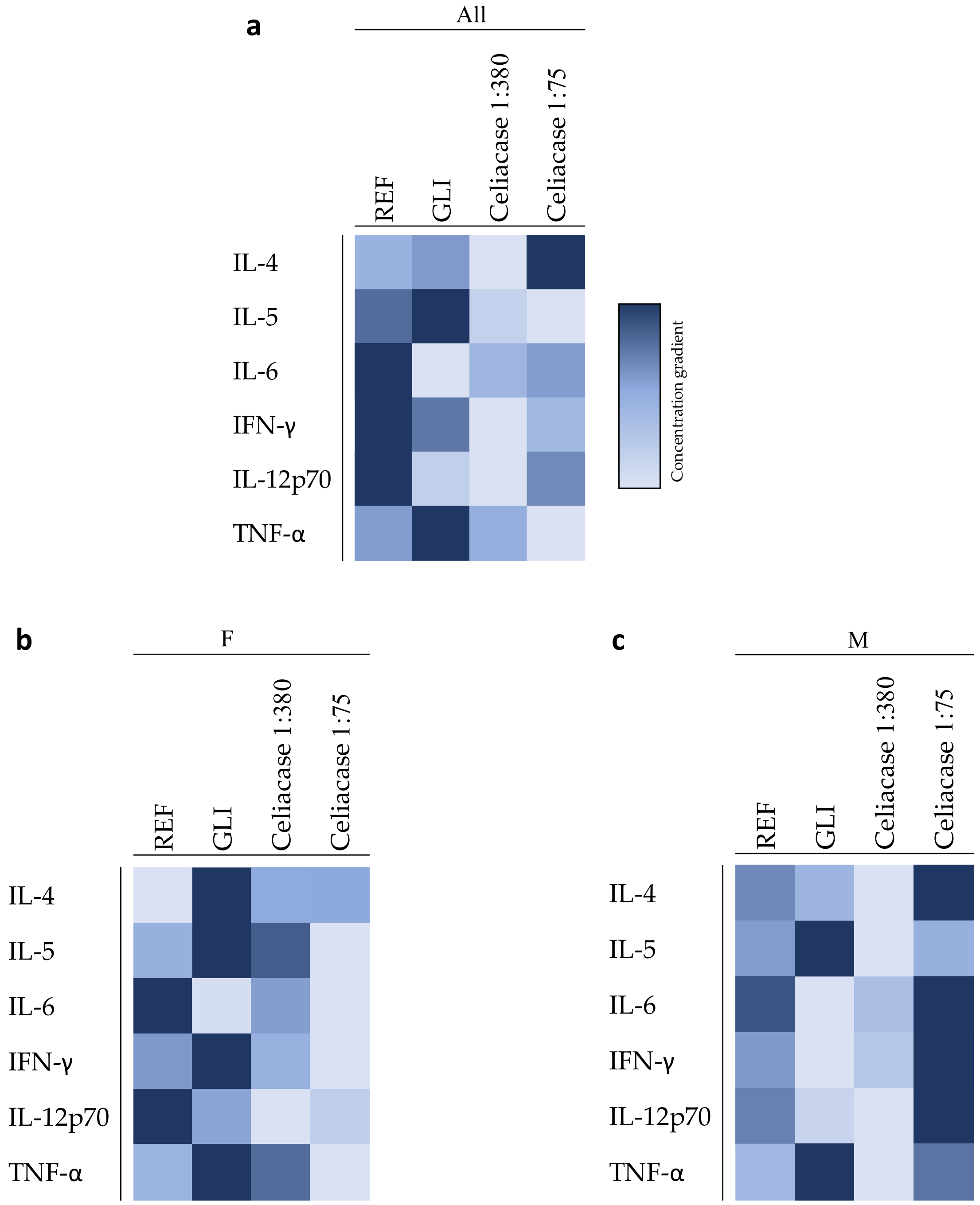

Appendix Figure S6 – Heatmap analysis of plasma cytokine levels of DQ8-D^{d}-villin-IL-15tg mice after ED2. a–c, Heatmaps depicting cytokine concentrations in plasma samples of the REF, GLI, Clc 1:380, and Clc 1:75 groups. The data were analysed for females (F), males (M), and combined (all). Colour intensity ranges from dark blue (high abundance) to light blue (low abundance), standardized according to the scale shown in (a). Abbreviations: IL, interleukin; IFN, interferon; TNF, tumour necrosis factor.

Phylum

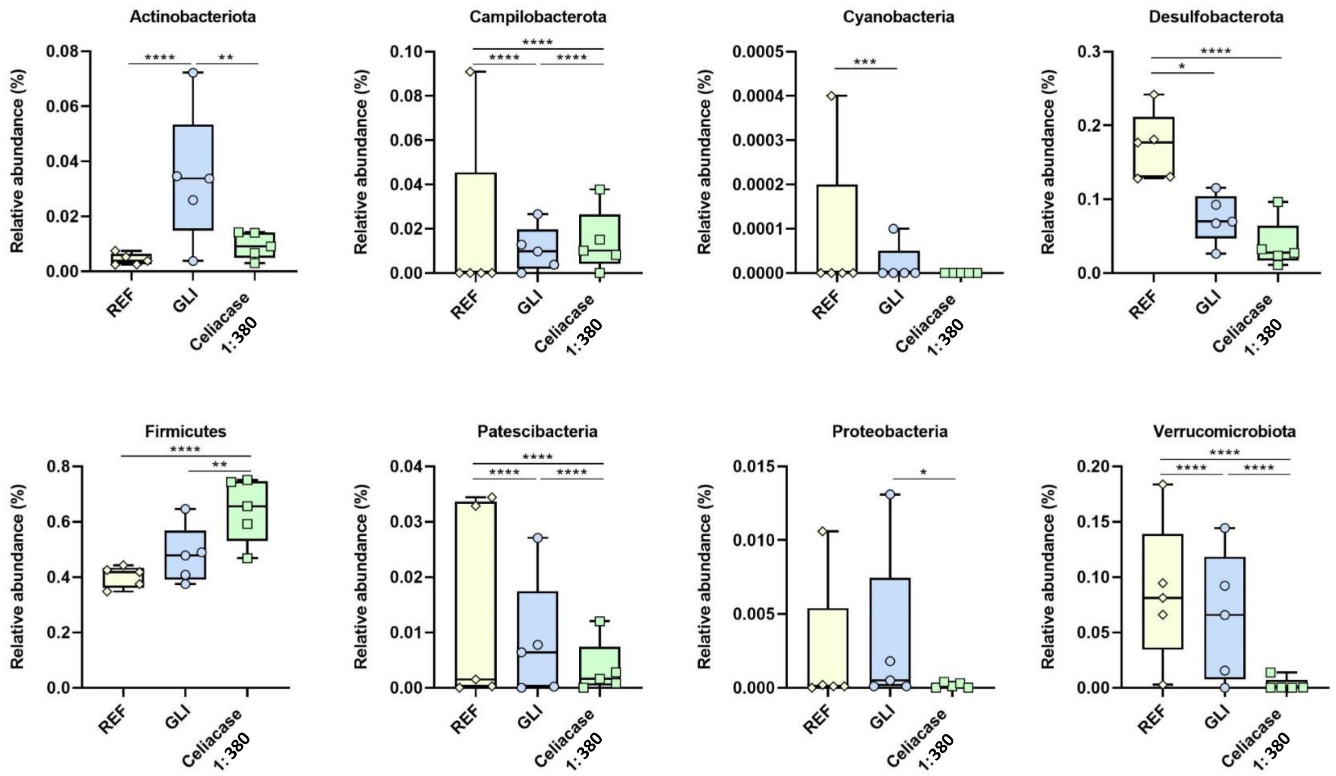

Appendix Figure S7 – Microbiota analysis by phylum of DQ8-D^d-villin-IL-15tg mice after ED2. Relative abundance of bacterial phyla in the caecal microbiota of the REF, GLI, and Clc 1:380 groups, determined via 16S rRNA sequencing. Only phyla showing significant differences between the treatment groups are included. Boxplots represent the median (horizontal line), interquartile range (box), and whiskers extending to 1.5-times the interquartile range. Outliers are shown as individual points. Statistical analyses and significance thresholds are as described in Section 4.20.

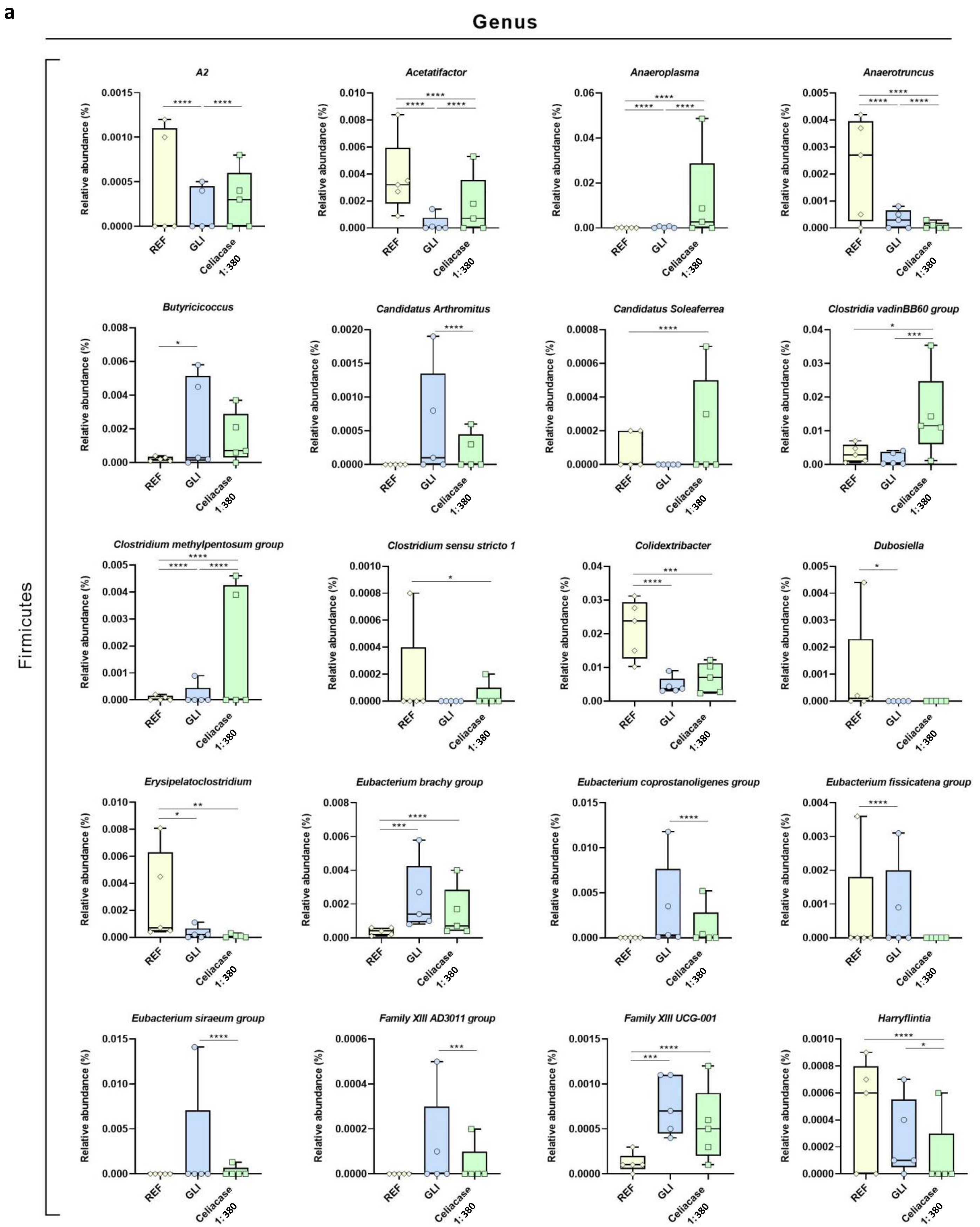
a
Genus
Firmicutes
A2
Acetatifactor
Anaeroplasma
Anaerotruncus
Butyricicoccus
Candidatus Arthromitus
Candidatus Soleaferrea
Clostridia vadinBB60 group
Clostridium methylpentosum group
Clostridium sensu stricto 1
Colidextribacter
Dubosiella
Erysipelatoclostridium
Eubacterium brachy group
Eubacterium coprostanoligenes group
Eubacterium fissicatena group
Eubacterium siraeum group
Family XIII AD3011 group
Family XIII UCG-001
Harryflintia
Relative abundance (%)
REF
GLI
Celiacase 1:380

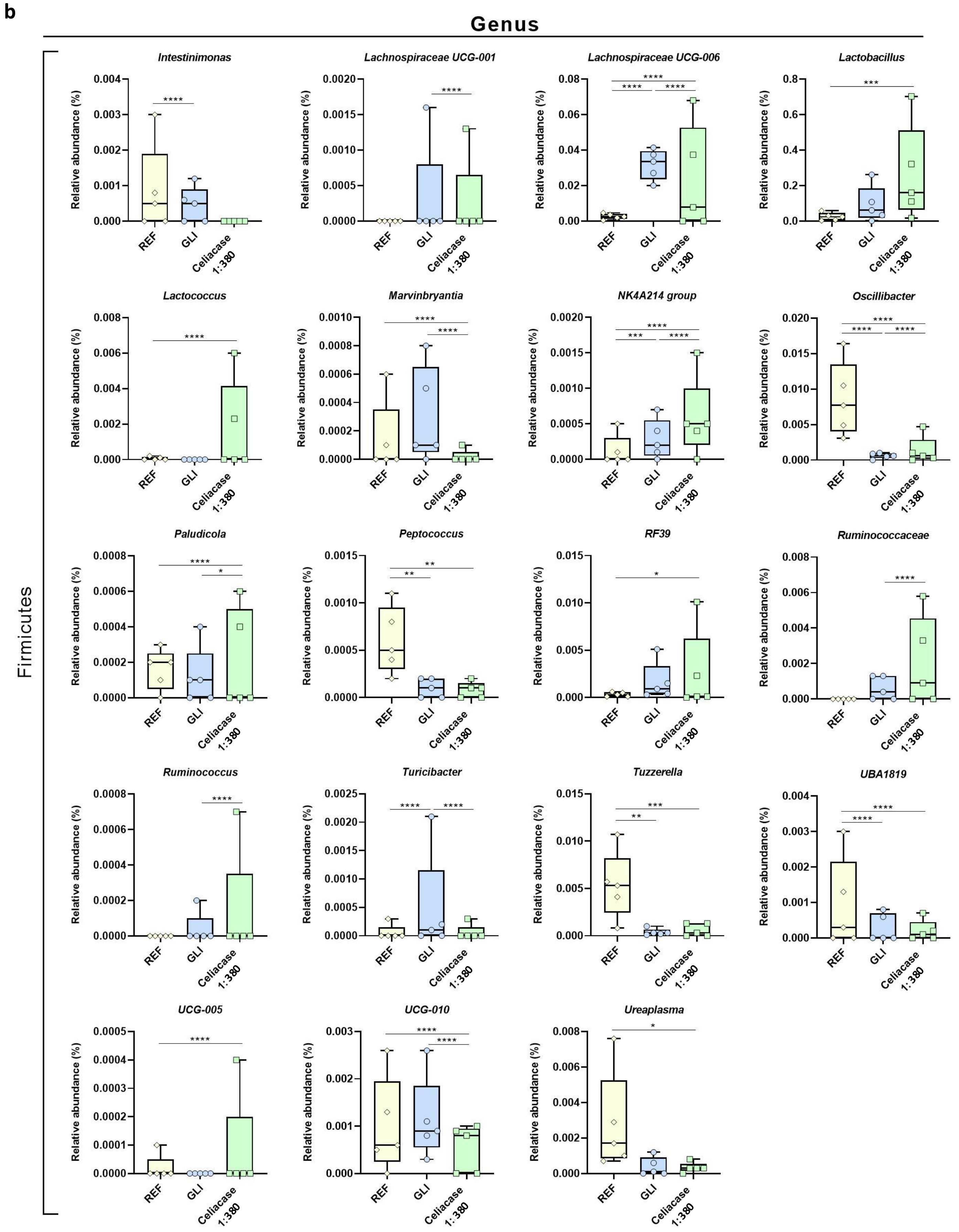
b
Genus
Firmicutes
Intestinimonas
Lachnospiraceae UCG-001
Lachnospiraceae UCG-006
Lactobacillus
Lactococcus
Marvinbryantia
NK4A214 group
Oscillibacter
Paludicola
Peptococcus
RF39
Ruminococcaceae
Ruminococcus
Turicibacter
Tuzzerella
UBA1819
UCG-005
UCG-010
Ureaplasma
Relative abundance (%)
REF
GLI
Celiacase 1:380

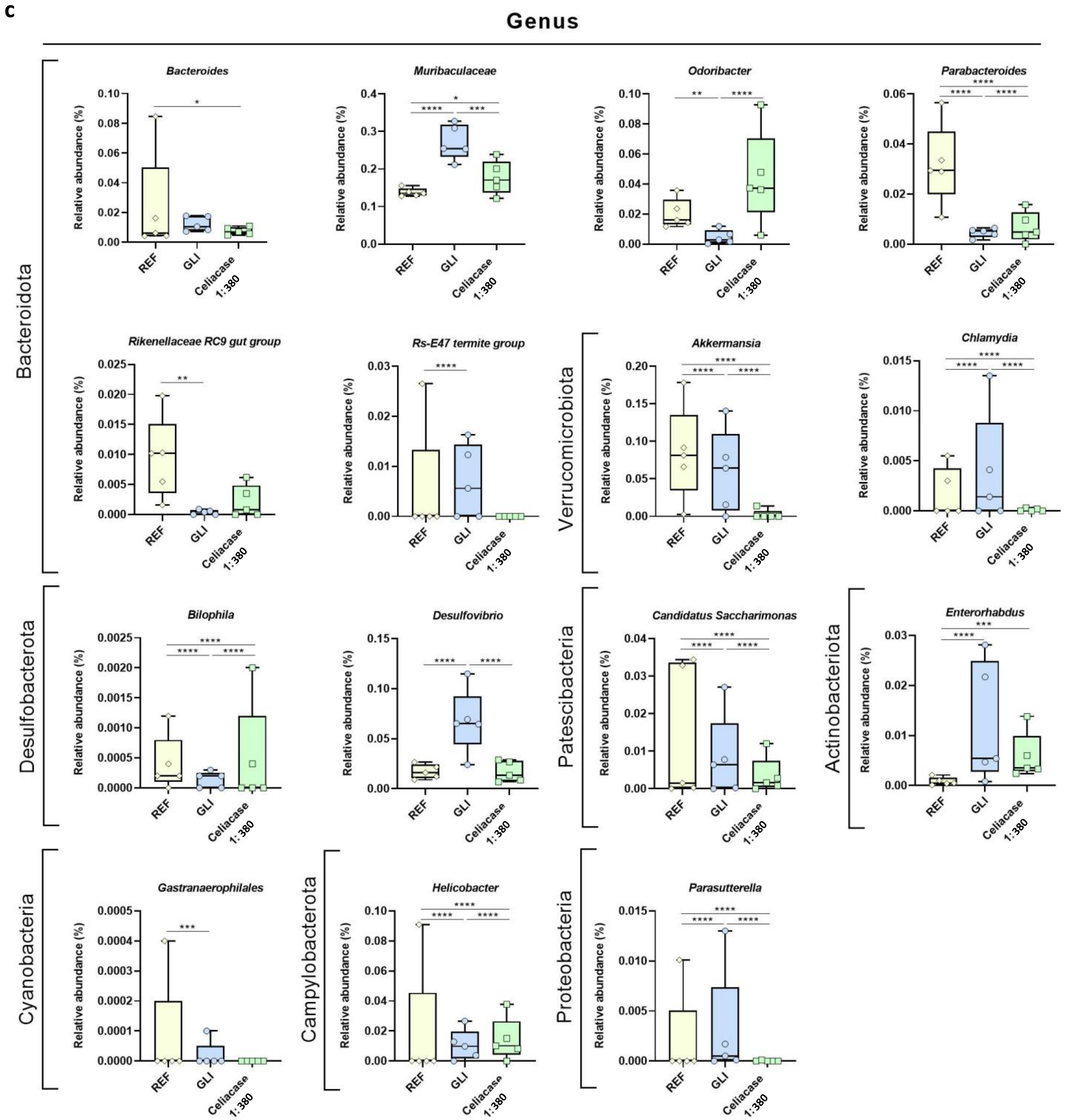

Appendix Figure S8 – Microbiota analysis by genus of DQ8-D^{d}-villin-IL-15tg mice after ED2. a–c, Relative abundance of bacterial genera in the caecal microbiota of the REF, GLI, and Clc 1:380 groups, determined via 16S rRNA sequencing. Only genera showing significant differences between the treatment groups are included. Boxplots represent the median (horizontal line), interquartile range (box), and whiskers extending to 1.5-times the interquartile range. Outliers are shown as individual points. Statistical analyses and significance thresholds are as described in Section 4.20.

Species

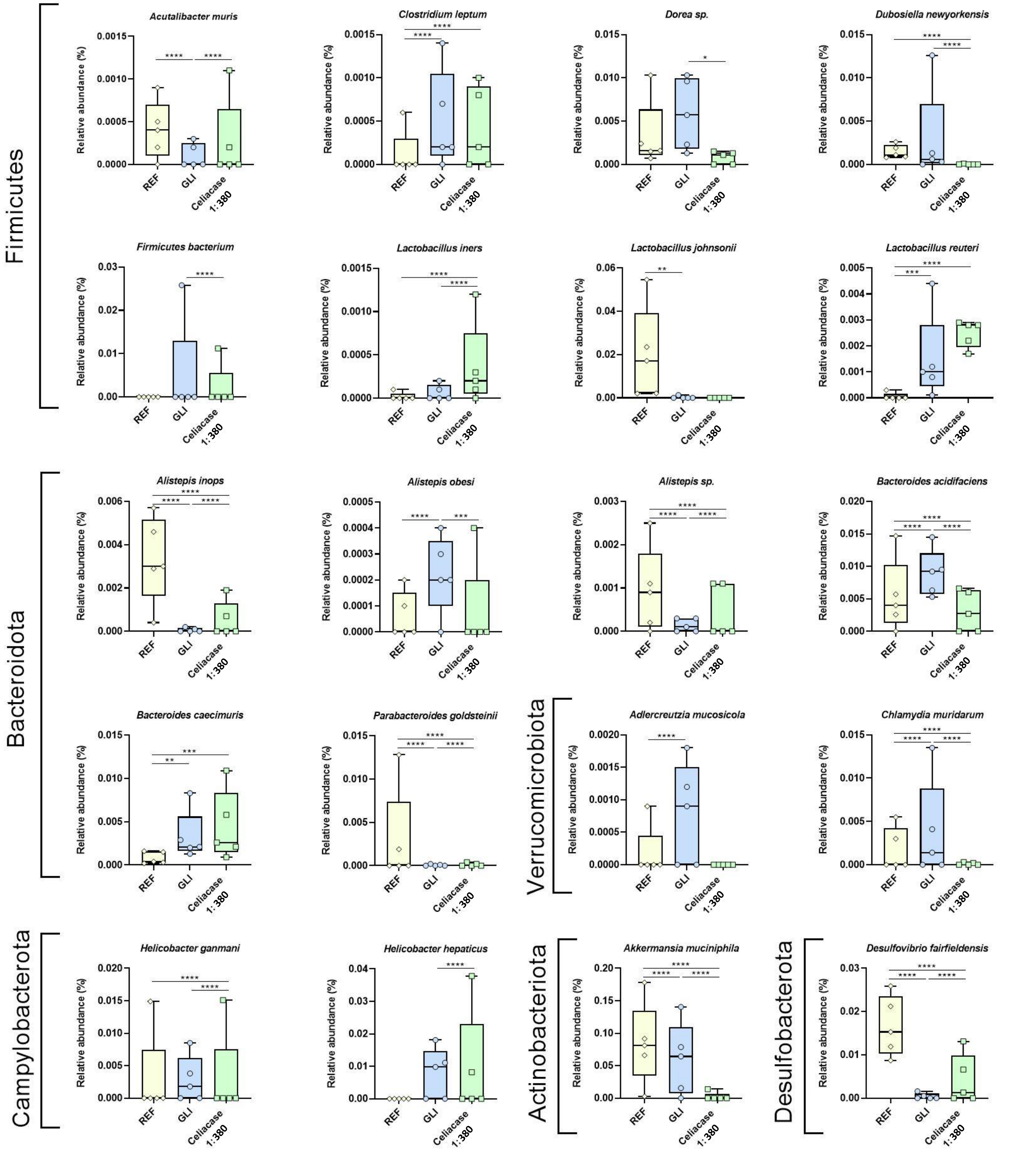

Appendix Figure S9 – Microbiota analysis by species of DQ8-D^{d}-villin-IL-15tg mice after ED2. Relative abundance of bacterial species, organized by phylum, in the caecal microbiota of the REF, GLI, and Clc 1:380 groups, determined via 16S rRNA sequencing. Only species showing significant differences between the treatment groups are included. Boxplots represent the median (horizontal line), interquartile range (box), and whiskers extending to 1.5-times the interquartile range. Outliers are shown as individual points. Statistical analyses and significance thresholds are as described in Section 4.20.

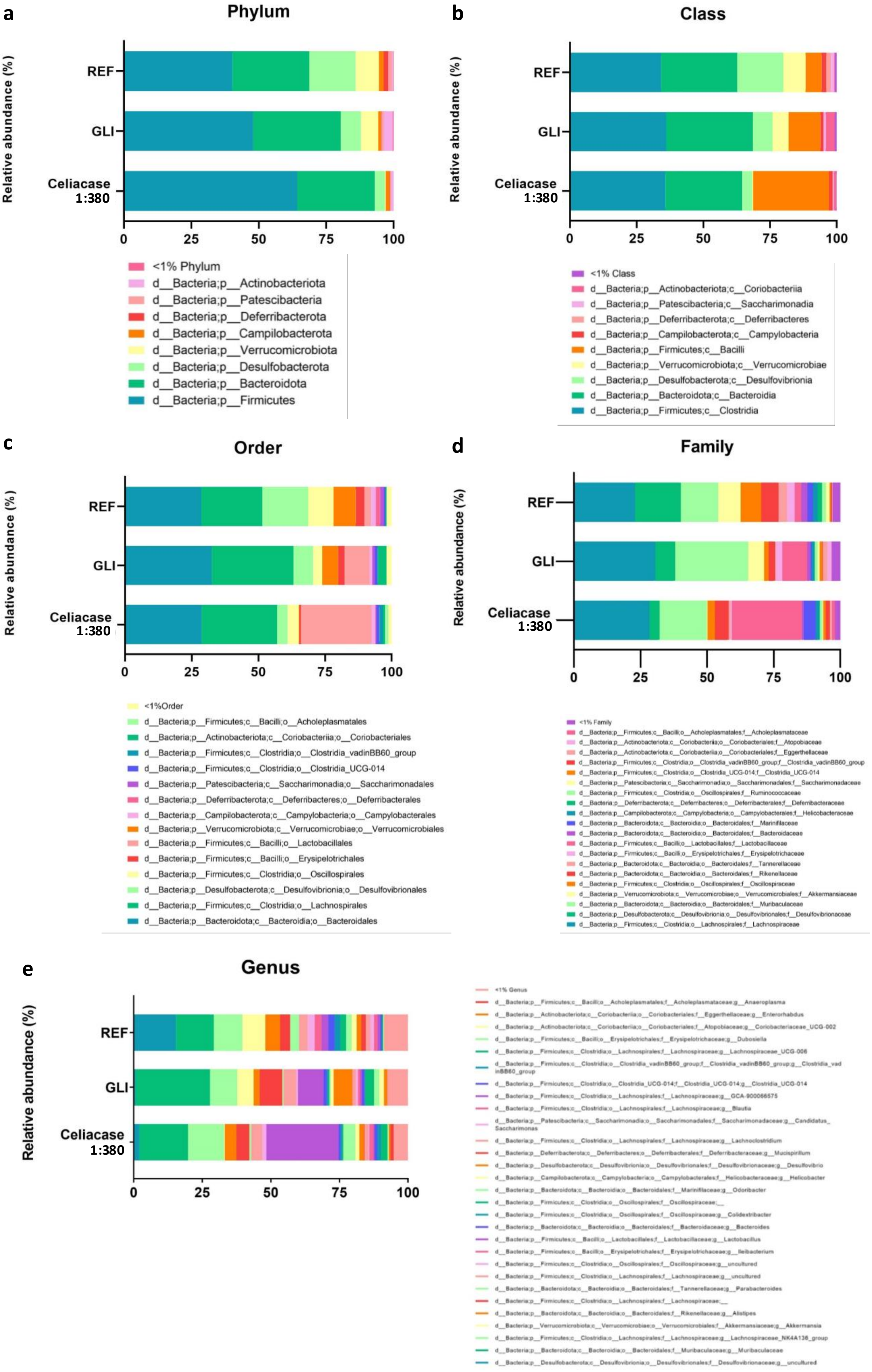
a
Phylum
Relative abundance (%)
REF
GLI
Celiacase 1:380
0
25
50
75
100
<1% Phylum
d__Bacteria;p__Actinobacteriota
d__Bacteria;p__Patescibacteria
d__Bacteria;p__Deferribacterota
d__Bacteria;p__Campilobacterota
d__Bacteria;p__Verrucomicrobiota
d__Bacteria;p__Desulfobacterota
d__Bacteria;p__Bacteroidota
d__Bacteria;p__Firmicutes
b
Class
Relative abundance (%)
REF
GLI
Celiacase 1:380
0
25
50
75
100
<1% Class
d__Bacteria;p__Actinobacteriota;c__Coriobacteriia
d__Bacteria;p__Patescibacteria;c__Saccharimonadia
d__Bacteria;p__Deferribacterota;c__Deferribacteres
d__Bacteria;p__Campilobacterota;c__Campylobacteria
d__Bacteria;p__Firmicutes;c__Bacilli
d__Bacteria;p__Verrucomicrobiota;c__Verrucomicrobiae
d__Bacteria;p__Desulfobacterota;c__Desulfovibrionia
d__Bacteria;p__Bacteroidota;c__Bacteroidia
d__Bacteria;p__Firmicutes;c__Clostridia
c
Order
Relative abundance (%)
REF
GLI
Celiacase 1:380
0
25
50
75
100
<1%Order
d__Bacteria;p__Firmicutes;c__Bacilli;o__Acholeplasmatales
d__Bacteria;p__Actinobacteriota;c__Coriobacteriia;o__Coriobacteriales
d__Bacteria;p__Firmicutes;c__Clostridia;o__Clostridia_vadinBB60_group
d__Bacteria;p__Firmicutes;c__Clostridia;o__Clostridia_UCG-014
d__Bacteria;p__Patescibacteria;c__Saccharimonadia;o__Saccharimonadales
d__Bacteria;p__Deferribacterota;c__Deferribacteres;o__Deferribacterales
d__Bacteria;p__Campilobacterota;c__Campylobacteria;o__Campylobacterales
d__Bacteria;p__Verrucomicrobiota;c__Verrucomicrobiae;o__Verrucomicrobiales
d__Bacteria;p__Firmicutes;c__Bacilli;o__Lactobacillales
d__Bacteria;p__Firmicutes;c__Bacilli;o__Erysipelotrichales
d__Bacteria;p__Firmicutes;c__Clostridia;o__Oscillospirales
d__Bacteria;p__Desulfobacterota;c__Desulfovibrionia;o__Desulfovibrionales
d__Bacteria;p__Firmicutes;c__Clostridia;o__Lachnospirales
d__Bacteria;p__Bacteroidota;c__Bacteroidia;o__Bacteroidales
d
Family
Relative abundance (%)
REF
GLI
Celiacase 1:380
0
25
50
75
100
<1% Family
d__Bacteria;p__Firmicutes;c__Bacilli;o__Acholeplasmatales;f__Acholeplasmataceae
d__Bacteria;p__Actinobacteriota;c__Coriobacteriia;o__Coriobacteriales;f__Atopobiaceae
d__Bacteria;p__Actinobacteriota;c__Coriobacteriia;o__Coriobacteriales;f__Eggerthellaceae
d__Bacteria;p__Firmicutes;c__Clostridia;o__Clostridia_vadinBB60_group;f__Clostridia_vadinBB60_group
d__Bacteria;p__Firmicutes;c__Clostridia;o__Clostridia_UCG-014;f__Clostridia_UCG-014
d__Bacteria;p__Patescibacteria;c__Saccharimonadia;o__Saccharimonadales;f__Saccharimonadaceae
d__Bacteria;p__Firmicutes;c__Clostridia;o__Oscillospirales;f__Ruminococcaceae
d__Bacteria;p__Deferribacterota;c__Deferribacteres;o__Deferribacterales;f__Deferribacteraceae
d__Bacteria;p__Campilobacterota;c__Campylobacteria;o__Campylobacterales;f__Helicobacteraceae
d__Bacteria;p__Bacteroidota;c__Bacteroidia;o__Bacteroidales;f__Marinifilaceae
d__Bacteria;p__Bacteroidota;c__Bacteroidia;o__Bacteroidales;f__Bacteroidaceae
d__Bacteria;p__Firmicutes;c__Bacilli;o__Lactobacillales;f__Lactobacillaceae
d__Bacteria;p__Firmicutes;c__Bacilli;o__Erysipelotrichales;f__Erysipelotrichaceae
d__Bacteria;p__Bacteroidota;c__Bacteroidia;o__Bacteroidales;f__Tannerellaceae
d__Bacteria;p__Bacteroidota;c__Bacteroidia;o__Bacteroidales;f__Rikenellaceae
d__Bacteria;p__Firmicutes;c__Clostridia;o__Oscillospirales;f__Oscillospiraceae
d__Bacteria;p__Verrucomicrobiota;c__Verrucomicrobiae;o__Verrucomicrobiales;f__Akkermansiaceae
d__Bacteria;p__Bacteroidota;c__Bacteroidia;o__Bacteroidales;f__Muribaculaceae
d__Bacteria;p__Desulfobacterota;c__Desulfovibrionia;o__Desulfovibrionales;f__Desulfovibrionaceae
d__Bacteria;p__Firmicutes;c__Clostridia;o__Lachnospirales;f__Lachnospiraceae
e
Genus
Relative abundance (%)
REF
GLI
Celiacase 1:380
0
25
50
75
100
<1% Genus
d__Bacteria;p__Firmicutes;c__Bacilli;o__Acholeplasmatales;f__Acholeplasmataceae;g__Anaeroplasma
d__Bacteria;p__Actinobacteriota;c__Coriobacteriia;o__Coriobacteriales;f__Eggerthellaceae;g__Enterorhabdus
d__Bacteria;p__Actinobacteriota;c__Coriobacteriia;o__Coriobacteriales;f__Atopobiaceae;g__Coriobacteriaceae_UCG-002
d__Bacteria;p__Firmicutes;c__Bacilli;o__Erysipelotrichales;f__Erysipelotrichaceae;g__Dubosiella
d__Bacteria;p__Firmicutes;c__Clostridia;o__Lachnospirales;f__Lachnospiraceae;g__Lachnospiraceae_UCG-006
d__Bacteria;p__Firmicutes;c__Clostridia;o__Clostridia_vadinBB60_group;f__Clostridia_vadinBB60_group;g__Clostridia_vadinBB60_group
d__Bacteria;p__Firmicutes;c__Clostridia;o__Clostridia_UCG-014;f__Clostridia_UCG-014;g__Clostridia_UCG-014
d__Bacteria;p__Firmicutes;c__Clostridia;o__Lachnospirales;f__Lachnospiraceae;g__GCA-900066575
d__Bacteria;p__Firmicutes;c__Clostridia;o__Lachnospirales;f__Lachnospiraceae;g__Blautia
d__Bacteria;p__Patescibacteria;c__Saccharimonadia;o__Saccharimonadales;f__Saccharimonadaceae;g__Candidatus_Saccharimonas
d__Bacteria;p__Firmicutes;c__Clostridia;o__Lachnospirales;f__Lachnospiraceae;g__Lachnoclostridium
d__Bacteria;p__Deferribacterota;c__Deferribacteres;o__Deferribacterales;f__Deferribacteraceae;g__Mucispirillum
d__Bacteria;p__Desulfobacterota;c__Desulfovibrionia;o__Desulfovibrionales;f__Desulfovibrionaceae;g__Desulfovibrio
d__Bacteria;p__Campilobacterota;c__Campylobacteria;o__Campylobacterales;f__Helicobacteraceae;g__Helicobacter
d__Bacteria;p__Bacteroidota;c__Bacteroidia;o__Bacteroidales;f__Marinifilaceae;g__Odoribacter
d__Bacteria;p__Firmicutes;c__Clostridia;o__Oscillospirales;f__Oscillospiraceae;__
d__Bacteria;p__Firmicutes;c__Clostridia;o__Oscillospirales;f__Oscillospiraceae;g__Colidextribacter
d__Bacteria;p__Bacteroidota;c__Bacteroidia;o__Bacteroidales;f__Bacteroidaceae;g__Bacteroides
d__Bacteria;p__Firmicutes;c__Bacilli;o__Lactobacillales;f__Lactobacillaceae;g__Lactobacillus
d__Bacteria;p__Firmicutes;c__Bacilli;o__Erysipelotrichales;f__Erysipelotrichaceae;g__Ileibacterium
d__Bacteria;p__Firmicutes;c__Clostridia;o__Oscillospirales;f__Oscillospiraceae;g__uncultured
d__Bacteria;p__Firmicutes;c__Clostridia;o__Lachnospirales;f__Lachnospiraceae;g__uncultured
d__Bacteria;p__Bacteroidota;c__Bacteroidia;o__Bacteroidales;f__Tannerellaceae;g__Parabacteroides
d__Bacteria;p__Firmicutes;c__Clostridia;o__Lachnospirales;f__Lachnospiraceae;__
d__Bacteria;p__Bacteroidota;c__Bacteroidia;o__Bacteroidales;f__Rikenellaceae;g__Alistipes
d__Bacteria;p__Verrucomicrobiota;c__Verrucomicrobiae;o__Verrucomicrobiales;f__Akkermansiaceae;g__Akkermansia
d__Bacteria;p__Firmicutes;c__Clostridia;o__Lachnospirales;f__Lachnospiraceae;g__Lachnospiraceae_NK4A136_group
d__Bacteria;p__Bacteroidota;c__Bacteroidia;o__Bacteroidales;f__Muribaculaceae;g__Muribaculaceae
d__Bacteria;p__Desulfobacterota;c__Desulfovibrionia;o__Desulfovibrionales;f__Desulfovibrionaceae;g__uncultured

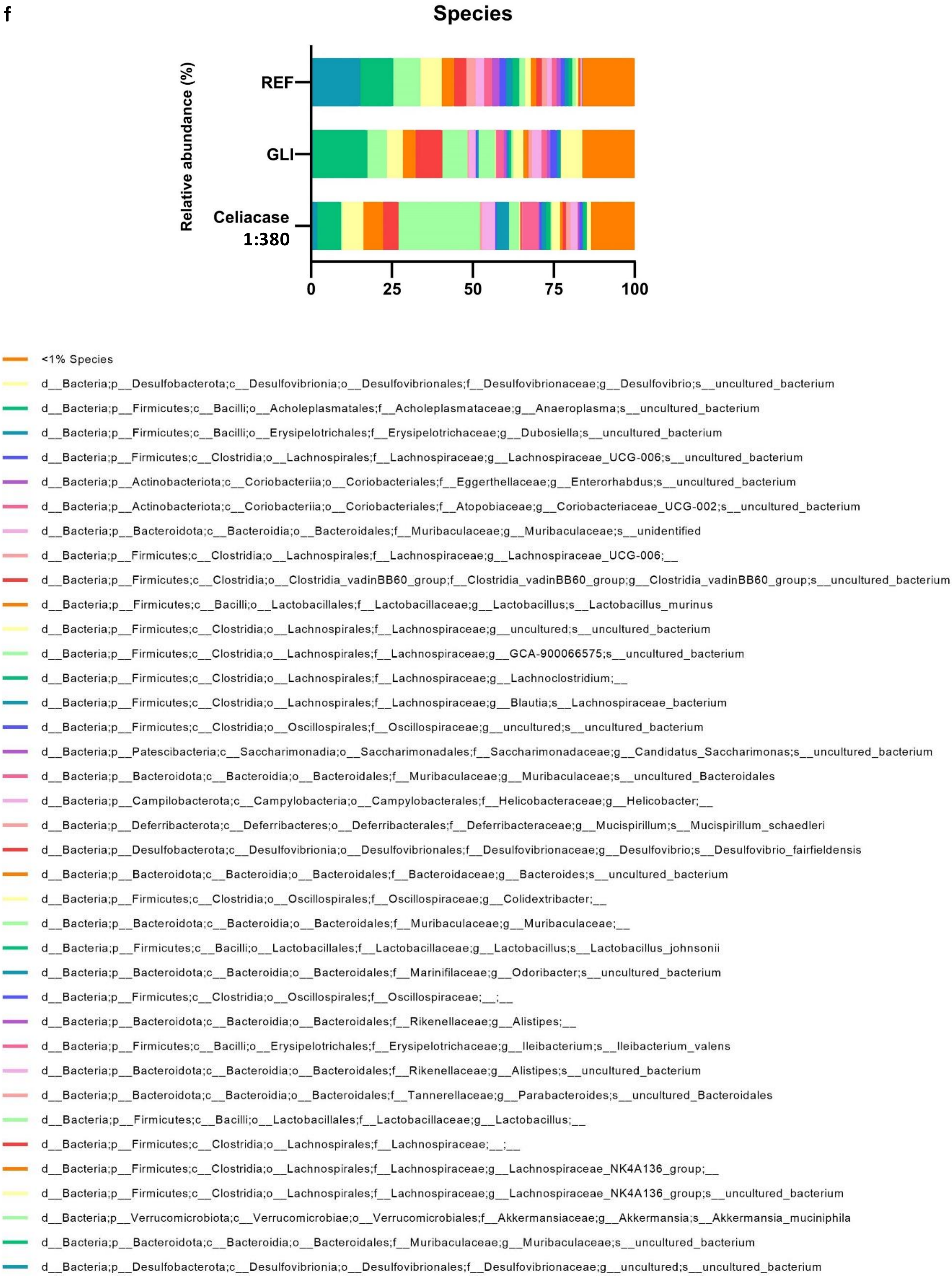

Appendix Figure S10 – Comprehensive microbiota analysis of DQ8-D^d-villin-IL-15tg mice after ED2. a–f, Bar plots showing the relative abundance in the caecal microbiota of the REF, GLI, and Clc 1:380 groups of bacterial taxa at the phylum (a), class (b), order (c), family (d), genus (e), and species (f) levels, determined by 16S rRNA sequencing.

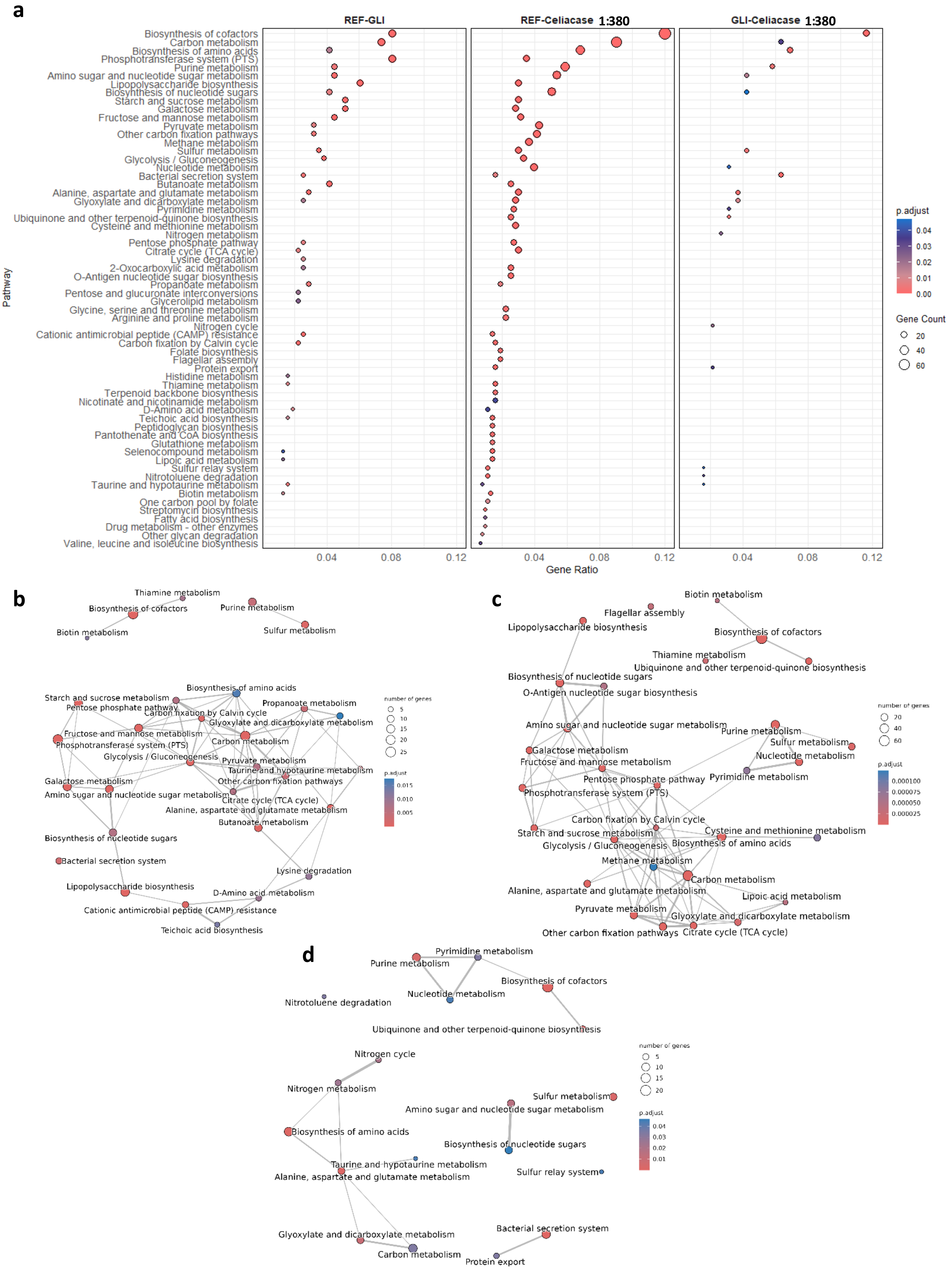

Appendix Figure S11 – Functional profiling of the caecal microbiota of DQ8-D^{d}-villin-IL-15tg mice after ED2. Functional predictions of the caecal microbiota were inferred for the REF, GLI, and Clc 1:380 groups (n = 5 per group) according to Section 4.19, based on the 30 most significantly enriched pathways. **a,** Overrepresented pathways in pairwise comparisons: REF–GLI, REF–Clc 1:380, and GLI–Clc 1:380. **b–d,** Enrichment maps for REF–GLI (b), REF–Clc 1:380 (c), and GLI–Clc 1:380 (d). Edges connect pathways sharing ≥5% similarity, scaled by shared gene count. The circle size reflects the number of overrepresented gene orthologues, and colour indicates significance (blue: $p \leq 0.05$, red: $p \leq 0.001$).

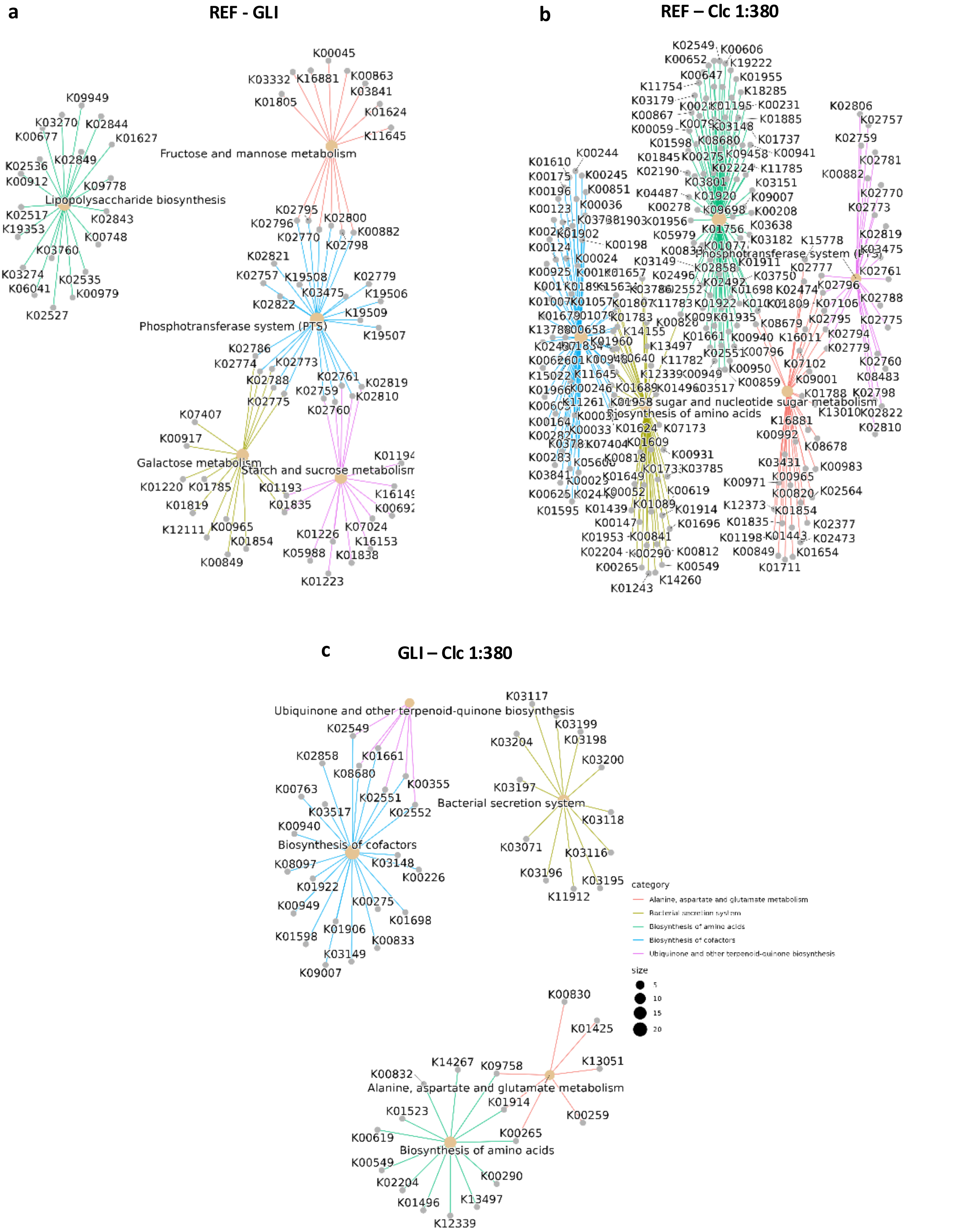

Appendix Figure S12 – Category netplots. a–c, Category netplots illustrating the linkages among the five most significant KEGG functions (see Section 4.19). The networks depict which functions are associated with each enriched pathway and highlight those shared by the three DQ8-D^{d}-villin-IL-15tg mouse groups—REF, GLI, and Clc 1:380—after ED2. Pairwise comparisons include REF vs. GLI, REF vs. Clc 1:380, and GLI vs. Clc 1:380.

Appendix Table S1 — Crystallographic data.

Specimen	Mature celiacase
Beam line (synchrotron)	ID23 (ESRF)
Detector	Eiger2 16M CdTe
Processing software	*Xds* / *Xscale*
Space group / protomers per a.u. [a]	$P2_1$ / 1 (chain A)
Cell constants (a, b, c in Å; β in º)	59.01, 39.58, 64.64, 108.11
Wavelength (Å)	0.97919
No. of measurements / unique reflections	72,728 / 21,884
Resolution range (Å) [b]	61.4 – 1.90 (2.01 – 1.90)
Completeness (%)	96.3 (81.2)
R_{merge} [c]	0.158 (0.907)
R_{meas} [c]	0.186 (1.174)
CC($^1/_2$) [c]	0.992 (0.578)
Average intensity [d]	6.8 (1.0)
B-Factor (Wilson) ($Å^2$)	32.2
Aver. Multiplicity	3.3 (2.3)
Refinement software	*Phenix.Refine*
Resolution range (Å)	61.4 – 1.90
No. of reflections used in refinement [b]	21,336 [529]
Crystallographic R_{factor} / free R_{factor} [c]	0.210 / 0.258
F_{obs} , F_{calc} correlation	0.96
Residues with RSRZ [a] > 2	5 out of 256
No. of protein residues / all atoms / solvent molecules / ions /	256 / 2280 / 185 / 1 SO_4^{2-}, 2 Cl^- /
ions / non-covalent ligands [a]	1 TRS, 1 GOL, 1 PEG, 5 GLY
sugar moieties anchored to protein residues	N^{145}→NAG–FUC; N^{152}→NAG–NAG–BMA
Rmsd from target values for protein residues	
bonds / angles / chirality / planarity	0.007 / 0.902 / 0.057 / 0.008
Average B-factors ($Å^2$)	32.5
Protein residues with	
favoured Ramachandran regions / outliers / all residues	247 (97%) / 0 / 254
rotamer / bond / angle / chirality / planarity / Cβ outliers	5 (2%) / 0 / 0 / 0 / 0 / 0
All-atom clashes / clashscore	12 / 2.9
Molprobity score	1.5
Protein residues in multiple conformations	0
PDB access code	9QR8

[a] Abbreviations: a.u., asymmetric unit; BMA, β-D-mannose; FUC, α-L-fucose; GLY, glycine; GOL, glycerol; NAG, β-*N*-acetylglucosamine; PEG, diethylene glycol; TRS, tris(hydroxymethyl)aminomethane; RSRZ, real space R_{value} normalised against residue type and resolution (Fourier-map outliers). [b] In parentheses, values for the highest resolution shell; in brackets, values for the test set. [c] For definitions, see https://onlinelibrary.wiley.com/doi/abs/10.1107/97809553602060000809. [d] Average intensity is < I/σ(I) > of unique reflections after merging according to the *Xscale* program. Validation according to the *Phenix* program and the wwPDB Server.

Appendix Table S2 – Morphometric and weight measurements.

		Body weight d25 (g)	Organ weights (g)						Length (cm)		BMI Index (g/cm2)	Lee Index
			Stomach	Small Intestine	Cecum	Liver	Thymus	Spleen	Naso-anal	Total		
REF	All	22.06 ± 0.70	1.13 ± 0.11	5.00 ± 0.27	0.84 ± 0.05	4.88 ± 0.10	0.25 ± 0.02	0.46 ± 0.07	9.01 ± 0.12	16.66 ± 0.16	0.27 ± 0.01	311.55 ± 4.73
	F	19.90 ± 0.55	1.20 ± 0.14	5.18 ± 0.39	0.84 ± 0.03	5.03 ± 0.12	0.32 ± 0.01	0.56 ± 0.12	8.90 ± 0.13	16.49 ± 0.18	0.25 ± 0.01	304.75 ± 5.07
	M	24.53 ± 0.48	1.05 ± 0.16	4.80 ± 0.34	0.83 ± 0.10	4.70 ± 0.15	0.18 ± 0.01	0.35 ± 0.04	9.13 ± 0.21	16.86 ± 0.27	0.30 ± 0.01	319.31 ± 7.28
GLI	All	24.09 ± 0.82	0.83 ± 0.05*	5.18 ± 0.22	1.39 ± 0.08*	4.93 ± 0.17	0.23 ± 0.02	0.36 ± 0.04	8.95 ± 0.12	16.44 ± 0.15	0.30 ± 0.01	322.34 ± 3.38
	F	21.34 ± 0.75	0.94 ± 0.08	5.89 ± 0.25	1.54 ± 0.09	5.19 ± 0.28	0.31 ± 0.03	0.37 ± 0.03	8.61 ± 0.14	16.03 ± 0.20	0.29 ± 0.01	322.22 ± 5.68
	M	26.49 ± 0.62	0.74 ± 0.04	4.55 ± 0.15	1.27 ± 0.10	4.69 ± 0.16	0.17 ± 0.01	0.36 ± 0.07	9.25 ± 0.12	16.80 ± 0.13	0.31 ± 0.01	322.44 ± 3.92
Celiacase 1:380	All	23.36 ± 0.78	0.95 ± 0.04	5.24 ± 0.17	1.50 ± 0.10*	4.77 ± 0.18	0.29 ± 0.02	0.38 ± 0.03	8.74 ± 0.10	16.47 ± 0.15	0.31 ± 0.01	326.87 ± 3.54
	F	21.00 ± 0.76	0.99 ± 0.05	5.59 ± 0.26	1.54 ± 0.17	4.65 ± 0.35	0.37 ± 0.02	0.44 ± 0.04	8.50 ± 0.11	16.06 ± 0.15	0.29 ± 0.01	324.57 ± 5.17
	M	25.20 ± 0.85	0.93 ± 0.07	4.98 ± 0.19	1.46 ± 0.12	4.86 ± 0.15	0.22 ± 0.02	0.33 ± 0.02	8.92 ± 0.12	16.79 ± 0.17	0.32 ± 0.01	328.67 ± 4.76
Celiacase 1:75	All	23.14 ± 0.81	1.05 ± 0.08	5.35 ± 0.24	1.42 ± 0.10*	4.87 ± 0.17	0.27 ± 0.02	0.35 ± 0.03	8.79 ± 0.14	16.49 ± 0.18	0.30 ± 0.01	324.25 ± 4.79
	F	20.69 ± 0.30	1.03 ± 0.08	5.84 ± 0.24	1.48 ± 0.17	5.03 ± 0.20	0.30 ± 0.01	0.41 ± 0.05	8.61 ± 0.18	16.50 ± 0.22	0.28 ± 0.01	319.49 ± 6.00
	M	25.59 ± 0.90	1.07 ± 0.13	4.85 ± 0.32	1.35 ± 0.08	4.70 ± 0.27	0.25 ± 0.02#	0.29 ± 0.02	8.97 ± 0.19	16.49 ± 0.29	0.32 ± 0.02	329.02 ± 7.01

Morphometric, weight, body mass index (BMI), and Lee index measurements for the REF, GLI, Clc 1:380, and Clc 1:75 groups of DQ8-D^{d}-villin-IL-15tg mice (N = 14 per group, 7 females and 7 males). The statistical analyses are described in Section 4.20, and the statistical significance is * = $p \leq 0.05$ vs. REF.

Appendix Table S3 – Analysis of haematological variables.

	REF	GLI	Clc 1:380	Clc 1:75
**WBC (cells/L)**	2.43E+09 ± 3.79E+08	2.92E+09 ± 3.01E+08	2.62E+09 ± 4.12E+08	2.19E+09 ± 3.10E+08
**LYM (%)**	13.16 ± 1.14	12.18 ± 1.86	12.57 ± 0.98	11.51 ± 1.10
**MID (%)**	36.05 ± 1.81	33.91 ± 1.36	34.58 ± 1.45	35.29 ± 1.39
**GRAN (%)**	50.79 ± 1.44	50.91 ± 2.74	51.91 ± 1.73	52.48 ± 1.54
**LYM (cells/L)**	3.26E+08 ± 8.32E+07	2.84E+08 ± 5.78E+07	3.58E+08 ± 8.19E+07	1.97E+08 ± 3.56E+07
**MID (cells/L)**	7.73E+08 ± 1.11E+08	8.67E+08 ± 9.69E+07	9.00E+08 ± 1.43E+08	6.29E+08 ± 1.10E+08
**GRAN (cells/L)**	1.33E+09 ± 1.94E+08	1.66E+09 ± 1.79E+08	1.44E+09 ± 2.00E+08	1.37E+09 ± 1.91E+08
**RBC (cells/L)**	8.38E+12 ± 1.15E+11	8.45E+12 ± 1.36E+11	7.51E+12 ± 3.27E+11*#	8.07E+12 ± 2.80E+11
**Hb (g/dL)**	11.25 ± 0.16	11.37 ± 0.20	10.31 ± 0.42*#	10.85 ± 0.38
**Hct (%)**	40.93 ± 0.72	40.89 ± 0.71	36.68 ± 1.41*#	38.84 ± 1.40
**MCV (f/L)**	48.08 ± 0.22	48.43 ± 0.11	47.95 ± 0.22	48.16 ± 0.17
**MCH (pg)**	13.53 ± 0.21	13.41 ± 0.08	13.49 ± 0.20	13.64 ± 0.20
**MCHC (g/dL)**	27.81 ± 0.27	27.77 ± 0.17	28.22 ± 0.36	28.03 ± 0.33
**RDW-CV (%)**	11.83 ± 0.16	12.31 ± 0.16	12.27 ± 0.14	12.04 ± 0.17
**RDW-SD (fL)**	19.93 ± 0.18	19.93 ± 0.16	19.70 ± 0.25	19.69 ± 0.19
**PLQ (cells/L)**	3.18E+11 ± 5.02E+10	4.09E+11 ± 3.80E+10	2.47E+11 ± 5.41E+10#	2.92E+11 ± 6.21E+10
**MPV (fL)**	8.41 ± 0.28	8.20 ± 0.50	8.90 ± 0.91	8.09 ± 0.30
**PDW (fL)**	8.60 ± 0.33	8.53 ± 0.19	8.39 ± 0.31	8.55 ± 0.32
**PCT (%)**	0.27 ± 0.04	0.31 ± 0.03	0.25 ± 0.04	0.24 ± 0.05
**P-LCR (%)**	5.93 ± 1.06	4.64 ± 0.35	5.81 ± 1.67	4.27 ± 0.32
**P-LCC (cells/L)**	2.24E+10 ± 2.93E+09	1.78E+10 ± 1.99E+09	1.44E+10 ± 2.65E+09*	1.43E+10 ± 2.21E+09*

Comprehensive panel of blood parameters measured in the REF, GLI, Clc 1:380, and Clc 1:75 groups of DQ8-D^{d}-villin-IL-15tg mice, following completion of ED2. The data are displayed and statistically analysed according to the methods outlined in Section 4.20. Statistical significance: * vs. REF; $^{\#}$ vs. GLI ($p \leq 0.05$). Abbreviations: WBC, white blood cells; LYM, lymphocytes; MID, mid-size population; GRAN, granulocytes; RBC, red blood cells; Hb, haemoglobin; Hct, haematocrit; MCV, mean corpuscular volume; MCH, mean corpuscular haemoglobin; MCHC, mean corpuscular haemoglobin concentration; RDW, red cell distribution width; PLQ, platelets; MPV, mean platelet volume; PDW, platelet volume distribution width; PCT, plateletecrit; P-LCR, platelet larger cell ratio, and P-LCC, procalcitonin.

Appendix Table S4 — Substrate gliadin content and enzyme digestion ratios.

Substrate	mg GLI / g of S[d]	mg gluten / g of S[d]				
PWG-gliadin	765	-				
gliadin (Sigma)	864	-				
Flour (Farina de Girona)	50	100				
2018 Global 18% Protein Rodent Diet; Teklad[a]	2.5	5				
***In vitro* experiments**	**Ratio (E[c]:S[d]) (w/w)**	**Ratio (E[c]:GLI) (w/w)**	**mg E[c] / g of GLI**	**S[d] (mg)**	**GLI (mg)**	**E[c] dose (mg)**
PWG-gliadin, figure 2a	1:320	1:305	3.268	0.16	0.1224	0.0004
gliadin (Sigma), figure 2a	1:375	1:350	2.9	0.16	0.1382	0.0004
Flour (Farina de Girona), figure 2b	1:10000, 1:5000, 1:1000	1:5000, 1:2500, 1:500	2, 4, 10	10	0.5	0.001, 0.002, 0.005
Gliadin, figure 2f	1:375	1:320	3.1	0.75	0.64	0.002
Flour, figure 2f	1:5000	1:250	4	10	0.5	0.001, 0.002, 0.005
Complex meal, Suppl. fig. 1C	1:5000	1:250	4	10	0.25	0.001
**Gastro-Intestinal Simulator**						
Flour (Farina de Girona), figure 2J	1:5000, 1:1000	1:250, 1:50	4	8000	400	1.6, 8
***In vivo* experiments [a]**						
PWG-gliadin for ED1 mice	1:400	1:350	2.75	5.2	4	0.11
α-gliadin for ED2 mice	1:500, 1:100	1:380, 1:75	2.5, 13.5	23	20	0.005, 0.27

[a] Values of gliadin calculated from the commercial brand, Teklad (45-55g of gluten/ Kg). [b] *In vivo* experiments gliadin ratio calculations are coming only from gliadin supplementation and not from the normal diet. [c] abbreviation of.enzyme [d] abbreviation substrate
