# Supplementary figures and images for: Targeted enzymatic therapy for coeliac disease
Source: EMBO Mol Med. 2026 May 14;18(6):2236–71. doi: 10.1038/s44321-026-00430-8 (PMC13269764; doi:10.1038/s44321-026-00430-8)

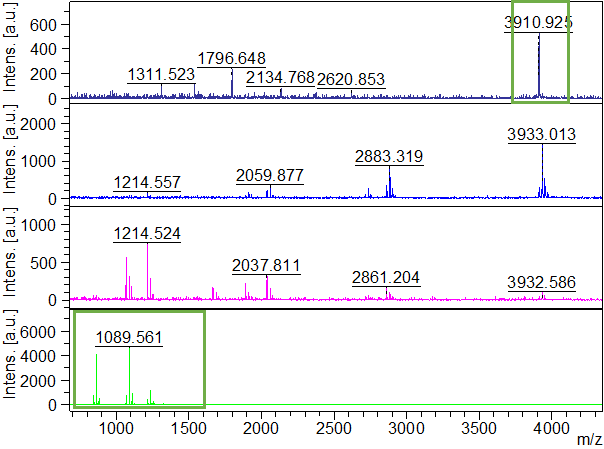

Supplement: Supplementary file 3 — Source data Fig. 1 [file 44321_2026_430_MOESM3_ESM.zip › Figure 1/1o/Graph.tif]

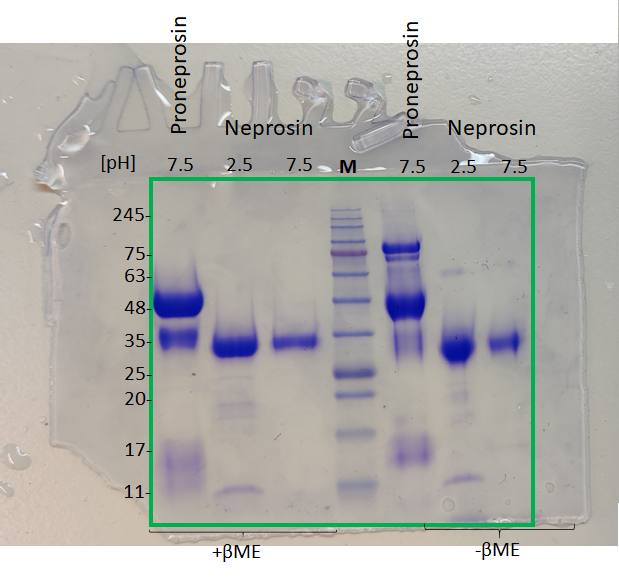

Supplement: Supplementary file 3 — Source data Fig. 1 [file 44321_2026_430_MOESM3_ESM.zip › Figure 1/1a/1a.tif]

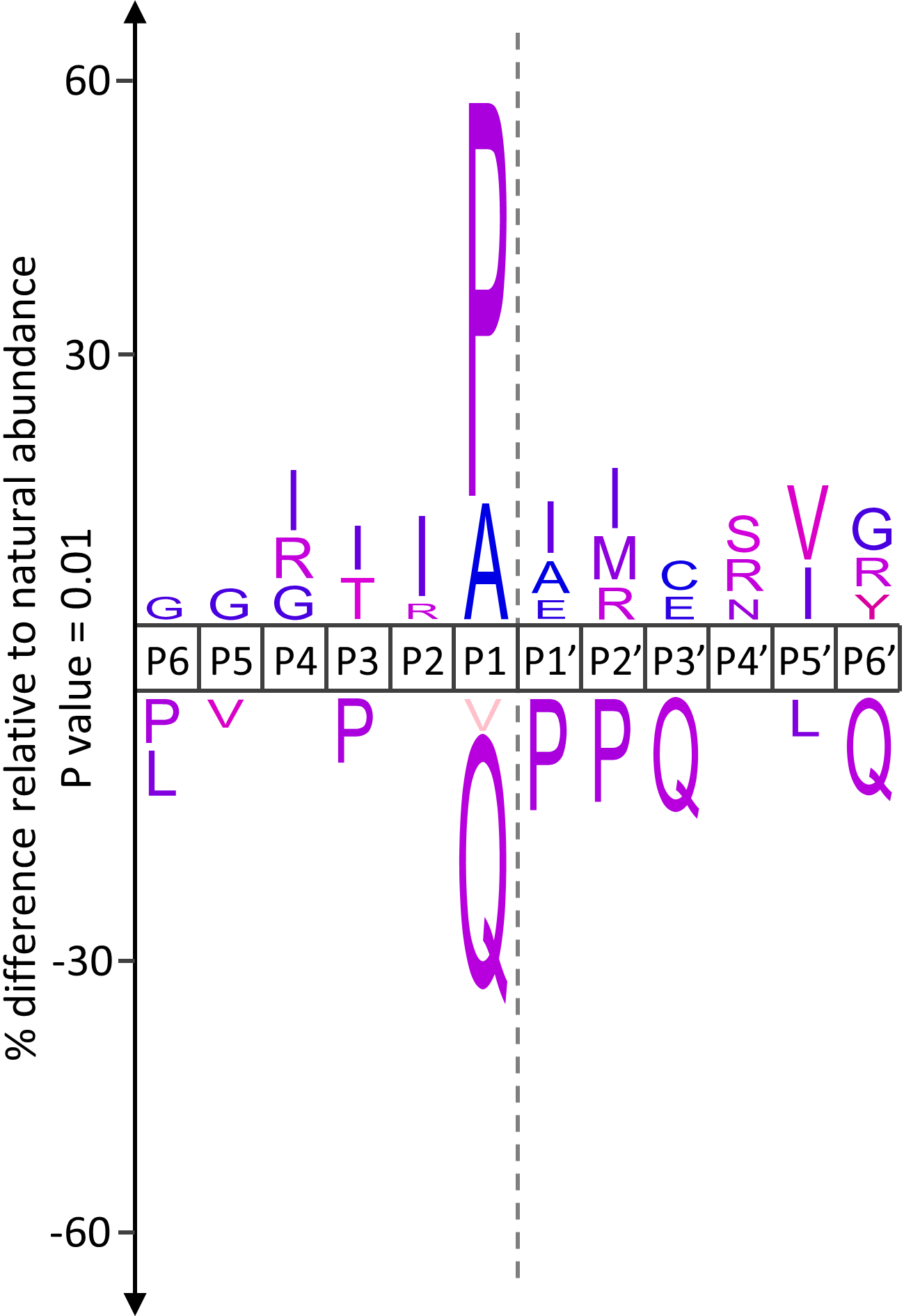

Supplement: Supplementary file 3 — Source data Fig. 1 [file 44321_2026_430_MOESM3_ESM.zip › Figure 1/1n/1n.PNG]

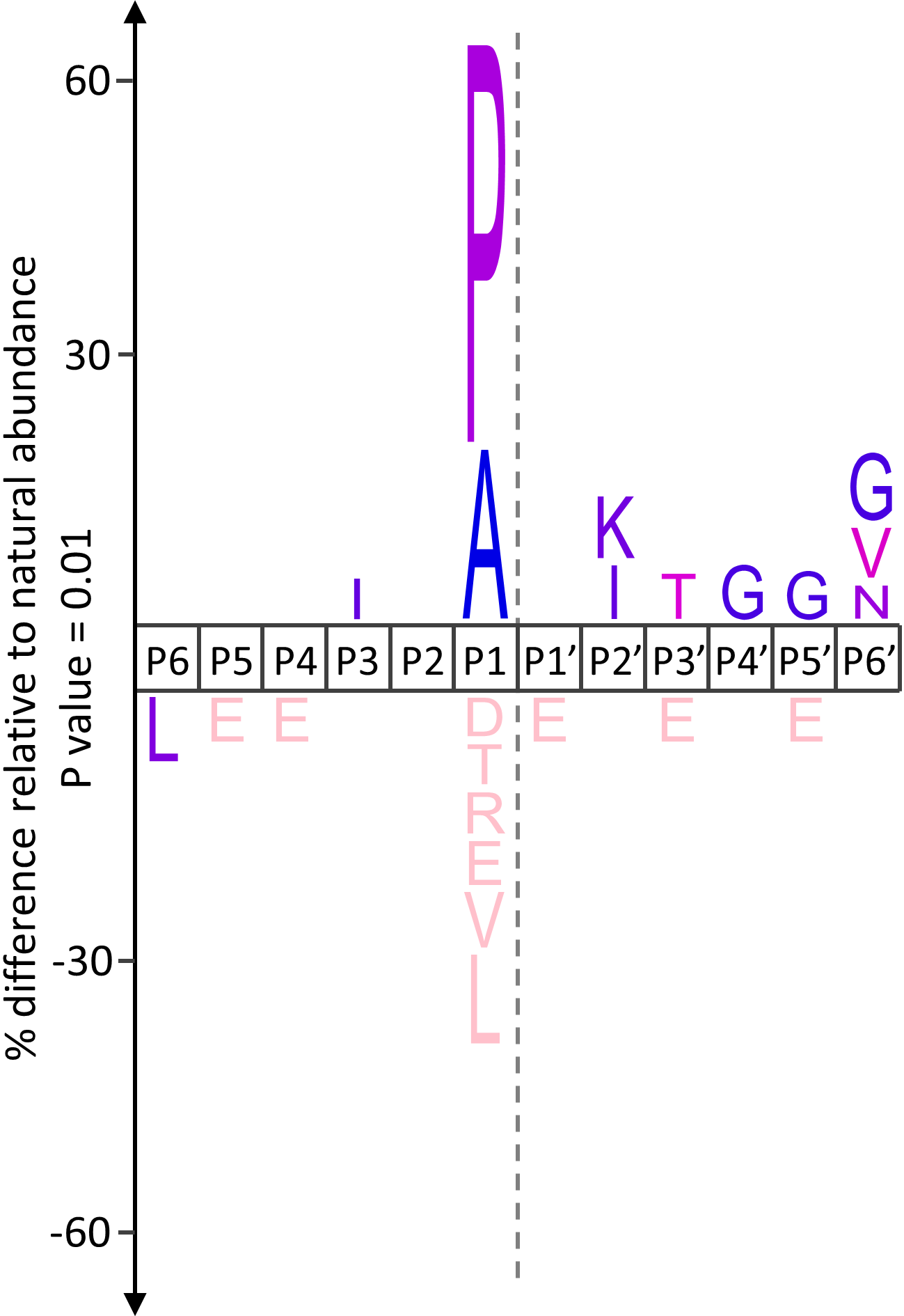

Supplement: Supplementary file 3 — Source data Fig. 1 [file 44321_2026_430_MOESM3_ESM.zip › Figure 1/1l/S1b.PNG]

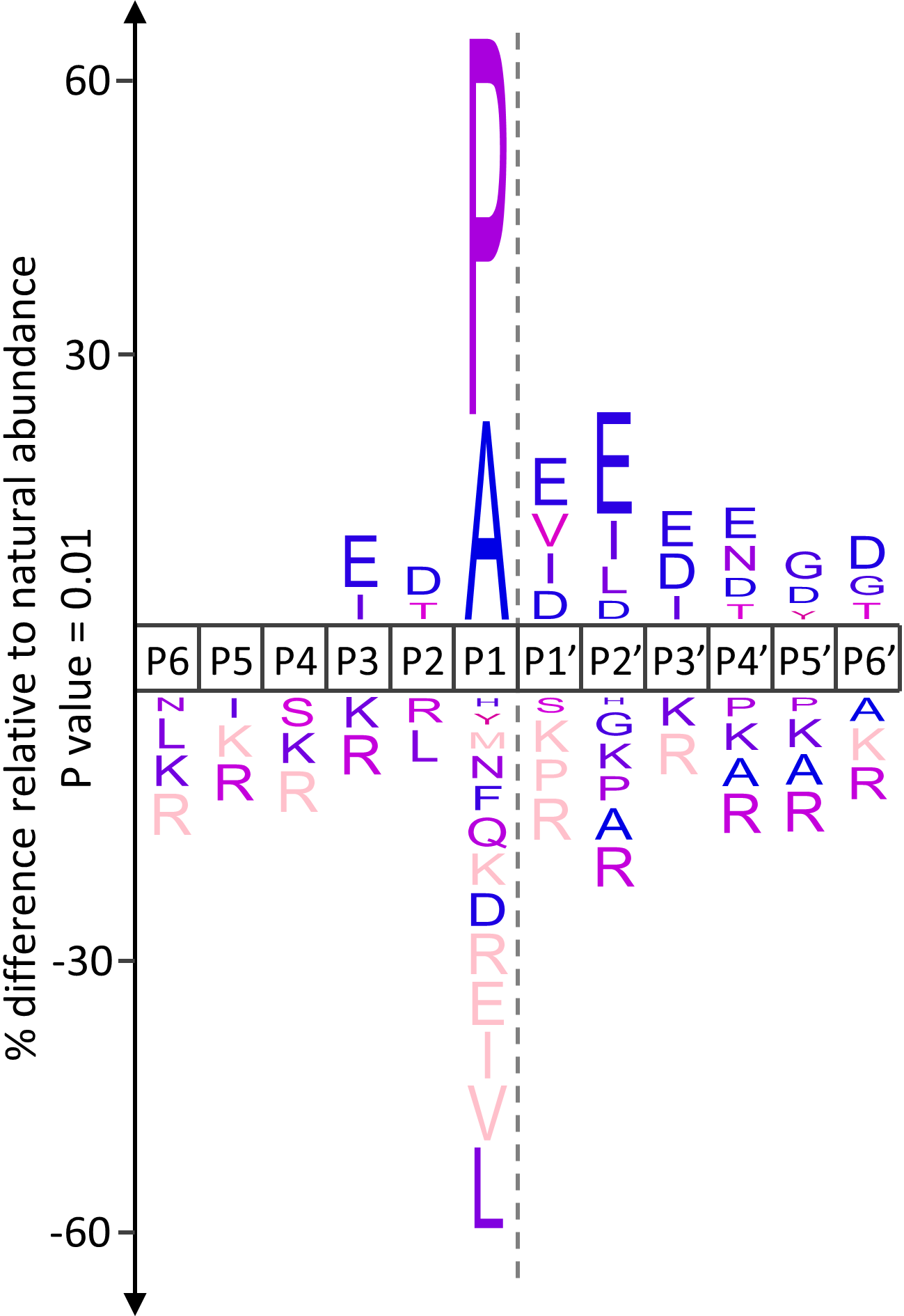

Supplement: Supplementary file 3 — Source data Fig. 1 [file 44321_2026_430_MOESM3_ESM.zip › Figure 1/1l/1l.PNG]

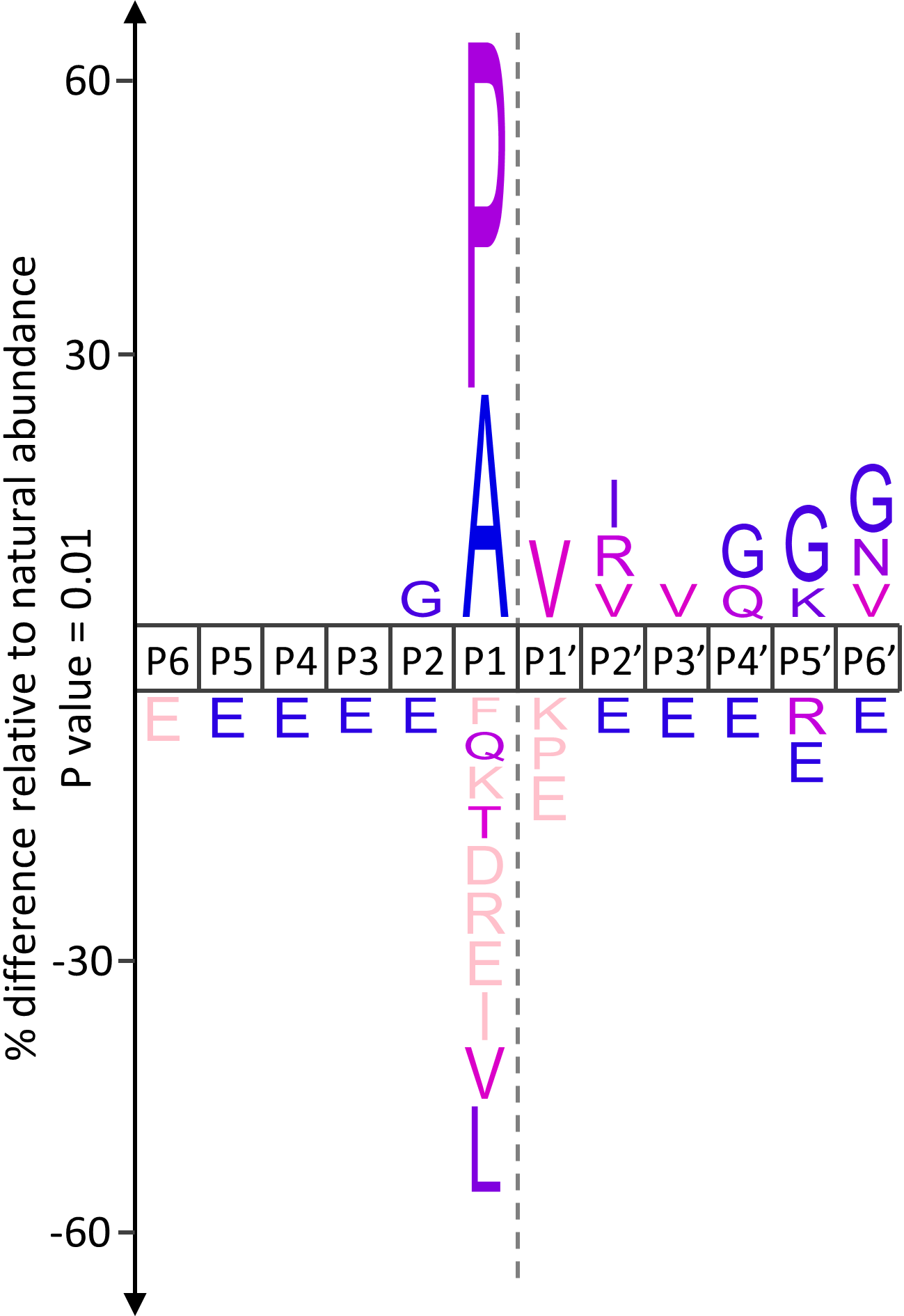

Supplement: Supplementary file 3 — Source data Fig. 1 [file 44321_2026_430_MOESM3_ESM.zip › Figure 1/1k/S1a.PNG]

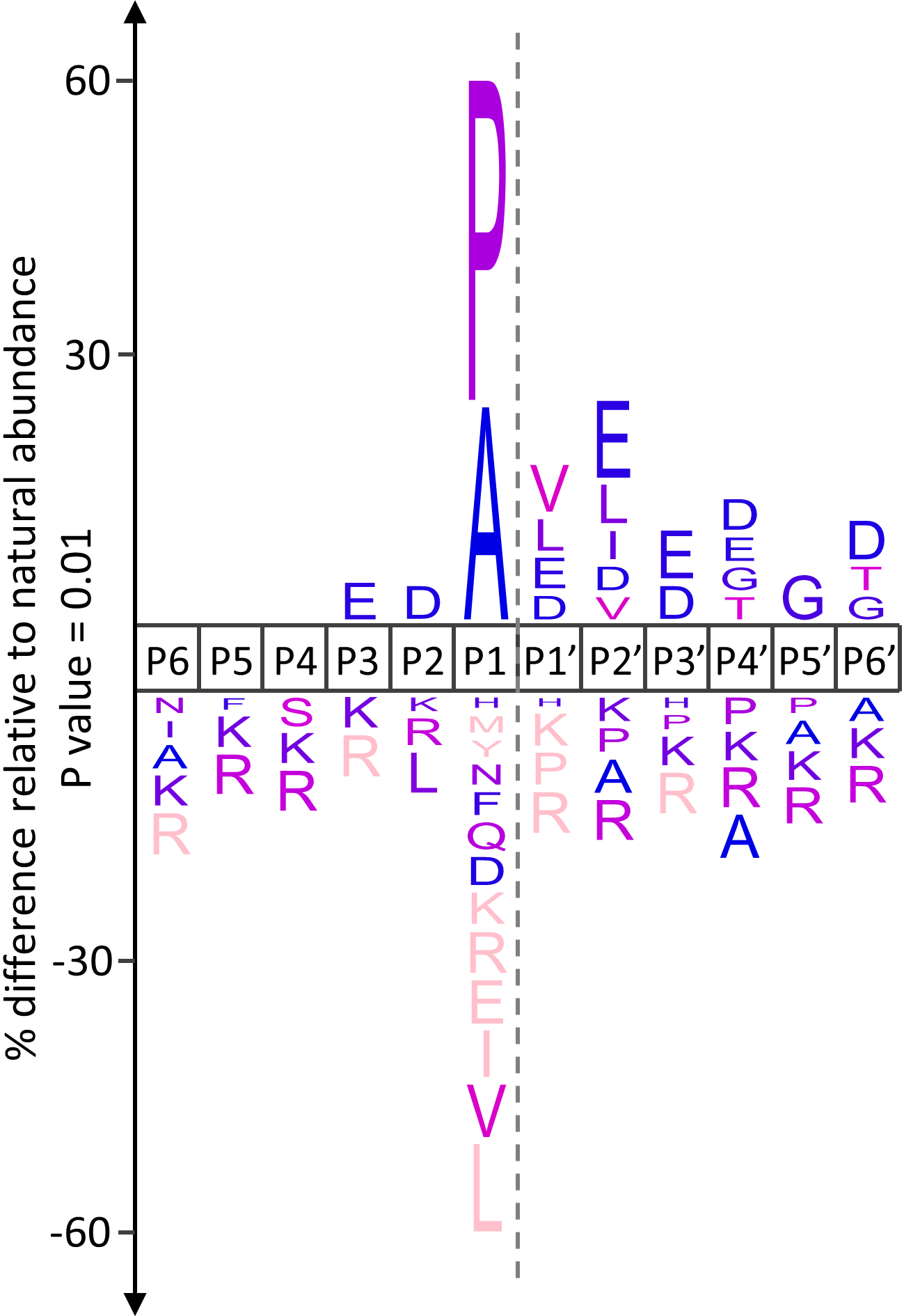

Supplement: Supplementary file 3 — Source data Fig. 1 [file 44321_2026_430_MOESM3_ESM.zip › Figure 1/1k/1k.PNG]

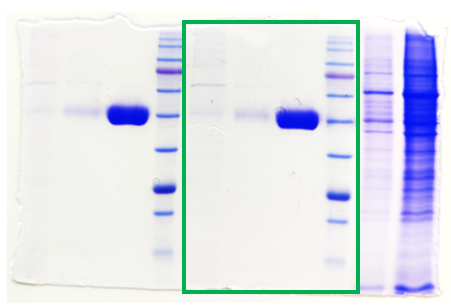

Supplement: Supplementary file 3 — Source data Fig. 1 [file 44321_2026_430_MOESM3_ESM.zip › Figure 1/1b/1b.tif]

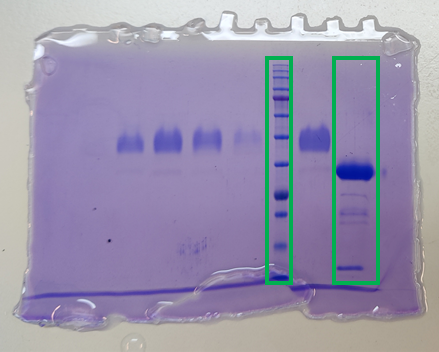

Supplement: Supplementary file 3 — Source data Fig. 1 [file 44321_2026_430_MOESM3_ESM.zip › Figure 1/1c/1c.tif]

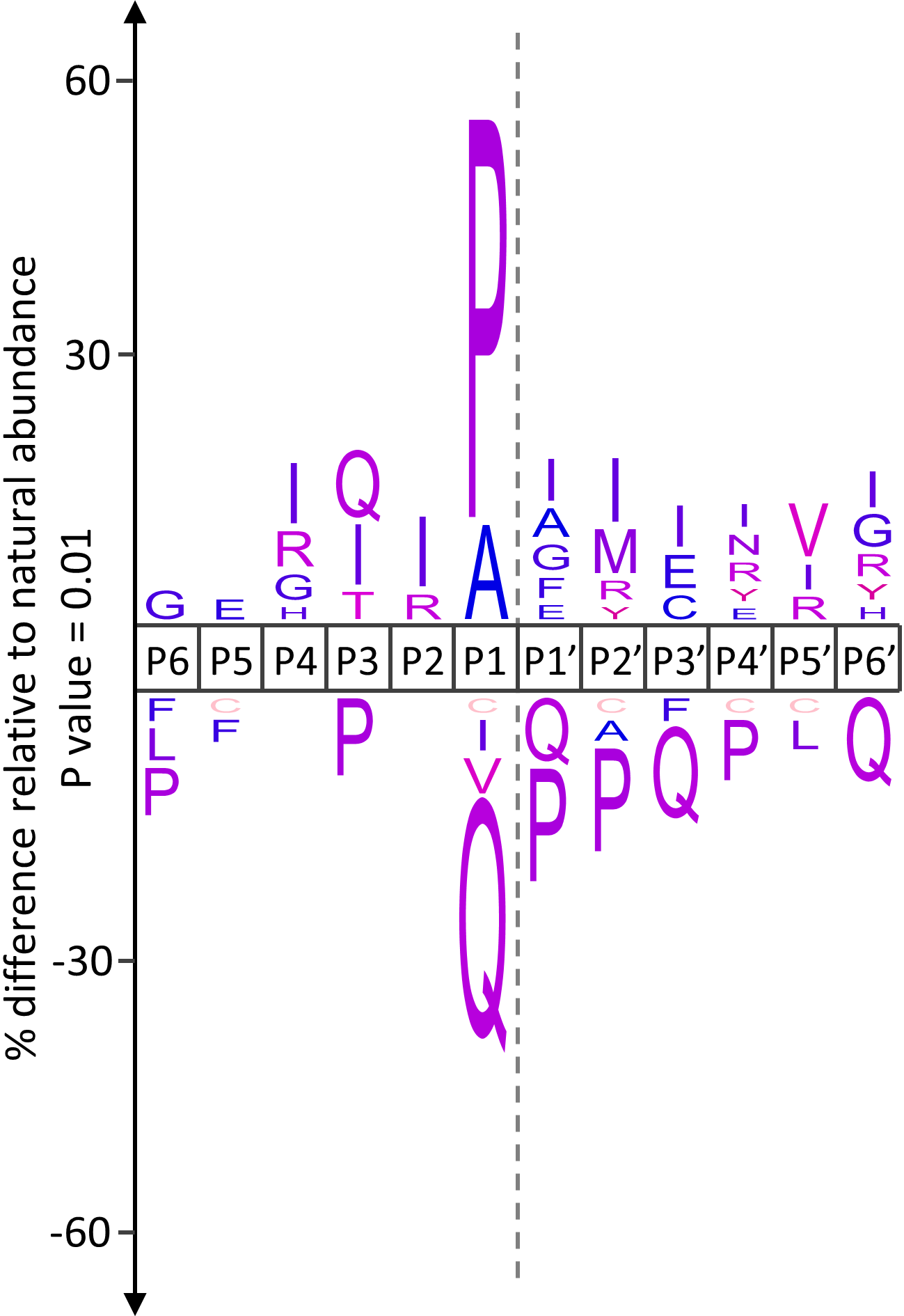

Supplement: Supplementary file 3 — Source data Fig. 1 [file 44321_2026_430_MOESM3_ESM.zip › Figure 1/1m/1m.PNG]

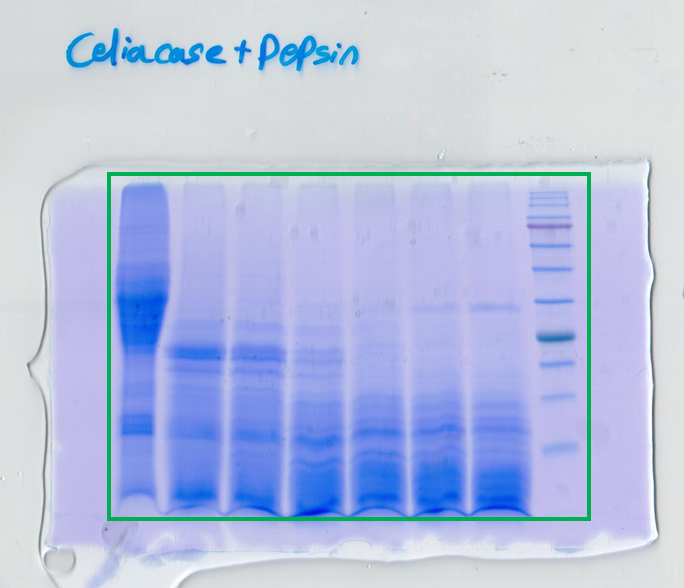

Supplement: Supplementary file 3 — Source data Fig. 1 [file 44321_2026_430_MOESM3_ESM.zip › Figure 1/1q/Celiacase & pepsin.tif]

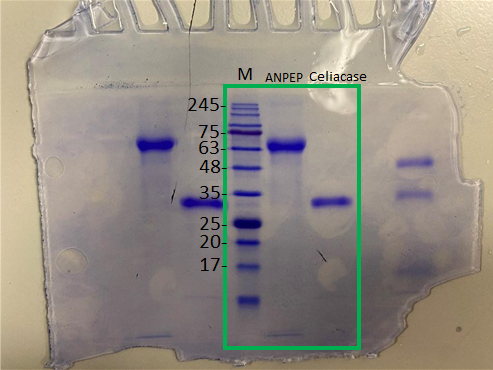

Supplement: Supplementary file 4 — Source data Fig. 2 [file 44321_2026_430_MOESM4_ESM.zip › Figure 2/2c/ANPEP & Celiacase.png]

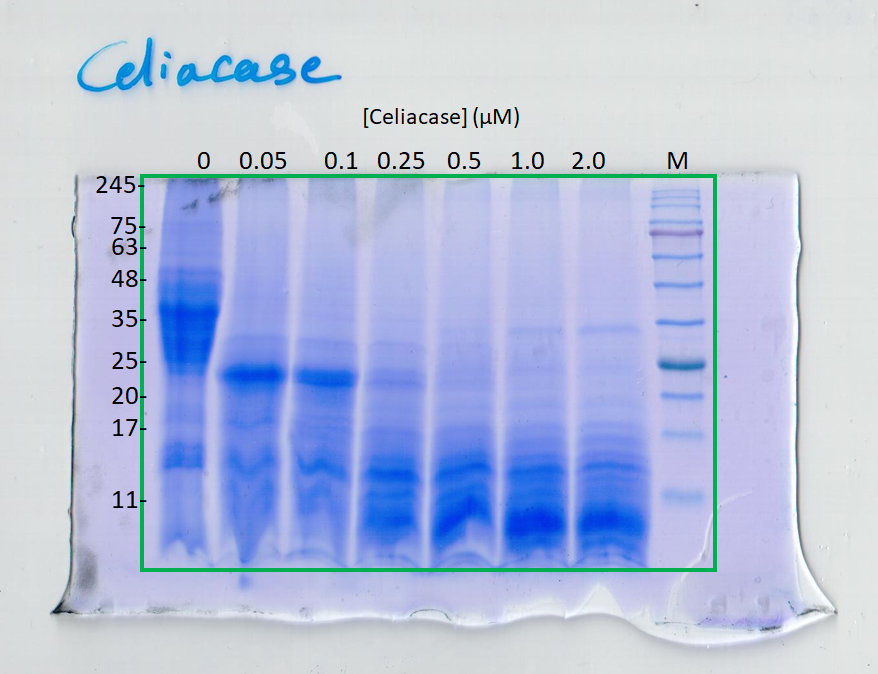

Supplement: Supplementary file 4 — Source data Fig. 2 [file 44321_2026_430_MOESM4_ESM.zip › Figure 2/2d/Celiacase.tif]

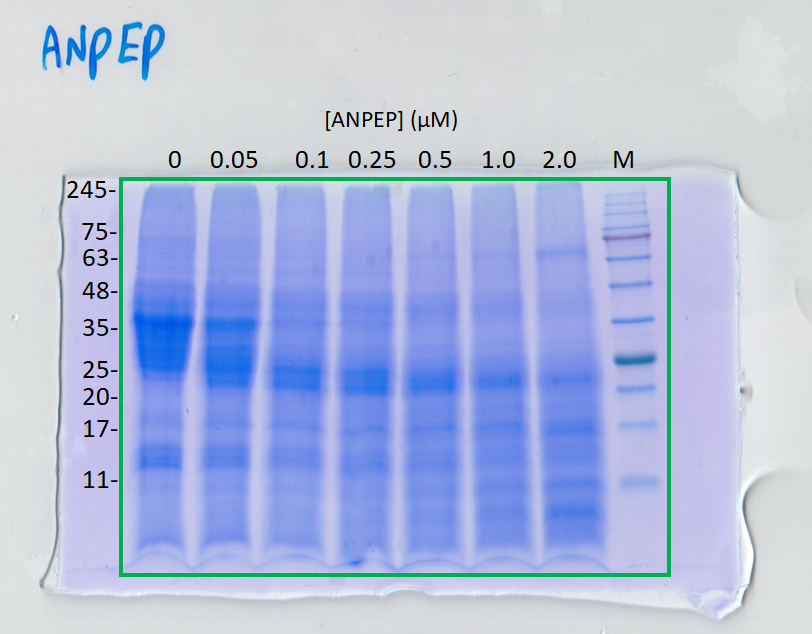

Supplement: Supplementary file 4 — Source data Fig. 2 [file 44321_2026_430_MOESM4_ESM.zip › Figure 2/2d/ANPEP.tif]

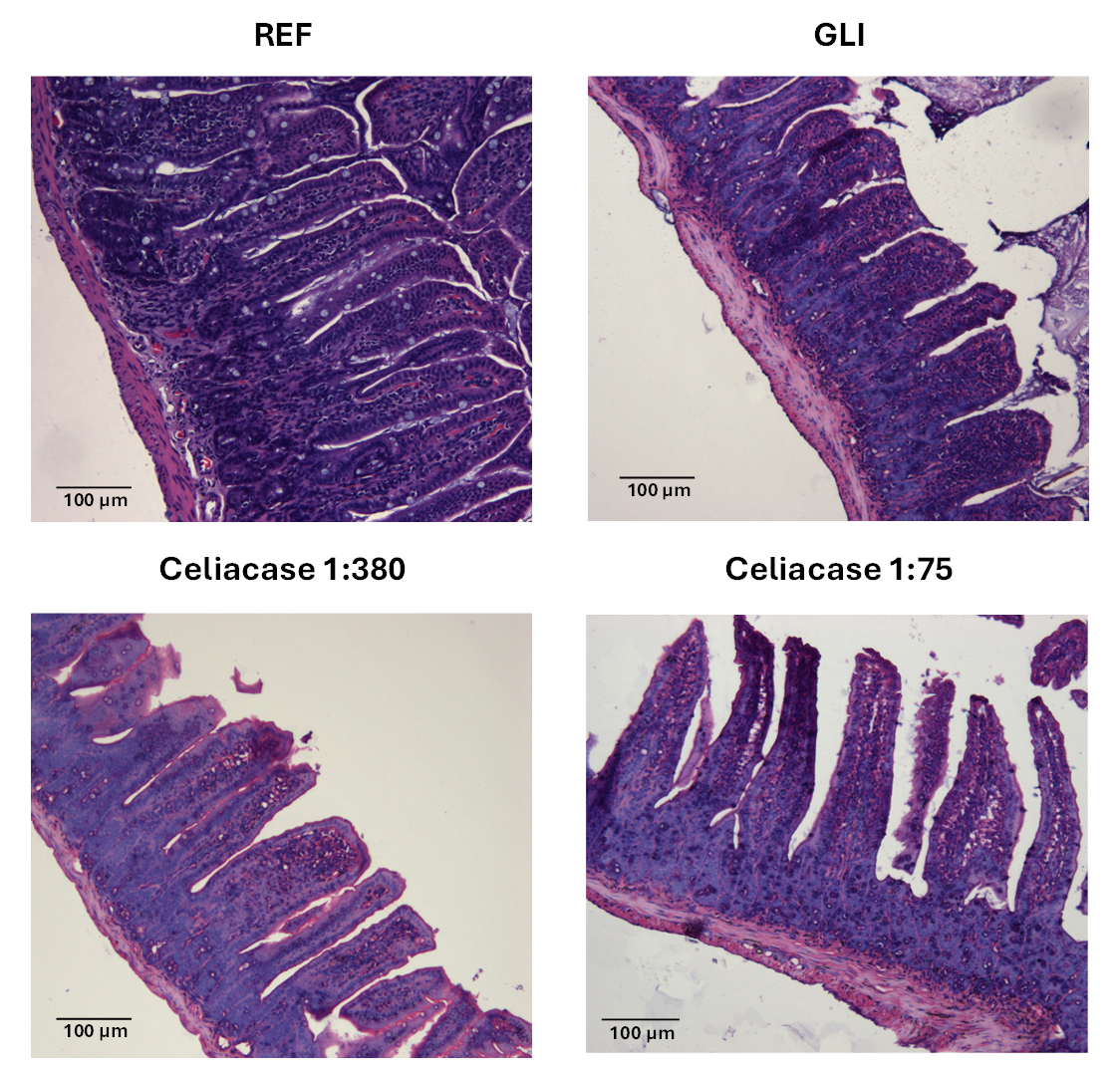

Supplement: Supplementary file 6 — Source data Fig. 4 [file 44321_2026_430_MOESM6_ESM.zip › Figure 4/4m/Fig. 4m.png]

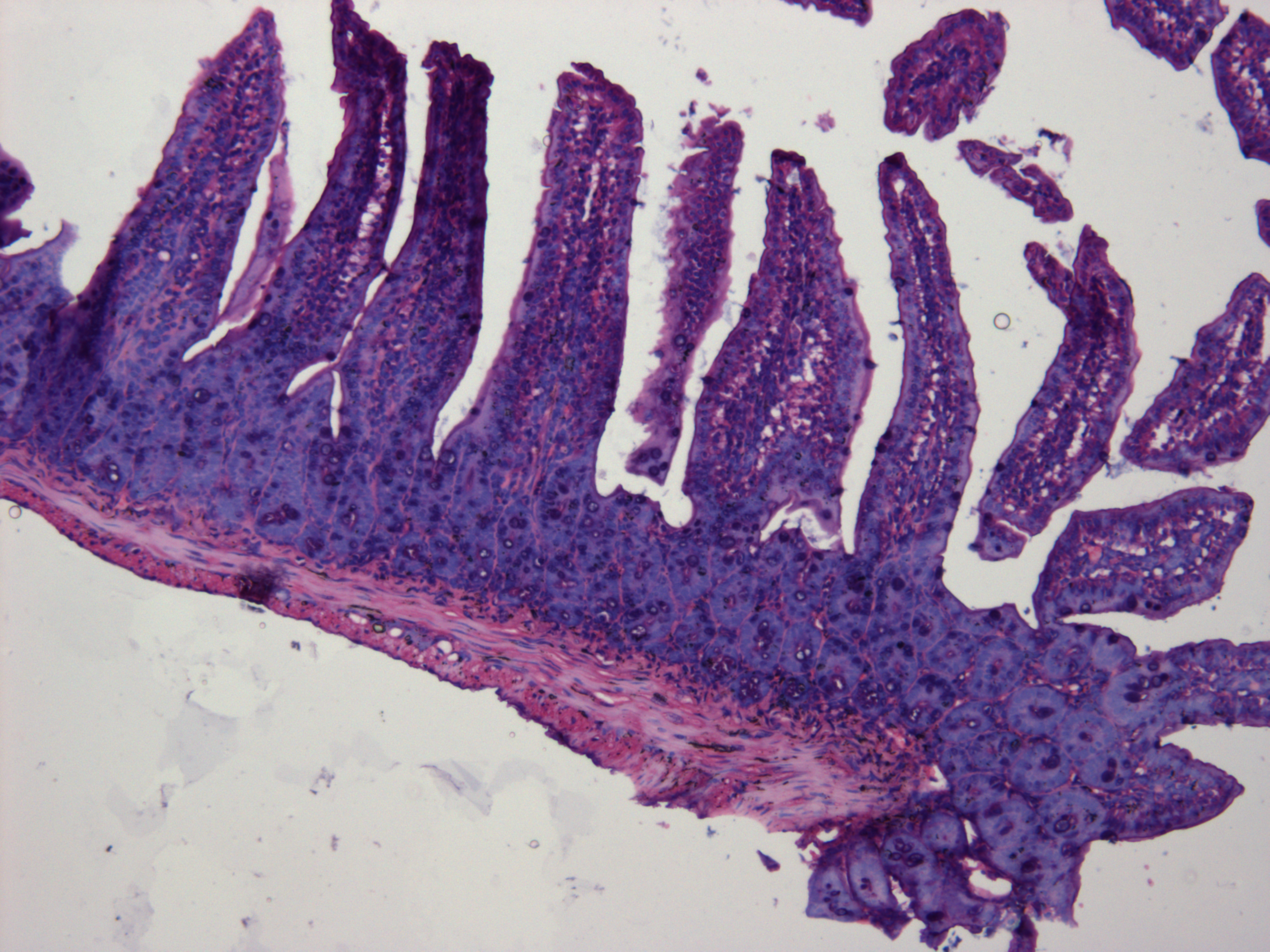

Supplement: Supplementary file 6 — Source data Fig. 4 [file 44321_2026_430_MOESM6_ESM.zip › Figure 4/4m/4m_Celiacase 75_42.1.1 10x.tif]
