# Supplementary material for: Targeted enzymatic therapy for coeliac disease
Source: EMBO Mol Med. 2026 May 14;18(6):2236–71. doi: 10.1038/s44321-026-00430-8 (PMC13269764; doi:10.1038/s44321-026-00430-8)
Supplement: Supplementary file 12 — Expanded View Figures [file 44321_2026_430_MOESM12_ESM.pdf]

Expanded View Figures

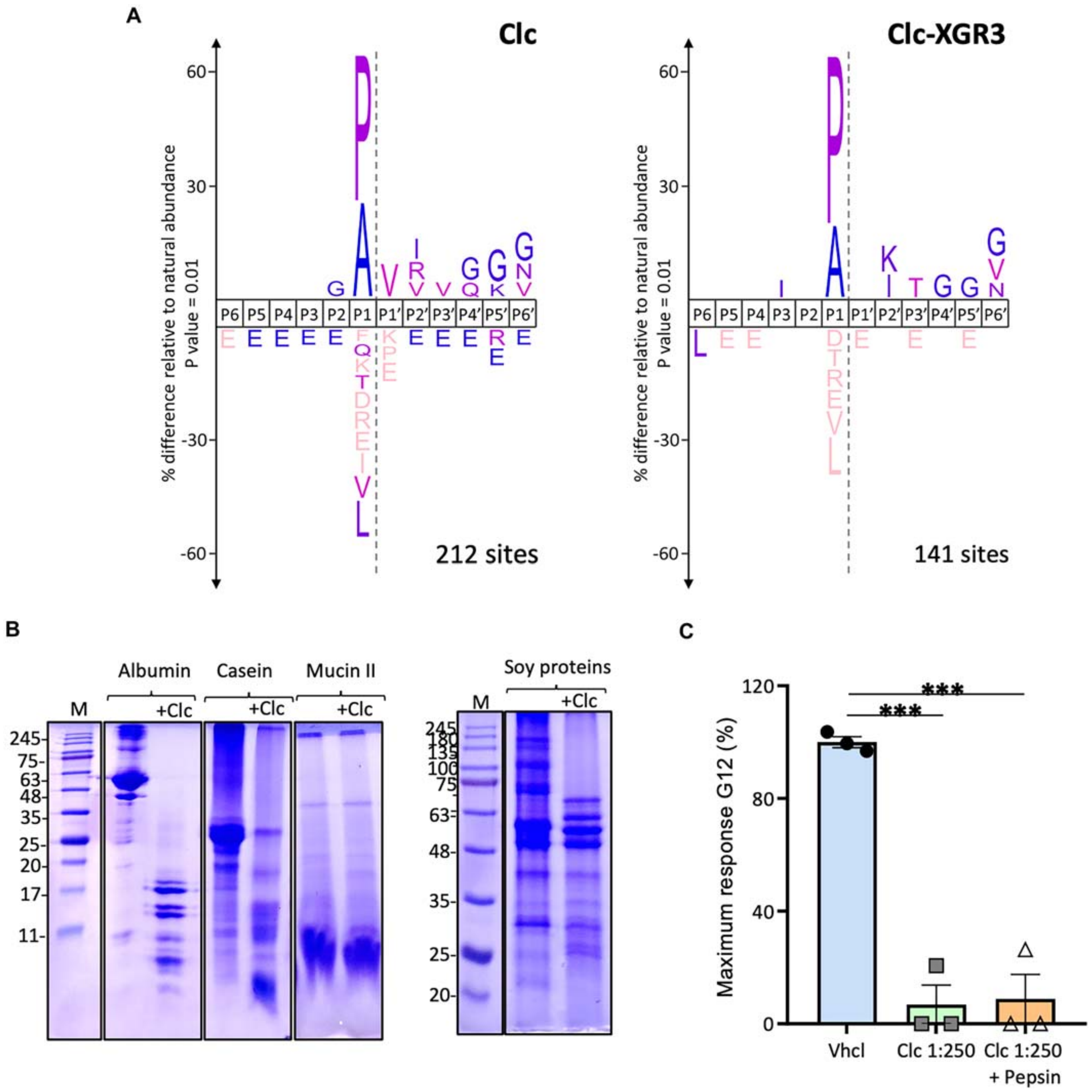

Figure EV1. Specificity profiling of celiacase and mutant Clc-XGR3 using PICS proteomics and activity against other proteins.

(**A**) The substrate specificities of celiacase (Clc) and Clc multimutant Clc-XGR3 were characterised using a GluC-derived peptide library from *Escherichia coli*. *IceLogos* visualise statistically significant ($p < 0.01$) amino-acid enrichment (above the x-axis) and depletion (below the x-axis) at non-prime (**P_6–P_1**, left) and prime (**P_1'–P_6'**, right) positions relative to the cleavage site, based on amino-acid frequencies in the *E. coli* K12 proteome. The data are derived from semi-specific cleavage peptides identified with at least two peptide-spectrum matches, and pink letters indicate amino acids not detected at a given position. (**B**) SDS–PAGE of albumin, casein, mucin II, and soy proteins after degradation by Clc at a 1:420 enzyme:substrate ratio. (**C**), ELISA ($n = 3$) of complex-meal digestion products using the G12 mAb pair, comparing Clc (1:250) and Clc (1:250) + Pepsin (0.5 µM) with control (Vhcl). Data were presented as mean ± SEM. Statistical analysis via one- or two-way ANOVA: *$p \leq 0.05$, **$p \leq 0.01$, ***$p \leq 0.001$.

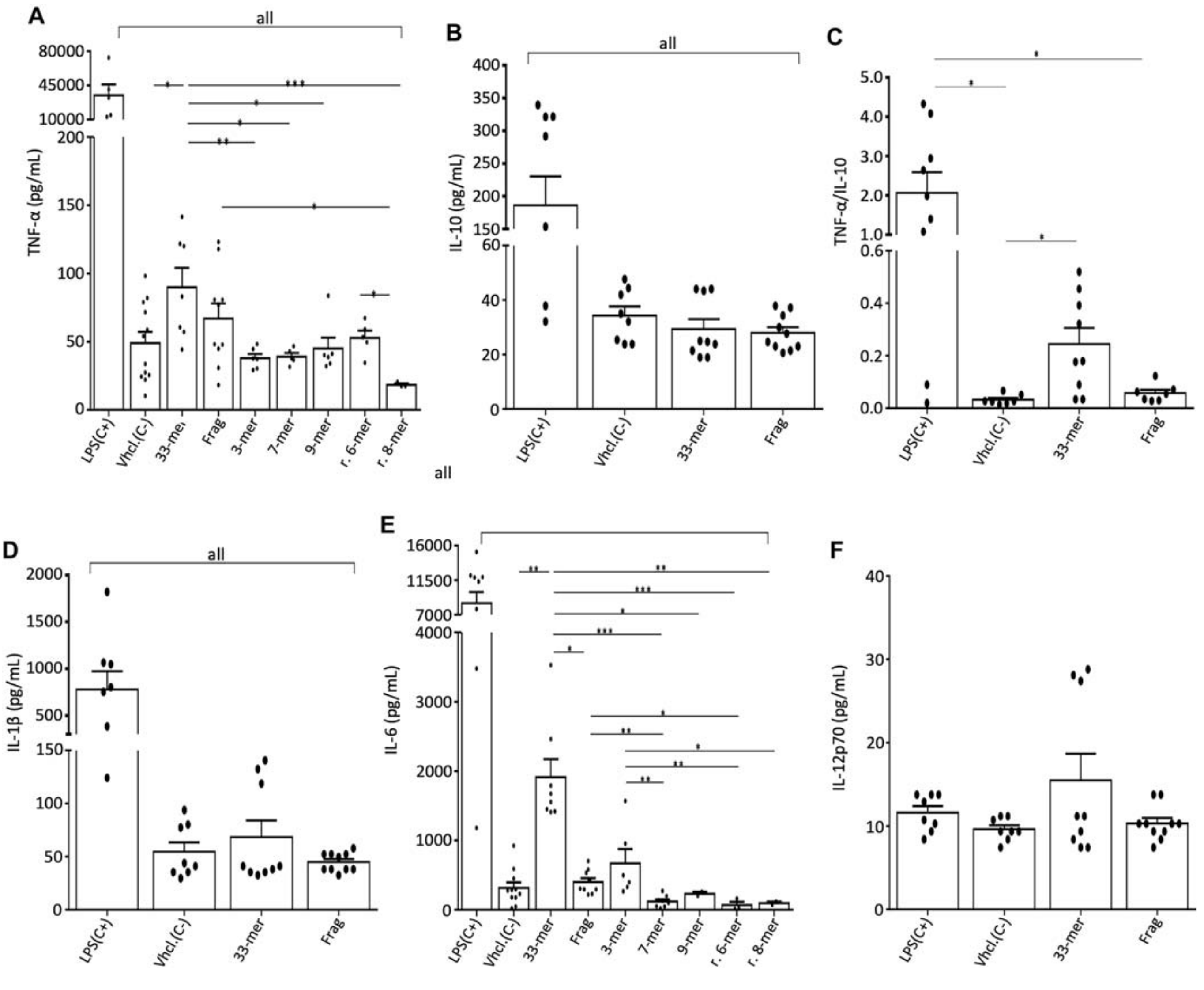

Figure EV2. Immune response of rat macrophages upon stimulation with the 33-mer and its fragments.

(**A–F**) Assessment of inflammatory cytokine secretion by peritoneal rat macrophages ($n = 24$) following stimulation with the 33-mer, its Clc cleavage fragments (3-mer, 7-mer, 9-mer and combined), or random (r) 6-mer (T A T R G G) and 8-mer (A T L A K V S H) peptides. Only the most significant cytokines are reported. Cytokine levels of TNF-α (**A**), IL-10 (**B**), TNF-α/IL-10 ratio (**C**), IL-1β (**D**), IL-6 (**E**), IL-12p70 (**F**); all stimuli at 4.25 mM, except (**A**) at 0.25 mM.

© The Author(s)

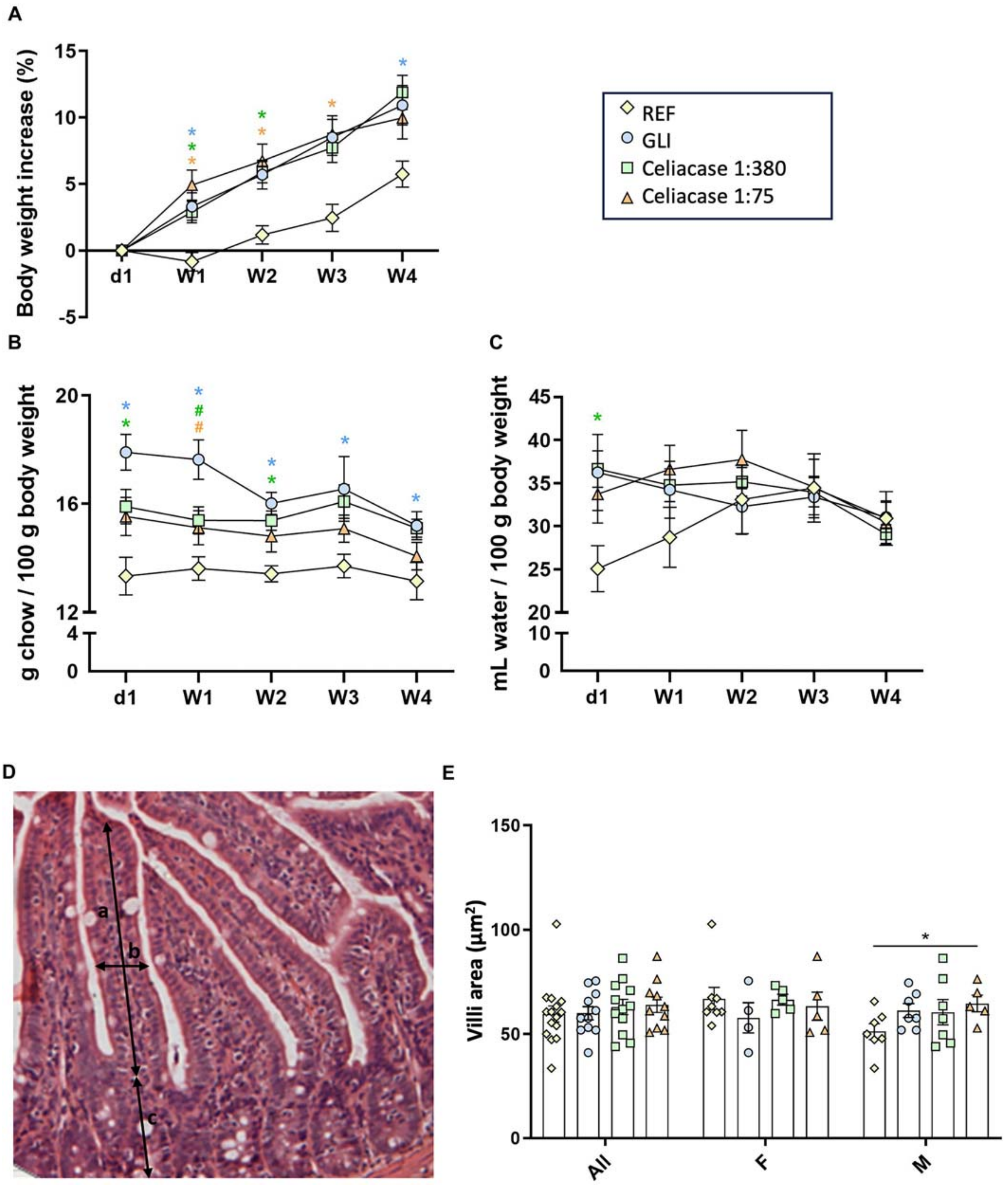
A
Body weight increase (%)
15
10
5
0
-5
d1 W1 W2 W3 W4
REF
GLI
Celiacase 1:380
Celiacase 1:75
B
g chow / 100 g body weight
20
16
12
4
0
d1 W1 W2 W3 W4
C
mL water / 100 g body weight
45
40
35
30
25
20
10
0
d1 W1 W2 W3 W4
D
a
b
c
E
Villi area (μm2)
150
100
50
0
All F M

Figure EV3. Body weight, food and water intake of DQ8-D^d-villin-IL-15tg mice during the ED2 intervention, and post-intervention histological analysis.

(**A–C**) Percentage increase in body weight, chow consumption, and water intake at day 1 (d1) and weeks 1–4 (W1–W4) within the 25-day experiment for the REF, GLI; Clc 1:380, and Clc 1:75 groups. Symbol colours indicate significant differences: $^*p \leq 0.05$ vs. REF; $^\#p \leq 0.05$ vs. GLI. (**D**) Schematic representation of histological measurements: a = villi length, b = villi width, c = crypt depth, a × b = villi area, b/c = villi length-to-crypt depth ratio. (**E**) Villi area of the distal small intestine split into the REF, GLI, Clc 1:380 and Clc 1:75 groups. In all cases, $n = 7$ per group and sex, results are expressed as mean ± SEM for females (F), males (M), and combined (all), * stands for $p \leq 0.05$ vs. REF. Statistical analyses are as described in section "Statistical and data analysis".

 © The Author(s)
