# Supplementary material for: Targeted enzymatic therapy for coeliac disease
Source: EMBO Mol Med. 2026 May 14;18(6):2236–71. doi: 10.1038/s44321-026-00430-8 (PMC13269764; doi:10.1038/s44321-026-00430-8)
Supplement: Supplementary file 2 — Peer Review File [file 44321_2026_430_MOESM2_ESM.pdf]

EMBO Molecular Medicine

Targeted enzymatic therapy for coeliac disease

Marina Girbal-Gonzalez, Arturo Rodriguez-Banqueri, Hadeel Swaid, Soraia Mendes, Laura Garzon-Flores, Juan Sebastian Ramirez-Larrota, Carolina Cueva, M. Victoria Moreno-Arribas, Christof Regl, Christian Huber, Katharina Scherf, Maria Jose Rodriguez-Lagunas, Angels Franch-Masferrer, Ulrich Eckhard, Francisco Jose Perez-Cano, and F. Xavier Gomis-Rüth

Corresponding authors: F. Xavier Gomis-Rüth (xgrcri@ibmb.csic.es) , Francisco Jose Perez-Cano (franciscoperez@ub.edu)

Review Timeline:

Submission Date:	11th Nov 25
Editorial Decision:	9th Jan 26
Revision Received:	9th Mar 26
Editorial Decision:	23rd Mar 26
Revision Received:	8th Apr 26
Accepted:	8th Apr 26

Editor: Lise Roth

Transaction Report:

(Note: With the exception of the correction of typographical or spelling errors that could be a source of ambiguity, letters and reports are not edited. Depending on transfer agreements, referee reports obtained elsewhere may or may not be included in this compilation. Referee reports are anonymous unless the Referee chooses to sign their reports.)

9th Jan 2026

Dear Prof. Gomis-Rüth,

Thank you for the submission of your manuscript to our editorial offices, and please accept my apologies for the delay in getting back to you. As previously explained, one referee became unresponsive and had to be withdrawn from the process, and we subsequently had difficulties securing referees with the right expertise at this time of the year. We have now received feedback from the three reviewers who agreed to evaluate your study. As you will see from the reports below, the reviewers acknowledge the novelty, quality and potential translational interest of the findings, but nevertheless raise a few concerns on your study. If you feel you can address the reviewers' comments satisfactorily, you may wish to submit a revised version of your manuscript.

Addressing the reviewers' concerns in full will be necessary for further considering the manuscript in our journal, and acceptance of the manuscript will entail a second round of review. EMBO Molecular Medicine encourages a single round of revision only and therefore, acceptance or rejection of the manuscript will depend on the completeness of your responses included in the next, final version of the manuscript. For this reason, and to save you from any frustrations in the end, I would strongly advise against returning an incomplete revision.

If you would like to discuss further the points raised by the referees, I am available to do so via email or video. Let me know if you are interested in this option.

We are expecting your revised manuscript within three months, if you anticipate any delay, please contact us.

When preparing your revised manuscript, please refer to our guidelines: https://link.springer.com/journal/44321/submission-guidelines#cms-Revised-submissions. We perform an initial quality control of all revised manuscripts before re-review; failure to include requested items will delay the evaluation of your revision.

We require:

1) A .docx formatted version of the manuscript text (including legends for main figures, EV figures and tables). Please make sure that the changes are highlighted to be clearly visible.

2) Individual production quality figure files as .eps, .tif, .jpg (one file per figure). For guidance, download the 'Figure Guide PDF': https://media.springernature.com/original/springer-cms/rest/v1/content/27825798/data/v1.

3) A .docx formatted letter INCLUDING the reviewers' reports and your detailed point-by-point responses to their comments. As part of the EMBO Press transparent editorial process, the point-by-point response is part of the Review Process File (RPF), which will be published alongside your paper.

4) A complete author checklist, which you can download from our author guidelines. Please insert information in the checklist that is also reflected in the manuscript. The completed author checklist will also be part of the RPF.

5) Please note that all corresponding authors are required to supply an ORCID ID for their name upon submission of a revised manuscript.

6) It is mandatory to include a 'Data Availability' section after the Materials and Methods. Before submitting your revision, primary datasets produced in this study need to be deposited in an appropriate public database, and the accession numbers and database listed under 'Data Availability'. Please remember to provide a reviewer password if the datasets are not yet public.

In case you have no data that requires deposition in a public database, please state so in this section. Note that the Data Availability Section is restricted to new primary data that are part of this study.

7) For data quantification: please specify the name of the statistical test used to generate error bars and P values, the number (n) of independent experiments (specify technical or biological replicates) underlying each data point and the test used to calculate p-values in each figure legend. The figure legends should contain a basic description of n, P and the test applied. Graphs must include a description of the bars and the error bars (s.d., s.e.m.).

8) At EMBO Press we ask authors to provide source data for the main manuscript figures. You will receive a separate email with instructions for providing source data with your revised manuscript, including how to upload and organize the files.

9) Our journal encourages inclusion of *data citations in the reference list* to directly cite datasets that were re-used and obtained from public databases. Data citations in the article text are distinct from normal bibliographical citations and should directly link to the database records from which the data can be accessed. In the main text, data citations are formatted as

follows: "Data ref: Smith et al, 2001" or "Data ref: NCBI Sequence Read Archive PRJNA342805, 2017". In the Reference list, data citations must be labeled with "[DATASET]". A data reference must provide the database name, accession number/identifiers and a resolvable link to the landing page from which the data can be accessed at the end of the reference.

10) We replaced Supplementary Information with Expanded View (EV) Figures and Tables that are collapsible/expandable online. A maximum of 5 EV Figures can be typeset. EV Figures should be cited as 'Figure EV1, Figure EV2" etc... in the text and their respective legends should be included in the main text after the legends of regular figures.

- For the figures that you do NOT wish to display as Expanded View figures, they should be bundled together with their legends in a single PDF file called *Appendix*, which should start with a short Table of Content. Appendix figures should be referred to in the main text as: "Appendix Figure S1, Appendix Figure S2" etc.

- Additional Tables/Datasets should be labeled and referred to as Table EV1, Dataset EV1, etc. Legends have to be provided in a separate tab in case of .xls files. Alternatively, the legend can be supplied as a separate text file (README) and zipped together with the Table/Dataset file.

11) The paper explained: EMBO Molecular Medicine articles are accompanied by a summary of the articles to emphasize the major findings in the paper and their medical implications for the non-specialist reader. Please provide a draft summary of your article highlighting
- the medical issue you are addressing,
- the results obtained and
- their clinical impact.

This may be edited to ensure that readers understand the significance and context of the research. Please refer to any of our published articles for an example.

12) Author contributions: You will be asked to provide CRediT (Contributor Role Taxonomy) terms in the submission system. These replace a narrative author contribution section in the manuscript.

13) A "Disclosure and Competing Interests Statement" should be provided in the main text.

14) Every published paper includes a 'Synopsis' to further enhance discoverability. Synopses are displayed on the journal webpage and are freely accessible to all readers. They include a short stand first (maximum of 300 characters, including space) as well as 2-5 one-sentences bullet points that summarizes the paper. Please write the bullet points to summarize the key NEW findings. They should be designed to be complementary to the abstract - i.e. not repeat the same text. We encourage inclusion of key acronyms and quantitative information (maximum of 30 words / bullet point). Please use the passive voice. Please attach these in a separate file or send them by email, we will incorporate them accordingly.

Please also suggest a striking image or visual abstract to illustrate your article as a PNG file 550 px wide x 300-600 px high.

15) Include a Reagents and Tools Table as part of the Methods section, which can be downloaded from our author guidelines.

EMBO Molecular Medicine has a "scooping protection" policy, whereby similar findings that are published by others during review or revision are not a criterion for rejection. Should you decide to submit a revised version, I do ask that you get in touch after three months if you have not completed it, to update us on the status.
Molecular Medicine

I look forward to receiving your revised manuscript.

Yours sincerely,

Lise Roth

Lise Roth, PhD
Senior Editor
EMBO Molecular Medicine

***** Reviewer's comments *****

Referee #2 (Remarks for Author):

The work by Girbal-González et al. presents the development of celicase, with an emphasis on the activity of this endopeptidase in both in vitro and in vivo studies. The research has been conducted very thoroughly, taking into account the multifactorial nature of coeliac disease and employing experimental models that closely reflect the physiology of the digestive tract. The introduction provides a comprehensive overview of the current state of knowledge and convincingly justifies the need for this research. The results obtained constitute a valuable contribution, indicating the potential for clinical application of celicase.
Specific comments are provided below.
1. The figures require improvement: the font size is too small and the chart labels are largely illegible.
2. All in vivo studies were performed in mice; therefore, the use of rat macrophages for testing (Fig. 3) is puzzling. Substituting mouse macrophages might facilitate the interpretation of data obtained from animal experiments.
3. Lines 427-428: the statement "By contrast, the fragments did not significantly differ from vehicle-except for IL-2, indicating a markedly reduced immunogenic profile" does not correspond to the data presented in Fig. 3i and j.
4. Line 558: the statement "Moreover, Clc 1:75 prevented the significant reduction in IgG2b observed in the GLI group relative to REF" does not correspond to the data presented in Fig. 5a.
5. Line 556: the statement "At the intestinal level, most Ig isotypes were increased in GLI compared with REF, particularly in males, an effect prevented by Clc treatment at both doses (Fig. 5f)" is not supported by the data shown in the corresponding figure. How do the authors explain the significant increase in IgG1 following celicase treatment?
6. Line 585: the statement "Intestinal concentrations of three key pro-inflammatory cytokines associated with CD-IFN-γ, IL-6, and TNF-α-were significantly elevated in GLI mice compared with REF as a result of gliadin intake (Fig. 6a-c). These increments were prevented by Clc treatment, specifically for IFN-γ and TNF-α in males and IL-6 in females" does not correspond to the presented data, as GLI appears to strongly induce these inflammatory markers only in males. How do the authors explain the striking sex-dependent differences?
7. Line 608: „CD8α$^-$CD4$^+$ double-positive T cells" should be corrected to CD8α$^+$CD4$^+$.

Referee #3 (Remarks for Author):

Reading this article was truly enjoyable for several reasons: the way it's written, the enormous scientific contribution it can make, and the large amount of data provided. I really appreciated it. I have several considerations to make that I think could increase the scientific impact of the article.
1. Across the manuscript, enzyme dosing is expressed variously as w/w ratios, mg/mL concentrations, and sometimes in mg enzyme without reference to gliadin mass. This inconsistency makes comparison across experiments difficult. The Simgi enzyme:gliadin ratios (1:250 and 1:50) are not mathematically consistent with the reported enzyme concentrations (0.16 mg/mL; 0.80 mg/mL). Please provide the full dose calculation and include a table expressing all doses as mg enzyme per g gliadin and mg per typical human meal. Also, because this data could be very useful for defining the quantities for an oral formulation.

2. There is no evaluation of off-target digestion or meal-dependent buffering. Please test Clc against representative food and host proteins (mucin, albumin, casein, soy) at gastric pH, or discuss this limitation. A minimal off-target panel is strongly recommended:
a. mucin
b. albumin
c. casein
d. soy proteins
e. representative complex meals with high fat or protein content (high buffering capacity).
I think with this information, your data would gain even more strength.

3. Figures 2i-j appear to show more data points than the stated triplicates. Please clarify n for biological and technical replicates and whether multiple time-points or sampling ports were included.

4. In vivo peptide degradation (Section 2.7) lacks fundamental dosing information: total gliadin consumed, relevance to human meal loads, and whether digestion was assessed with complex food matrices.

5. The reported female-selective histological improvement is not supported by the cited literature. Line 550 refers to an improvement in histological damage, especially in female mice. The discussion (lines 855-857) refers to possible differences between the sexes. While this is true, the cited article doesn't mention any gender differences in the degree of villous atrophy (nor do more recent articles such as doi:10.3390/nu14153192). So, how do you think this can be explained?

Referee #4 (Comments on Novelty/Model System for Author):

In the present manuscript the authors describe in vitro and in vivo characterization of engineered neprosin, that differs from the original enzyme by a Cys334Val mutation, and exhibited substantially improved properties. Both in vitro and in vivo studies were very comprehensive, including ex vivo studies with a dynamic gastrointestinal simulator, a human digestion model, and in vivo studies in a mouse CD model. Celiacase was very efficient in degrading gluten, reducing immune response and inflammation, as well as restoring microbial metabolic pathways and immune-regulatory markers. They have also demonstrated that their celiacase is more efficient in gluten degradation as Aspergillus niger prolyl endopeptidase (AN-PEP) that is routinely used as a food supplement. I found the study technically sound and very interesting. However, I am not a specialist in CD disease and can really only comment more on the protease part of the study, which has been done very well.

Referee #4 (Remarks for Author):

The idea of using prolyl endopeptidases as food additives to treat celiac disease is not novel and has already been explored in the past for over 20 years and has already been introduced in the CD treatment. This holds true also for the authors, who have described a detailed characterization and structure of neprosin, a plant prolyl endopeptidase from the pitcher plant, including its in vivo effect in mice on gliadin degradation.

In the present manuscript the authors describe in vitro and in vivo characterization of engineered neprosin, that differs from the original enzyme by a Cys334Val mutation, and exhibited substantially improved properties. Both in vitro and in vivo studies were very comprehensive, including ex vivo studies with a dynamic gastrointestinal simulator, a human digestion model, and in vivo studies in a mouse CD model. Celiacase was very efficient in degrading gluten, reducing immune response and inflammation, as well as restoring microbial metabolic pathways and immune-regulatory markers. They have also demonstrated that their celiacase is more efficient in gluten degradation as Aspergillus niger prolyl endopeptidase (AN-PEP) that is routinely used as a food supplement. I found the study technically sound and very interesting.

Anyhow, I have few comments:

Authors consistently use the term protease concentration and it seems that they are referring to protein concentration rather than to the active concentration of the protease, which may differ drastically. This is normally determined with active site titration, which was not performed here. While it seems that celiacase (Clc) has considerable activity based on the assays performed with pure enzyme, there is zero evidence of the specific activity of the Aspergillus niger prolyl endopeptidase (AN-PEP) which they used as a comparison. They have purified it from GliadinX (it is GliadinX not Giadin X - please correct in the legend) capsules as shown in Fig 2c, but no specific assay was really performed to demonstrate that the purified AN-PEP was active or how active it was. Please demonstrate the specific activity of AN-PEP and preferably active site titrate both enzymes.

Citations are a bit selective and I have found a couple of references that should have been considered, especially the work on AN-PEP, e.g. more works from F. Koning group, early work of Marti et al . Also the work from Stefanolo (such as 2024, World J Gastroenterol) should have been commented in the Discussion as it deals with CD patients already on the gluten free diet (a randomized double-blind study), as this offers another application for the enzyme.

A minor point. Suppl. Fig. 1. In determining the specificity of celiacase, the authors used PICS and tryptic and GluC peptides. Could the authors explain the increased appearance of glutamates in the Ice logo of Clc-XGR3 in the P2'-P4' positions in the GluC peptides, a pattern not observed in any other group.

Referee #2:

1. The figures require improvement: the font size is too small and the chart labels are largely illegible.

We agree with the reviewer and have thoroughly revised all panels of the main figures. All labels, graphs, and annotations have been updated to a single, consistent sans-serif font to ensure clear readability, even at relatively small letter sizes.

2. All in vivo studies were performed in mice; therefore, the use of rat macrophages for testing (Fig. 3) is puzzling. Substituting mouse macrophages might facilitate the interpretation of data obtained from animal experiments.

We would like to thank the reviewer for pointing out this important observation. In order to solve this obstacle in understanding, we have now performed new experiments using mouse macrophages (Figure 3b). This way, now Figure 3 is a multispecies representation of decrease in the immune response generated by the fragments resulting from the digestion of the 33-mer peptide by celiacase. We now show a panel containing the secretion of cytokines by mouse and rat macrophages and human biopsies. Part of the results on rat macrophages has been consequently moved to the Appendix Figure S6. Moreover, we would like to justify our previous use of rat macrophages in accordance with the principles of the 3Rs (Replacement, Reduction, and Refinement) in animal experimentation. The female rats used in this study were healthy rats aimed to other tissue sampling purposes. The figure now, with mice, rats and humans is more complete. Thus, thanks for the suggestion.

3. Lines 427-428: the statement "By contrast, the fragments did not significantly differ from vehicle-except for IL-2, indicating a markedly reduced immunogenic profile" does not correspond to the data presented in Fig. 3i and j.

We also very much appreciate this point and have revised the paragraph describing the effects of stimulation of biopsies from CD patients with the 33-mer peptide and 33-mer–derived fragments to more accurately reflect the observed results and their implications.

4. Line 558: the statement "Moreover, Clc 1:75 prevented the significant reduction in IgG2b observed in the GLI group relative to REF" does not correspond to the data presented in Fig. 5a.

We apologize for this mistake. We have now deleted this sentence since it was actually confusing and not representative of the results reported.

5. Line 556: the statement "At the intestinal level, most Ig isotypes were increased in GLI compared with REF, particularly in males, an effect prevented by Clc treatment at both doses (Fig. 5f)" is not supported by the data shown in the corresponding figure. How do the authors explain the significant increase in IgG1 following celiacase treatment?

The agree on that this sentence is probably somewhat too overreaching. Accordingly, we have now changed it in order to mention specifically only those Ig isotypes in which a significant increase in GLI compared to REF was observed. Moreover, we have further detailed in which cases Celiacase exerted a preventive effect. In terms of the observed increase in intestinal IgG1 following enzyme treatment, we hypothesize a change in the nature of the mucosal immune response to gluten. Given that IgG1 is associated with Th2-biased humoral immunity in mice, this finding may indicate altered antigen processing and immune recognition in the presence of enzymatically degraded gluten. However, the functional implications of this increase remain to be fully elucidated.

6. Line 585: the statement "Intestinal concentrations of three key pro-inflammatory cytokines associated with CD-IFN-γ, IL-6, and TNF-α-were significantly elevated in GLI mice compared with REF as a result of gliadin intake (Fig. 6a-c). These increments were prevented by Clc treatment,

specifically for IFN-γ and TNF-α in males and IL-6 in females" does not correspond to the presented data, as GLI appears to strongly induce these inflammatory markers only in males. How do the authors explain the striking sex-dependent differences?

We thank the reviewer for this comment. We have now adjusted the text to specifically say that IFN-γ and TNF-α increase in males, while IL-6 increases in females. The sex-specific cytokine differences observed following enzyme treatment may reflect the well-established sexual dimorphism in mucosal immune regulation. Sex hormones and sex-linked factors are known to differentially modulate cytokine networks, with males often exhibiting stronger Th1-associated responses (such as IFN-γ and TNF-α) [1], while females may show enhanced IL-6 production under comparable immune stimulation [2]. In addition, it should be noted that this is a relatively recent animal model—reported in 2020—, for which a full immunological characterization—particularly with respect to sex-dependent responses—is still pending. Therefore, the observed differences may also be linked to intrinsic, sex-derived specificities of the model itself rather than reflecting divergent disease outcomes.

[1] Lasrado, N., Jia, T., Massilamany, C. *et al.* Mechanisms of sex hormones in autoimmunity: focus on EAE. *Biol Sex Differ* **11**, 50 (2020). https://doi.org/10.1186/s13293-020-00325-4
[2] Lamason, R., Zhao, P., Rawat, R. *et al.* Sexual dimorphism in immune response genes as a function of puberty. *BMC Immunol* **7**, 2 (2006). https://doi.org/10.1186/1471-2172-7-2

7. Line 608: „CD8α^{-}CD4^{+} double-positive T cells" should be corrected to CD8α^{+}CD4^{+}.

Thanks for pointing this out, it has been corrected.

Referee #3:

1. Across the manuscript, enzyme dosing is expressed variously as w/w ratios, mg/mL concentrations, and sometimes in mg enzyme without reference to gliadin mass. This inconsistency makes comparison across experiments difficult. The SIMGI enzyme:gliadin ratios (1:250 and 1:50) are not mathematically consistent with the reported enzyme concentrations (0.16 mg/mL; 0.80 mg/mL). Please provide the full dose calculation and include a table expressing all doses as mg enzyme per g gliadin and mg per typical human meal. Also, because this data could be very useful for defining the quantities for an oral formulation.

We thank the reviewer for this valuable feedback. As suggested, we have created new Appendix Table S4 to standardize the dosing information across all experiments. This table provides the full dose calculations and explicitly expresses all doses as mg enzyme per g gliadin and it details the absolute gliadin content for each substrate used in this research. It also reports the SIMGI enzyme:gliadin ratios and their corresponding concentrations.

2. There is no evaluation of off-target digestion or meal-dependent buffering. Please test Clc against representative food and host proteins (mucin, albumin, casein, soy) at gastric pH, or discuss this limitation. A minimal off-target panel is strongly recommended:
a.mucin
b.albumin
c.casein
d. soy proteins
e. representative complex meals with high fat or protein content (high buffering capacity).

We also greatly appreciate the reviewer's recommendation and performed all the assays requested and added panels b and c to new Appendix Figure S1. Specifically, panel S1b now demonstrates the enzymatic activity of Clc against casein, albumin, and soy proteins. In contrast, no off-target activity

against gastric mucin II was found, thus supporting host safety. Furthermore, panel 1Sc now presents an ELISA performed with the G12 mAb, which reveals drastic reduction of GIPs and thus underlies the efficacy of the enzyme even within a complex meal. We further have updated the Results and Discussion sections to reflect these new exciting data.

3. Figures 2i-j appear to show more data points than the stated triplicates. Please clarify n for biological and technical replicates and whether multiple time-points or sampling ports were included.

We thank the reviewer for pointing this out. The inconsistency was due to a plotting error, following which some data points were inadvertently omitted. We have updated Figure 2i-j to include all replicates.

4. In vivo peptide degradation (Section 2.7) lacks fundamental dosing information: total gliadin consumed, relevance to human meal loads, and whether digestion was assessed with complex food matrices.

We fully agree with the reviewer also on this one. We have now added information regarding the volume and concentration of gliadin administered, as well as the human equivalent doses, in Section 2.7. Prior to the experiment, mice had access to food *ad libitum* until one hour before the procedure, in order to standardize gastrointestinal contents. As a result, a complex food matrix (mouse chow) was present during the digestion process, providing a physiologically relevant context for in vivo peptide degradation. In addition, as stated in #1, we have prepared new Appendix Table S4 to standardize the dosing information across all experiments.

5. The reported female-selective histological improvement is not supported by the cited literature. Line 550 refers to an improvement in histological damage, especially in female mice. The discussion (lines 855-857) refers to possible differences between the sexes. While this is true, the cited article doesn't mention any gender differences in the degree of villous atrophy (nor do more recent articles such as doi:10.3390/nu14153192). So, how do you think this can be explained?

There is indeed a divergence between the data and the explained results, we apologize for this mistake. The results actually show a very similar gluten-derived histological damage in both male and female animals, and a likewise preventive effect exerted by Clc. We have, hence, corrected the interpretation of the results in Section 2.8 and in the Discussion.

Referee #4:

1. Authors consistently use the term protease concentration and it seems that they are referring to protein concentration rather than to the active concentration of the protease, which may differ drastically. This is normally determined with active site titration, which was not performed here. While it seems that celiacase (Clc) has considerable activity based on the assays performed with pure enzyme, there is zero evidence of the specific activity of the Aspergillus niger prolyl endopeptidase (AN-PEP) which they used as a comparison. They have purified it from GliadinX (it is GliadinX not Giadin X - please correct in the legend) capsules as shown in Fig 2c, but no specific assay was really performed to demonstrate that the purified AN-PEP was active or how active it was. Please demonstrate the specific activity of AN-PEP and preferably active site titrate both enzymes.

We thank the reviewer for raising this important point. The manuscript has been revised to specify‘protein concentration’ where appropriate, and additional data have been included to validate the potency of the commercial AN-PEP preparation used for comparison with Clc. Specifically, two new panels have been added to Appendix Figure S4, characterizing the activity of AN-PEP purified from *GliadinX*. Panel S4a shows the specific activity measured against the synthetic substrate Z-Gly-Pro-pNA, from which we determined a value of 6.8 U per capsule. Panel S4b further demonstrates

enzymatic activity against the immunogenic FS6 QPQL-based peptide. Regarding dosage, although the manufacturer reports a content of 360 mg (194,000 PPI)—corresponding to approximately 11 U—our functional assays provide a standardized reference to ensure that comparisons between Clc and AN-PEP are based on active enzymatic units. Finally, the spelling of ‘GliadinX’ has been corrected throughout the manuscript and figure legends.

2. Citations are a bit selective and I have found a couple of references that should have been considered, especially the work on AN-PEP, e.g. more works from F. Koning group, early work of Marti et al . Also the work from Stefanolo (such as 2024, World J Gastroenterol) should have been commented in the Discussion as it deals with CD patients already on the gluten free diet (a randomized double-blind study), as this offers another application for the enzyme.

We apologize for this omission. In the revised manuscript, we have expanded the citation base to better reflect prior and recent work on AN-PEP. Specifically, we have added three additional references from the Koning group, including the pioneering study by Stepniak et al. (2006), as well as the early work by Marti et al., and have integrated these studies into the discussion in an appropriate contextual framework. In addition, the study by Stefanolo et al. (2024, *World Journal of Gastroenterology*) has now been included and discussed explicitly four times.

3. A minor point. Suppl. Fig. 1. In determining the specificity of celiacase, the authors used PICS and tryptic and GluC peptides. Could the authors explain the increased appearance of glutamates in the Ice logo of Clc-XGR3 in the P2'-P4' positions in the GluC peptides, a pattern not observed in any other group.

We thank the reviewer for spotting this discrepancy. Upon re-examination, we discovered that two PICS profiles were accidentally swapped during the final assembly of the figure panels. We have now corrected this in the revised version of the manuscript, as well as in new Figure 1k–n.

23rd Mar 2026

Dear Prof. Gomis-Rüth,

Thank you for submitting your revised study. We have now received the reports from the referees who were asked to evaluate your revised manuscript. As you will see below, they are satisfied with the revisions, and I will therefore be able to accept your manuscript once the following minor editorial matters are addressed:

1/ Manuscript text:
- Please indicate any new modification in track changes mode.
- Please correct the order and the headings of the sections in the manuscript text as follows: Abstract / The Paper Explained / Introduction / Results / Discussion / Methods / Data Availability / Acknowledgements / Disclosure and Competing Interests Statement / References / Figure Legends.
- "Material and Methods" should be renamed "Methods".
o Please upload the reagents and tools as a separate file and remove it from the manuscript text
o Please indicate whether the cells were tested for mycoplasm contamination.
o If collected and within the bounds of privacy constraints report on age, sex and gender or ethnicity for all study participants.
o Please provide antibody dilutions
o Statistics: pelase provide statements on blinding and randomization
- Please rename "Availability of data and materials" to "Data availability". Remove "All materials and reagents are available from the authors upon reasonable request". Please provide a URL to the deposited data.
- Funding should be merged with Acknowledgments. Please enter the complete funding information line-by-line and don't use the comments field. The funding information will be made publicly available on PubMed based on the line-by-line entries, so it is important that it is complete and accurate.
- Author contributions: Please remove from the manuscript text, and instead provide CRediT (Contributor Role Taxonomy) terms in the submission system. These replace a narrative author contribution section in the manuscript.
- Remove "Materials and Correspondence".

2/ Figures and Appendix:
- We replaced Supplementary Information with Expanded View (EV) Figures and Tables that are collapsible/expandable online. We would suggest making some of your Appendix figures into EV Figures, that should be cited as 'Figure EV1, Figure EV2" etc... in the text and their respective legends should be included in the main text after the legends of regular figures. For the figures that you do NOT wish to display as Expanded View figures, they should be bundled together with their legends in a single PDF file called *Appendix*, which should start with a short Table of Content. Appendix figures should be referred to in the main text as: "Appendix Figure S1, Appendix Figure S2" etc. Additional Tables/Datasets should be labeled and referred to as Table EV1, Dataset EV1, etc. Legends have to be provided in a separate tab in case of .xls files. Alternatively, the legend can be supplied as a separate text file (README) and zipped together with the Table/Dataset file.
- Figures should be removed from the manuscript text. Legend should be moved to the end of the manuscript text, after References.
- A callout is missing for Fig 4B.
- Appendid Figure S15 is missing in the table of contents.
- Please note that figure reuse is allowed, but should be indicated in the figure legends (i.e. Figure 1Q and Appendix Figure S4C, Figure 2D and Appendix Figure S4C).
- For n=2, please only indicate individual points, and remove error bars and statistics.
- Please address the queries from our data editors in the figure legends:
1. Please note that the exact p values are not provided in the legends of figures 1j; 2a, b, f, j; 3b-d; 4b, d-l; 5a-f; 6a-c, h-k; 7b
2. Please indicate the statistical test used for data analysis in the legends of figures 5c, d, g-j; 6d-g; 7c
3. Please note that the box plots need to be defined in terms of minima, maxima, centre, bounds of box and whiskers, and percentile in the legends of figures 8a, b
4. Please note that information related to n is missing in the legends of figures 1f-j
5. Please note that the error bars are not defined in the legends of figures 1j
6. Please note that the scale bar is missing for figures 4m

3/ Please fill in the author checklist. Information in the checklist should be reflected in the manuscript. The completed author checklist will also be part of the RPF.

4/ Please note that all corresponding authors are required to supply an ORCID ID for their name upon submission of a revised manuscript. An ORCID identifier is currently missing for Francisco Jose Perez-Cano.

5/ Thank you for providing Source Data. Please note that the files provided for Figure 3D and 3E are the same. For the blots, please add annotations.

6/ Thank you for providing the Paper Explained. I have slightly shortened it and structured in into 3 parts to match our format. Please let me know if you agree with the following or amend as you see fit:
Medical issue
Coeliac disease (CD) is a common autoimmune disorder triggered by the ingestion of gluten, a group of proteins present in wheat, barley, and rye. In sensitive individuals, digestion of gluten generates highly resistant fragments known as gluten immunogenic peptides (GIPs). These peptides can activate the immune system and lead to chronic inflammation and damage to the small intestinal lining, causing symptoms such as malabsorption, gastrointestinal discomfort, and systemic complications. Currently, the only effective treatment is strict lifelong adherence to a gluten-free diet (GFD). However, maintaining such a diet is challenging because gluten is widespread in processed foods and accidental exposure is frequent, meaning that many patients continue to experience symptoms despite careful dietary control.
Results
We developed a novel enzyme, termed celiacase (Clc), designed to break down harmful gluten peptides during digestion. The enzyme was engineered from a digestive enzyme found in carnivorous pitcher plants and optimized to improve its stability, activity, and production yield. Biochemical and structural analyses showed that Clc efficiently cleaves GIPs under acidic conditions similar to those in the human stomach. The enzyme degraded the highly immunogenic 33-amino-acid peptide and reduced detectable GIP levels by up to 99% in complex substrates. When tested in immune cells and in duodenal biopsy samples from CD patients, the Clc-generated peptide fragments failed to induce the pro-inflammatory cytokine responses typically triggered by intact GIPs. The enzyme also remained active under simulated human digestive conditions, efficiently degrading gluten during the gastric phase of digestion. Oral administration of the enzyme reduced immunogenic gluten peptides in the gastrointestinal tract of standard mice. In the currently most advanced CD transgenic mouse model, which reproduces key features of the disease, Clc treatment alleviated several hallmarks of the disorder, including intestinal inflammation, villous atrophy, disease-associated antibodies, and gluten-induced alterations in gut microbiota.
Clinical impact:
Together, these findings indicate that Clc is a potent gastric gluten-degrading enzyme capable of neutralizing GIPs before they can trigger harmful immune responses. With further development and clinical evaluation, this strategy could complement the GFD and help protect individuals with CD from accidental gluten exposure.

7/ Synopsis: Thank you for providing the visual abstract. To make it clearer, I suggest modifying the bottom right part (see attached) by changing the arrows or the tick/cross mark.

8/ As part of the EMBO Publications transparent editorial process initiative (see our Editorial at http://embomolmed.embopress.org/content/2/9/329), EMBO Molecular Medicine will publish online a Review Process File (RPF) to accompany accepted manuscripts.
In the event of acceptance, this file will be published in conjunction with your paper and will include the anonymous referee reports, your point-by-point response and all pertinent correspondence relating to the manuscript. Let us know whether you agree with the publication of the RPF.
Please note that the Authors checklist will be published at the end of the RPF.

I look forward to receiving your revised manuscript.

Yours sincerely,

Lise Roth

Lise Roth, PhD
Senior Editor
EMBO Molecular Medicine

***** Reviewer's comments *****

Referee #3 (Remarks for Author):

Excellent work that provides hope in the treatment of celiac disease with a targeted enzymatic approach. The revisions make it clearer and richer in content. It's ready for publication!

Referee #4 (Remarks for Author):

The authors have positively addressed all my concerns.

The authors addressed the remaining editorial issues.

8th Apr 2026

Dear Prof. Gomis-Rüth,

We are pleased to inform you that your manuscript is accepted for publication and is now being sent to our publisher to be included in the next available issue of EMBO Molecular Medicine.

Your manuscript will be processed for publication by EMBO Press. It will be copy edited and you will receive page proofs prior to publication. Please note that you will be contacted by Springer Nature Author Services to complete licensing and payment information.

You may qualify for financial assistance for your publication charges - either via a Springer Nature fully open access agreement or an EMBO initiative. Check your eligibility: https://link.springer.com/journal/44321/how-to-publish-with-us

Should you be planning a Press Release on your article, please get in contact with embo_production@springernature.com as early as possible in order to coordinate publication and release dates.

If you have any questions, please do not hesitate to contact the Editorial Office. Thank you for your contribution to EMBO Molecular Medicine.

Yours sincerely,

Lise

Lise Roth, Ph.D
Senior Editor
EMBO Molecular Medicine

>>> Please note that it is EMBO Molecular Medicine policy for the transcript of the editorial process (containing referee reports and your response letter) to be published as an online supplement to each paper. If you do NOT want this, you will need to inform the Editorial Office via email immediately. More information is available here: https://link.springer.com/partners/embo-press/editorial-policies#Peer%20review

EMBO Press Author Checklist

Corresponding Author Name: Gomis-Rüth, F. Xavier	
Journal Submitted to: EMBO Molecular Medicine	
Manuscript Number: EMM-2025-22961	

USEFUL LINKS FOR COMPLETING THIS FORM
The EMBO Journal - Author Guidelines
EMBO Reports - Author Guidelines
Molecular Systems Biology - Author Guidelines
EMBO Molecular Medicine - Author Guidelines

Reporting Checklist for Life Science Articles (updated January
This checklist is adapted from Materials Design Analysis Reporting (MDAR) Checklist for Authors. MDAR establishes a minimum set of requirements in transparent reporting in the life sciences (see Statement of Task: 10.31222/osf.io/9sm4x). Please follow the journal's guidelines in preparing your
Please note that a copy of this checklist will be published alongside your article.

Abridged guidelines for figures
1. Data
The data shown in figures should satisfy the following conditions:

- the data were obtained and processed according to the field's best practice and are presented to reflect the results of the experiments in an accurate and unbiased manner.
- ideally, figure panels should include only measurements that are directly comparable to each other and obtained with the same assay.
- plots include clearly labeled error bars for independent experiments and sample sizes. Unless justified, error bars should not be shown for technical
- if n<5, the individual data points from each experiment should be plotted. Any statistical test employed should be justified.
- Source Data should be included to report the data underlying figures according to the guidelines set out in the authorship guidelines on Data

2. Captions
Each figure caption should contain the following information, for each panel where they are relevant:

- a specification of the experimental system investigated (eg cell line, species name).
- the assay(s) and method(s) used to carry out the reported observations and measurements.
- an explicit mention of the biological and chemical entity(ies) that are being measured.
- an explicit mention of the biological and chemical entity(ies) that are altered/varied/perturbed in a controlled manner.
- the exact sample size (n) for each experimental group/condition, given as a number, not a range;
- a description of the sample collection allowing the reader to understand whether the samples represent technical or biological replicates (including how many animals, litters, cultures, etc.).
- a statement of how many times the experiment shown was independently replicated in the laboratory.
- definitions of statistical methods and measures:
  - common tests, such as t-test (please specify whether paired vs. unpaired), simple $\chi 2$ tests, Wilcoxon and Mann-Whitney tests, can be unambiguously identified by name only, but more complex techniques should be described in the methods section;
  - are tests one-sided or two-sided?
  - are there adjustments for multiple comparisons?
  - exact statistical test results, e.g., P values = x but not P values < x;
  - definition of 'center values' as median or average;
  - definition of error bars as s.d. or s.e.m.

Please complete ALL of the questions below.
Select "Not Applicable" only when the requested information is not relevant for your study.

Materials

Newly Created Materials	Information included in the manuscript?	In which section is the information available? (Reagents and Tools Table, Materials and Methods, Figures, Data Availability Section)
New materials and reagents need to be available; do any restrictions apply?	Not Applicable	

Antibodies	Information included in the manuscript?	In which section is the information available? (Reagents and Tools Table, Materials and Methods, Figures, Data Availability Section)
For **antibodies** provide the following information:<br>- Commercial antibodies: RRID (if possible) or supplier name, catalogue number and or/clone number<br>- Non-commercial: RRID or citation	Yes	Reagents and tools table, Materials and Methods

DNA and RNA sequences	Information included in the manuscript?	In which section is the information available? (Reagents and Tools Table, Materials and Methods, Figures, Data Availability Section)
**Short novel DNA or RNA including primers, probes**: provide the sequences.	Yes	Methods, Section 4.1

Cell materials	Information included in the manuscript?	In which section is the information available? (Reagents and Tools Table, Materials and Methods, Figures, Data Availability Section)
**Cell lines:** Provide species information, strain. Provide accession number in repository **OR** supplier name, catalog number, clone number, and/**OR** RRID.	Not Applicable	
**Primary cultures:** Provide species, strain, sex of origin, genetic modification status.	Yes	Reagents and tools table, Materials and Methods
Report if the cell lines were recently **authenticated** (e.g., by STR profiling) and tested for mycoplasma contamination.	Not Applicable	

Experimental animals	Information included in the manuscript?	In which section is the information available? (Reagents and Tools Table, Materials and Methods, Figures, Data Availability Section)
**Laboratory animals or Model organisms:** Provide species, strain, sex, age, genetic modification status. Provide accession number in repository **OR** supplier name, catalog number, clone number, **OR** RRID.	Yes	Reagents and tools table, Materials and Methods
**Animal observed in or captured from the field:** Provide species, sex, and age where possible.	Not Applicable	
Please detail **housing and husbandry conditions**.	Yes	Reagents and tools table, Materials and Methods

Plants and microbes	Information included in the manuscript?	In which section is the information available? (Reagents and Tools Table, Materials and Methods, Figures, Data Availability Section)
**Plants:** provide species and strain, ecotype and cultivar where relevant, unique accession number if available, and source (including location for collected wild specimens).	Not Applicable	
**Microbes:** provide species and strain, unique accession number if available, and source.	Not Applicable	

Human research participants	Information included in the manuscript?	In which section is the information available? (Reagents and Tools Table, Materials and Methods, Figures, Data Availability Section)
If collected and within the bounds of privacy constraints report on age, sex and gender or ethnicity for all study participants.	Yes	Reagents and tools table, Materials and Methods

Core facilities	Information included in the manuscript?	In which section is the information available? (Reagents and Tools Table, Materials and Methods, Figures, Data Availability Section)
If your work benefited from core facilities, was their service mentioned in the acknowledgments section?	Yes	Reagents and tools table, Materials and Methods

Design

Study protocol	Information included in the manuscript?	In which section is the information available? (Reagents and Tools Table, Materials and Methods, Figures, Data Availability Section)
If study protocol has been **pre-registered, provide DOI in the manuscript**. For clinical trials, provide the trial registration number **OR** cite DOI.	Not Applicable	
Report the **clinical trial registration number** (at ClinicalTrials.gov or equivalent), where applicable.	Not Applicable	

Laboratory protocol	Information included in the manuscript?	In which section is the information available? (Reagents and Tools Table, Materials and Methods, Figures, Data Availability Section)
Provide DOI OR other citation details if **external detailed step-by-step protocols** are available.	Not Applicable	

Experimental study design and statistics	Information included in the manuscript?	In which section is the information available? (Reagents and Tools Table, Materials and Methods, Figures, Data Availability Section)
Include a statement about **sample size** estimate even if no statistical methods were used.	Yes	Reagents and tools table, Materials and Methods
Were any steps taken to minimize the effects of subjective bias when allocating animals/samples to treatment (e.g. **randomization procedure**)? If yes, have they been described?	Yes	Reagents and tools table, Materials and Methods
Include a statement about **blinding** even if no blinding was done.	Yes	Materials and methods
Describe **inclusion/exclusion criteria** if samples or animals were excluded from the analysis. Were the criteria pre-established?<br><br>If sample or data points were omitted from analysis, report if this was due to attrition or intentional exclusion and provide justification.	Not Applicable	
For every figure, are **statistical tests** justified as appropriate? Do the data meet the assumptions of the tests (e.g., normal distribution)? Describe any methods used to assess it. Is there an estimate of variation within each group of data? Is the variance similar between the groups that are being statistically compared?	Yes	Materials and methods, Figures

Sample definition and in-laboratory replication	Information included in the manuscript?	In which section is the information available? (Reagents and Tools Table, Materials and Methods, Figures, Data Availability Section)
In the figure legends: state number of times the experiment was **replicated** in laboratory.	Yes	Figure legends
In the figure legends: define whether data describe **technical or biological replicates**.	Yes	Figure legends

Ethics

Ethics	Information included in the manuscript?	In which section is the information available? (Reagents and Tools Table, Materials and Methods, Figures, Data Availability Section)
Studies involving **human participants**: State details of **authority granting ethics approval** (IRB or equivalent committee(s), provide reference number for approval.	Yes	Materials and methods
Studies involving **human participants**: Include a statement confirming that **informed consent** was obtained from all subjects and that the experiments conformed to the principles set out in the WMA Declaration of Helsinki and the Department of Health and Human Services Belmont Report.	Yes	Materials and methods
Studies involving **human participants: For** publication of **patient photos**, include a statement confirming that consent to publish was obtained.	Not Applicable	
Studies involving experimental **animals**: State details of **authority granting ethics approval** (IRB or equivalent committee(s), provide reference number for approval. Include a statement of compliance with ethical regulations.	Yes	Materials and methods
Studies involving **specimen and field samples:** State if relevant **permits** obtained, provide details of authority approving study; if none were required, explain why.	Not Applicable	

Dual Use Research of Concern (DURC)	Information included in the manuscript?	In which section is the information available? (Reagents and Tools Table, Materials and Methods, Figures, Data Availability Section)
Could your study fall under dual use research restrictions? Please check biosecurity documents and list of **select agents and toxins** (CDC): https://www.selectagents.gov/sat/list.htm	Not Applicable	
If you used a select agent, is the security level of the lab appropriate and reported in the manuscript?	Not Applicable	
If a study is subject to dual use research of concern regulations, is the name of the **authority granting approval and reference number** for the regulatory approval provided in the manuscript?	Not Applicable	

Reporting

The MDAR framework recommends adoption of discipline-specific guidelines, established and endorsed through community initiatives. Journals have their own policy about requiring specific guidelines and recommendations to complement MDAR.

Adherence to community standards	Information included in the manuscript?	In which section is the information available? (Reagents and Tools Table, Materials and Methods, Figures, Data Availability Section)
State if relevant guidelines or checklists (e.g., **ICMJE, MIBBI, ARRIVE, PRISMA**) have been followed or provided.	Yes	ARRIVE, Materials and Methods
For **tumor marker prognostic studies,** we recommend that you follow the **REMARK** reporting guidelines (see link list at top right). See author guidelines, under 'Reporting Guidelines'. Please confirm you have followed these guidelines.	Not Applicable	
For **phase II and III randomized controlled trials**, please refer to the **CONSORT** flow diagram (see link list at top right) and submit the CONSORT checklist (see link list at top right) with your submission. See author guidelines, under 'Reporting Guidelines'. Please confirm you have submitted this list.	Not Applicable	

Data Availability

Data availability	Information included in the manuscript?	In which section is the information available? (Reagents and Tools Table, Materials and Methods, Figures, Data Availability Section)
Have **primary datasets** been deposited according to the journal's guidelines (see 'Data Deposition' section) and the respective accession numbers provided in the Data Availability Section?	Yes	Data availability section
Were **human clinical and genomic datasets** deposited in a public access-controlled repository in accordance to ethical obligations to the patients and to the applicable consent agreement?	Not Applicable	
Are **computational models** that are central and integral to a study available without restrictions in a machine-readable form? Were the relevant accession numbers or links provided?	Not Applicable	
If publicly available data were reused, provide the respective **data citations in the reference list**.	Not Applicable	
